# Effect of histidine protonation state on ligand binding at the ATP-binding site of human protein kinase CK2

**DOI:** 10.1038/s41598-024-51905-y

**Published:** 2024-01-17

**Authors:** Maria Winiewska-Szajewska, Daniel Paprocki, Ewa Marzec, Jarosław Poznański

**Affiliations:** 1 Institute of Biochemistry and Biophysics PAS, Pawinskiego 5a, 02-106 Warsaw, Poland; 2 Division of Biophysics, Institute of Experimental Physics, University of Warsaw, Pasteura 5, 02-089 Warsaw, Poland

**Keywords:** Molecular biophysics, Kinases

## Abstract

Histidine residues contribute to numerous molecular interactions, owing to their structure with the ionizable aromatic side chain with pK_a_ close to the physiological pH. Herein, we studied how the two histidine residues, His115 and His160 of the catalytic subunit of human protein kinase CK2, affect the binding of the halogenated heterocyclic ligands at the ATP-binding site. Thermodynamic studies on the interaction between five variants of hCK2α (WT protein and four histidine mutants) and three ionizable bromo-benzotriazoles and their conditionally non-ionizable benzimidazole counterparts were performed with nanoDSF, MST, and ITC. The results allowed us to identify the contribution of interactions involving the particular histidine residues to ligand binding. We showed that despite the well-documented hydrogen bonding/salt bridge formation dragging the anionic ligands towards Lys68, the protonated His160 also contributes to the binding of such ligands by long-range electrostatic interactions. Simultaneously, His 115 indirectly affects ligand binding, placing the hinge region in open/closed conformations.

## Introduction

Histidine is undoubtedly one of the most versatile residues affecting protein structure and function. The functional importance of this amino acid was proved in numerous proteins^1–5^, including the HRD motif of a protein kinase^6^. Histidine residues play multiple roles in molecular interactions, owing to their structure with the ionizable aromatic imidazole side chain, pK_a_ of which is close to the physiological pH. Depending on the pH conditions, the two protonation forms (neutral and protonated) differ by charge and geometry of the aromatic ring^7^. The neutral form of the aromatic imidazole ring can participate in the cation-π interactions with various metal cations or Lys and Arg residues^8–10^. When protonated, it donates cation-π interactions with other aromatic residues (Phe, Tyr, and Trp)^11,12^. Both histidine forms can also participate in π-π stacking with aromatic side chains of proximal Phe, Tyr, and Trp residues^13,14^, whose contributions differ, however, in strength^15^. The two particular motifs of the imidazole ring are responsible for specific interactions: the polar secondary amine group and the basic nitrogen atoms. The first acts as a hydrogen-bond donor (including hydrogen-π bonds^16^), while the free imino nitrogen is a hydrogen-bond acceptor. Most importantly, proton-free histidine's heterocyclic nitrogen atom is widely involved in coordinating metal cations^17^. Summarizing, each state of histidine interacts specifically, differing by interaction types or for a particular interaction by preferred geometry and strength^15^. However, the explicit contribution of the formal charge to the long-range electrostatic interactions should also be considered.

We use the catalytic domain of human protein kinase CK2 (hCK2α) as the model protein in ligand binding studies^18–20^. CK2 is a serine/threonine protein kinase that plays an essential role in many processes, including cell growth, differentiation, death, and survival, by controlling several signaling pathways^21^. Protein kinase CK2 is implicated in many human pathologies, including neurodegeneration (Parkinson's^22,23^ and Alzheimer’s^24–26^ diseases), hypoxia^27,28^ atherosclerosis^29^ various human infections, and many other medical conditions already extensively reviewed^30,31^. The intense attention as a therapeutic target CK2 attracts in cancer therapy^32^ and has recently been considered a target for SARS-CoV-2 virus treatment^33,34^. For those reasons, CK2 is intensively studied, and numerous inhibitors have already been developed. Most of them represent so-called “Type I” inhibitors that target the ATP-binding site of the protein. An important family of CK2 inhibitors directed towards the conserved ATP binding site constitutes benzimidazole and benzotriazole derivatives, which scaffold derived from the 5,6-dichloro-1-(β-d-ribofuranosyl)benzimidazole^35–38^.

**Figure 1:**
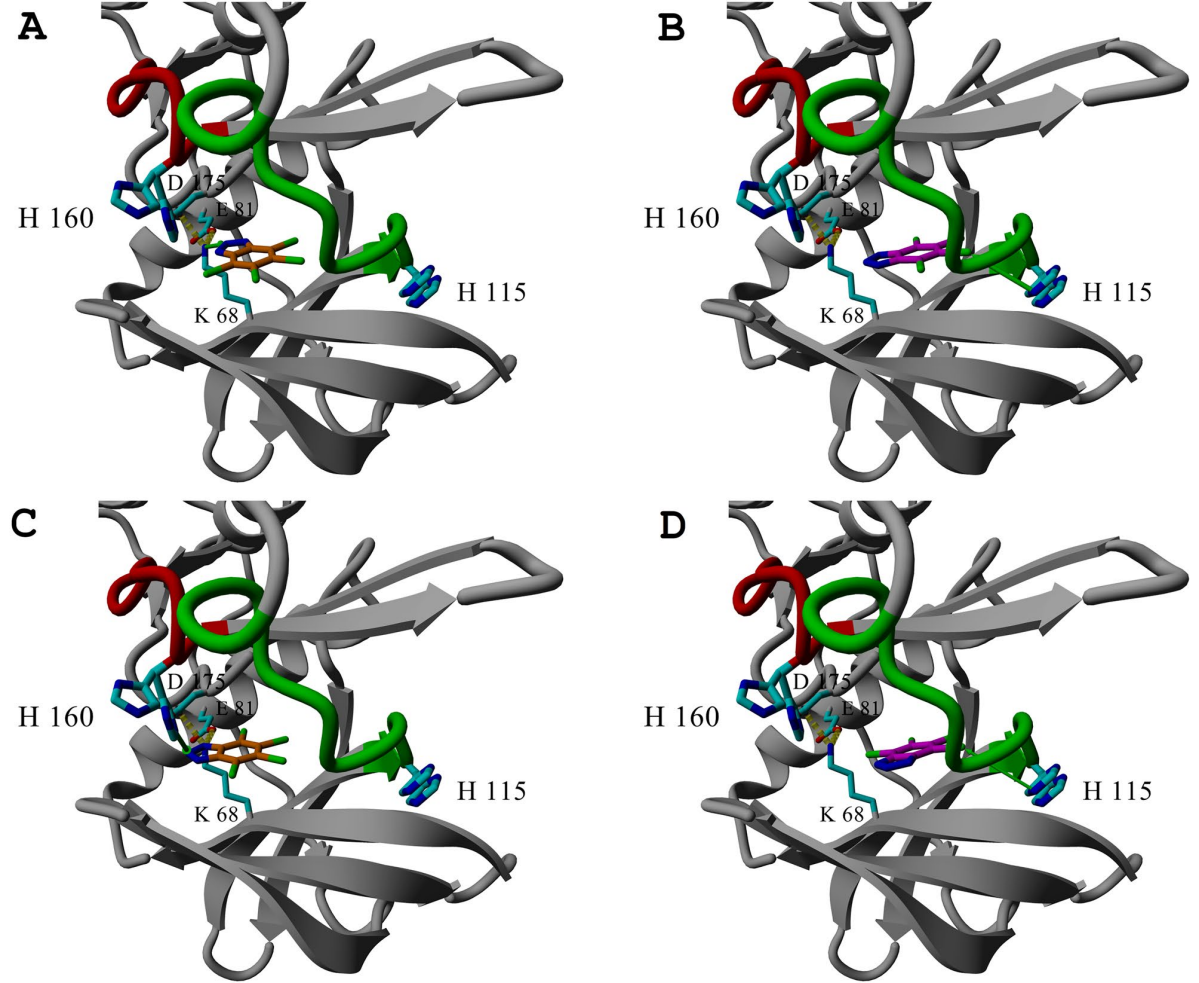
The interaction of TBBt within the binding pocket of hCK2α (PDB:6tll). His115 and His160 are identified in two conformations, and TBBt adopts four alternative poses, of which two proximal to His115 are denoted in magenta/blue, and two closer to His160 are in orange/blue. The green arrows identify the shortest histidine-ligand contacts. The hinge region and the catalytic loop are denoted in green and red, respectively.

We have recently shown that a balance of hydrophobic and electrostatic interactions contribute predominantly to the binding of halogenated benzotriazoles at the ATP-binding site of hCK2α^19,20,39^. Five residues neighboring the CK2 ATP-binding site may contribute to the effective electrostatic potential. Among them, there is positively charged Lys68 from the N-lobe involved in the binding of α- and β-phosphate in the productive complex with the ATP. The geometry of ligands allows the anionic ones to form hydrogen bonds or salt bridges with the Lys68, while neutral ones preferably bind closer to the hinge region^40^. Our recent studies demonstrated that electrostatic interactions predominate over possible halogen/hydrogen bonding with the hinge region of human CK2α^41^. We showed that the apparent binding affinity of titratable 5,6-dibromobenzotriazole (5,6-Br_2_Bt; pK_a_ = 6.93)^42^ to hCK2α varies non-linearly with pH, and the inflection point identifies the dissociation of the triazole proton upon ligand binding^18^. However, a slight inverse trend of pH-dependent changes was observed for 4,5,6,7-tetrabromobenzotriazole (TBBt), in which anionic form predominates in the tested pH range. Such observation indicates an alternative source of variation in the apparent binding affinity, presumably associated with the ionization states of neighboring histidine residues. Two of the sixteen histidine residues are within the 5 Å distance from the bound ligand: His115 from the hinge region and His160 of the catalytic loop (see Fig. 1).

While several studies showed that hydrogen bonding/salt bridge formation drags the ligands towards Lys68^41^, nothing was reported on how His115 and His160 (or their equivalents in other PK) may affect the binding of charged ligands and how such interactions can be used to optimize Type I inhibitors (i.e., those that bind at the ATP-binding site of a protein kinase).

**Figure 2:**
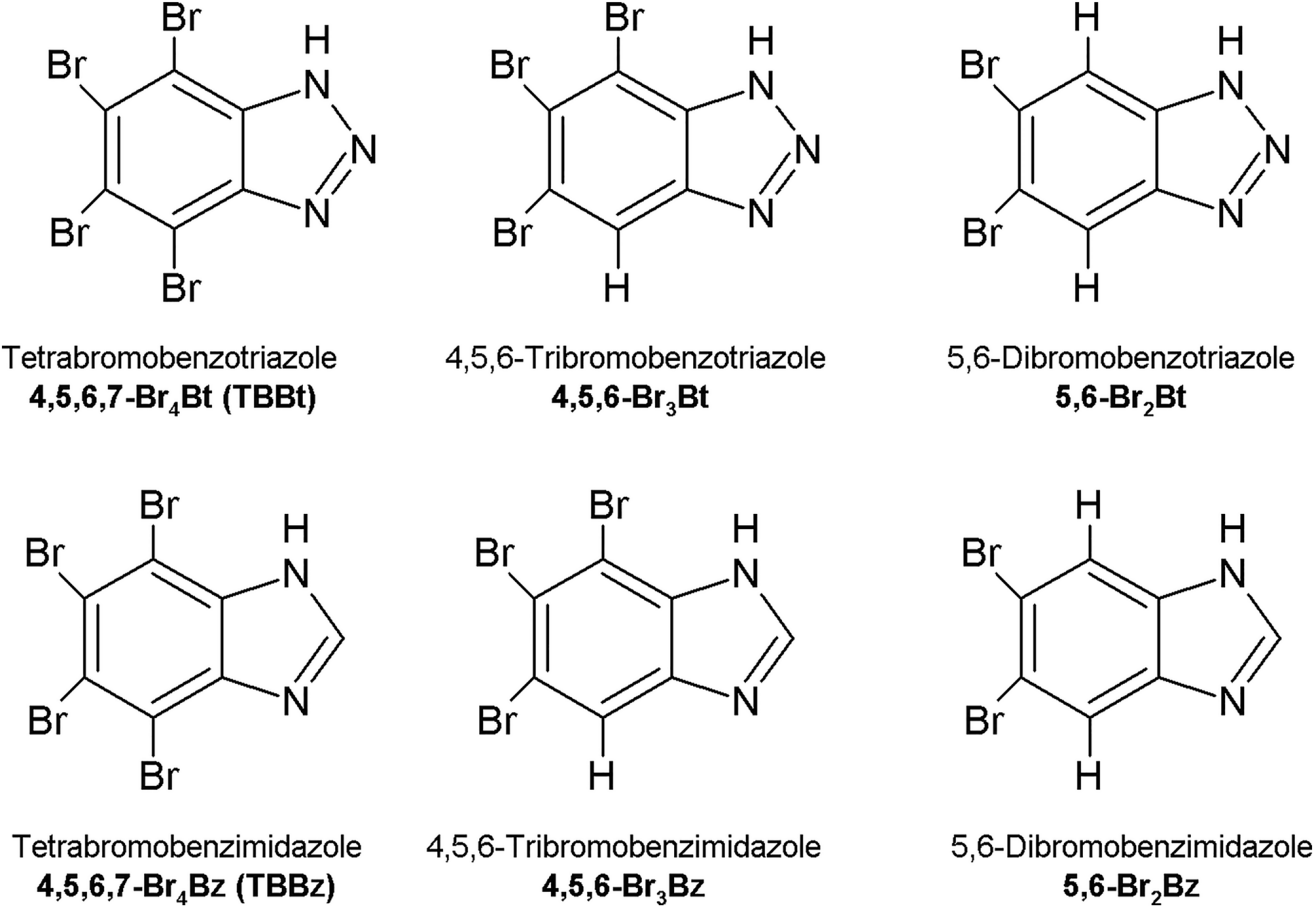
Schematic diagrams of the compounds used in the study.

To address this problem, we studied the interaction between five variants of hCK2α (WT protein and four histidine mutants) and three reference ionizable bromo-benzotriazoles and their conditionally non-ionizable benzimidazole counterparts (see Fig. 2) to identify the contribution of long-range electrostatic interactions involving the particular histidine residue to ligand binding.

## Results and discussion

### CK variants

**Figure 3:**
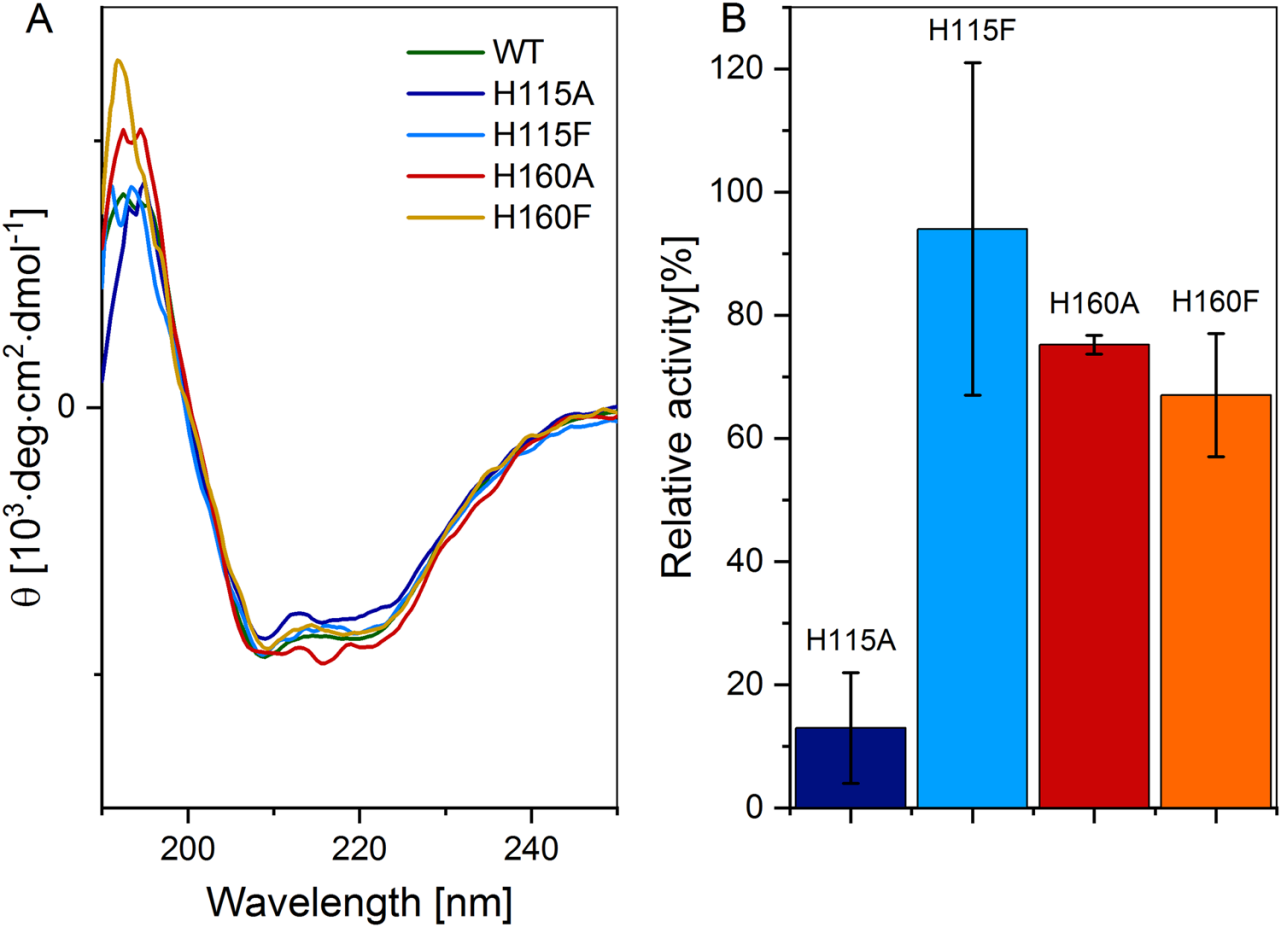
CD Spectra of the studied hCK2α variants (**A**) and their enzymatic activity (**B**).

Besides the WT protein, four variants of hCK2α were analyzed-two in which a single histidine residue was substituted with alanine (H115A and H160A) and two other ones in which phenylalanine was introduced to preserve the existence of the aromatic ring (H115F and H160F). These four replacements were designed based on the in silico analysis of the hCK2α structure (see Fig. 1). All these variants fold correctly, as confirmed with Circular Dichroism spectroscopy (see Fig. 3A).

We also assessed the enzymatic activity of the tested protein variants (Fig. 3B and Table 1). The H115F variant retains the activity of the WT protein, and both H160A and H160F are minutely less active, while H115A displays only vestigial activity. However, the observed decrease in the activity of the H115A variant is likely due to its reduced stability rather than weaker or non-productive substrate binding. We applied differential scanning fluorimetry (DSF) to determine the thermal stability of these four hCK2α variants and, indeed, replacing His115 with alanine shifts the thermal denaturation middle-point temperature from 46.2 down to 38.5 °C with the protein aggregation starting at 35.5 ± 1.0 °C. The replacement effects were additionally assessed in silico with the aid of FoldX. We also checked with the aid of MST that ATP binding affinities for the H160A variant and the WT protein are the same within the estimated experimental uncertainties (K_diss_ = 1.7 ± 0.5 and 2.2 ± 0.4 μM, respectively).

**Table 1:** The stability of WT hCK2α and its four variants, determined with DSF (middle-point denaturation temperature, T_m_) and assessed in silico with FoldX change in free energy of unfolding (ΔΔG_unf_), and relative enzymatic activity.

Protein variant	T_m_ [°C]	T_aggregation_ [°C]	ΔΔG_unf_ [kJ·mol^−1^]	Activity [%] (relative to WT)
WT	46.2 ± 0.1	41.5 ± 0.9	–	100
H115A	38.5 ± 0.1	35.5 ± 1.0	2.5 ± 0.2	13 ± 9
H115F	46.6 ± 0.1	40.9 ± 0.4	− 1.1 ± 0.1	94 ± 27
H160A	48.0 ± 0.1	43.9 ± 0.9	− 5.1 ± 0.1	75 ± 2
H160F	44.4 ± 0.1	39.3 ± 0.4	− 1.0 ± 0.4	67 ± 10

### Ligand binding by His160 and His115 hCK2α mutants

**Figure 4:**
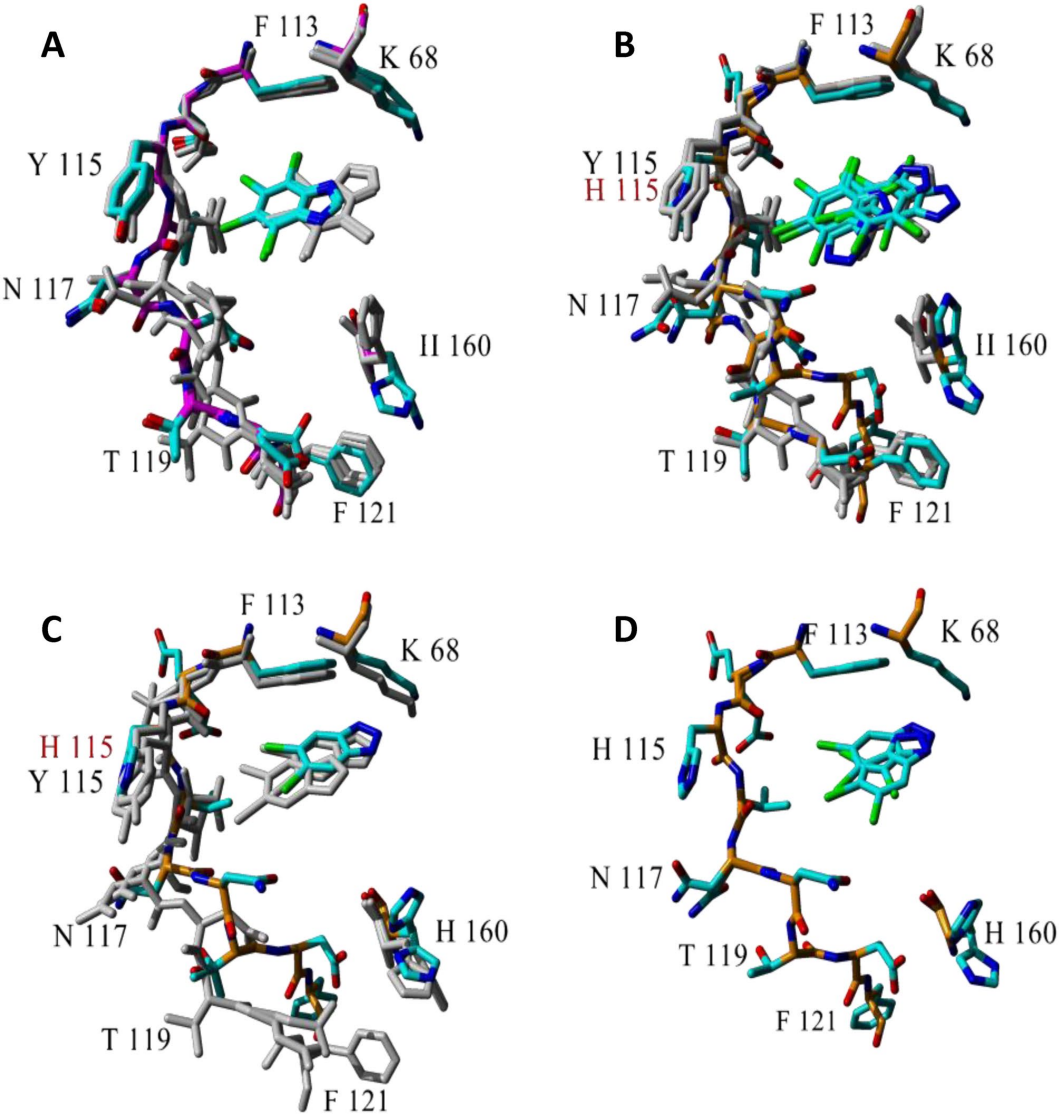
The tested ligands bound at the ATP-ligand site of CK2α. (**A**) the complex of maize CK2α with TBBz (PDB:2oxy) and TBBt (PDB:1j91) as a reference (gray), (**B**) the complex of human CK2α with TBBt (PDB:6tll) and maize CK2α as a reference (gray), (**C**) the complex of human CK2α with 5,6Br_2_Bt (PDB:6tlp) and maize CK2α (PDB:5ts8) as a reference (gray), (**D**) the complex of human CK2α with 4,5,6Br_3_Bt (PDB:6tlo).

We used differential scanning fluorescence (nanoDSF) to study the binding of model ligands by four variants of hCK2α relative to the WT protein. We choose three benzotriazoles (5,6-Br_2_Bt,4,5,6-Br_3_Bt, and 4,5,6,7-Br_4_Bt) that differ in their physicochemical properties together with their three conditionally indissociable benzimidazole analogs (5,6-Br_2_Bz, 4,5,6- Br_3_Bz, 4,5,6,7-Br_4_Bz). Such a ligand series enables sensing protein-dependent changes in the contribution of electrostatic and hydrophobic interactions to ligand binding. The binding poses at the ATP-binding site of hCK2α are known for all three tested benzotriazoles, while for benzimidazoles, the only available crystal structure is of TBBz bound to maize CK2α (see Fig. 4).

According to the analysis of available structures of halogenated ligands with CK2, neutral ligands, irrespective of the organism (here maize or human), bind closer to the hinge, possibly forming halogen bonds with the backbone carbonyl oxygen atoms of residues from the hinge region^18,40^. However, for anionic ligands, there are some slight differences depending on the origin of CK2α. TBBt is bound in a single pose to maize CK2α, forming a salt bridge with Lys68, not halogen bonds with the hinge. By contrast, TBBt in human CK2α is bound in multiple poses, some of which allow for halogen bonding with the hinge region (Fig. 4B). Multiple poses are also observed for 4,5,6-Br_3_Bt bound to hCK2α (Fig. 4D). Interestingly, the opposite relation was identified for 5,6-Br_2_Bt. Thus, the single pose in hCK2α (close to Lys68) is confronted with the two poses for mCK2α, one with halogen bonding to the hinge and another with salt bridge formed to Lys68 in mCK2α.

The binding affinities were initially estimated from the shift of the thermal unfolding profile, ∆T_m_, registered for the protein in *apo* form and complex with a ligand (see Table 2). The observed ∆T_m_ values for H160A are generally lower by 0.5–1 °C than those for WT protein, while subtle effects are observed for other hCK2α variants. It must be, however, mentioned that the nanoDSF method is regarded as semi-quantitative since binding affinities are temperature-dependent, and differences in the thermal stability of each variant might also contribute to ∆T_m_ change. A balance of enthalpic and entropic contributions to the binding may also affect the thermal shift of the complex. Ligands whose binding is entropically driven usually exhibit higher ∆T_m_ than those of the same affinity but with enthalpically driven binding. That is why we only considered the ∆T_m_ variation exceeding 1 °C as an indicator of change in ligand binding affinity.

**Table 2:** Thermal stabilization of four variants of hCK2α by the presence of tenfold excess of tested ligands, assessed by nanoDSF at pH 8.

Ligand	4,5,6,7-Br_4_Bt	4,5,6-Br_3_Bt	5,6-Br_2_Bt	4,5,6,7-Br_4_Bz	4,5,6 -Br_3_Bz	5,6-Br_2_Bz
pK_a_	4.78^42^	5.91^42^	6.93^42^	9.73 ± 0.08	10.75 ± 0.04	11. 77 ± 0.02
Protein variant	ΔT_m_[°C]					
WT	9.0 ± 0.1^39^	8.4 ± 0.1^39^	7.4 ± 0.1^39^	5.1 ± 0.1	4.3 ± 0.1	1.6 ± 0.1
H115A	8.9 ± 0.1	7.6 ± 0.1	7.1 ± 0.1	**3.5 ± 0.1**	3.5 ± 0.1	1.2 ± 0.1
H115F	8.3 ± 0.1	8.0 ± 0.1	7.1 ± 0.1	5.8 ± 0.1	4.4 ± 0.1	2.0 ± 0.1
H160A	8.4 ± 0.1	**6.9 ± 0.1**	6.5 ± 0.1	4.9 ± 0.1	3.5 ± 0.1	0.9 ± 0.1
H160F	9.8 ± 0.1	8.3 ± 0.1	8.0 ± 0.1	4.8 ± 0.1	4.6 ± 0.1	2.0 ± 0.1

Values that changed significantly are in bold.

For both H115 variants, there is no significant difference in ΔT_m_ for benzotriazole derivatives at pH 8. However, for the H115A, but not for H115F, an evident change in TBBz binding affinity is observed. Also, for H160 variants, DSF experiments are minutely contradictory—as for 4,5,6-Br_3_Bt, which stabilizes the H160A by ~ 1.5 °C less than WT protein, when no such difference is observed when the same ligand binds to H160F variant. These discrepancies might stem from different melting temperatures of the *apo* form of hCK2α variants reflecting the temperature dependence of the binding affinity. However, they can also result directly from different physicochemical properties of alanine, phenylalanine, and histidine residues.

These observations were further confirmed with Microscale Thermophoresis (MST) and, additionally, for WT protein and alanine variants, with Isothermal Titration Calorimetry (ITC). Both methods allowed the direct determination of binding affinities and associated thermodynamic parameters for binding at 25 °C (presented in Table 3).

**Table 3:**
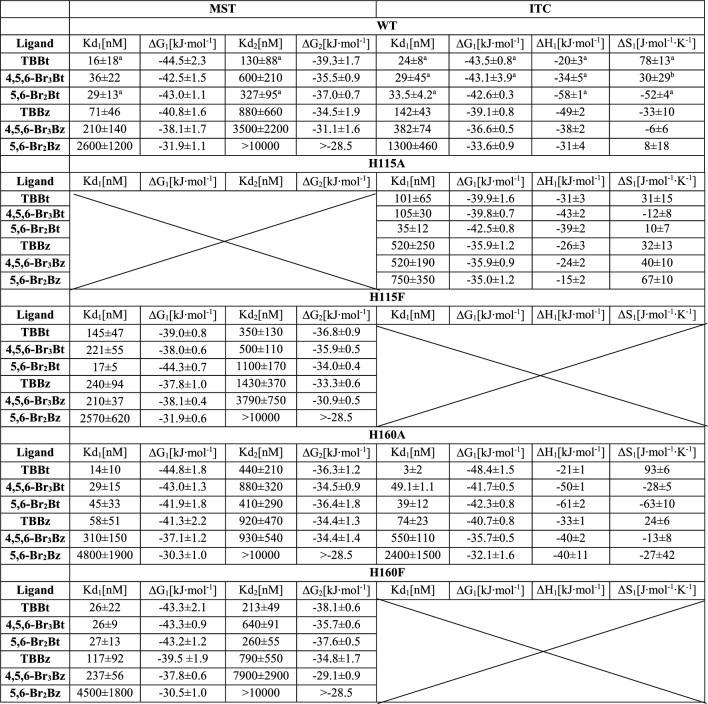
The thermodynamic data for binding events monitored with MST and ITC at pH 8.

^a^Data from Refs.^41,49^ recalculated as described in “Methods”.

As reported previously^19,39^, MST-derived data displayed the second inflection point at a higher ligand concentration, indicating the second weaker binding site. This second transition should be assigned to the binding at the site located at the interface between α and β subunits of CK2, identified in crystal structures of variously brominated benzotriazoles bound by hCK2α^43^.Consequently, all MST data were analyzed according to the model of two independent binding sites. ITC analysis differs depending on ligand–protein systems. The model assuming a single binding site was found sufficient for most ligands, and results obtained with the MST and ITC methods were consistent. However, significant discrepancies between the two methods occurred for the binding of TBBt and 4,5,6-Br_3_Bt by WT, and TBBt binding by the H160A variant. For these interactions studied by ITC, the model of two independent binding sites was scored better according to the F-test. Supplementary Table S2 summarizes the justification for the fitting model selection and thermodynamic parameters for the second binding.

**Figure 5:**
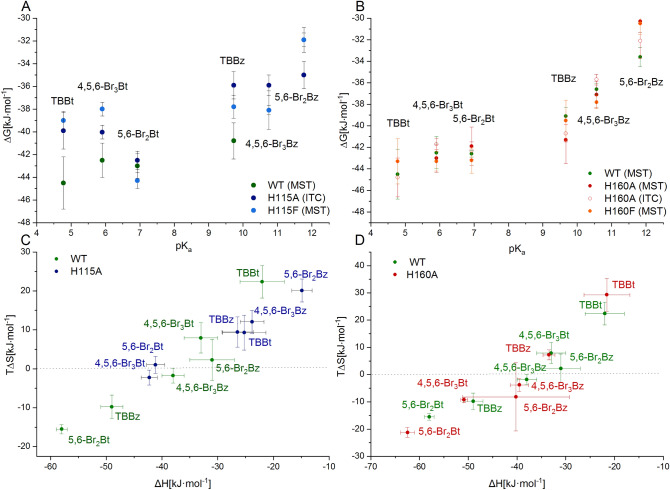
Thermodynamics of the interaction of hCK2α variants with the tested ligands. Binding by the catalytic subunit of human protein kinase CK2 vs. H115 (**A**) and H160 mutants (**B**) was assessed with ITC and MST methods. Additionally, the entropy-enthalpy compensation is compared for WT vs. H115A (**C**) and H160A (D).

Intense aggregation of the H115A variant during the required labeling precluded the application of MST, so for this hCK2α variant, we analyzed ligand binding with ITC. Measured binding affinities for a particular ligand fall for both mutants within the limits of experimental error except for a slight difference in binding 4,5,6-Br_3_Bt. Interestingly, both H115A and H115F bind TBBt, 4,5,6-Br_3_Bt, and TBBz weaker than the WT protein, while their affinity towards 5,6-Br_2_Bt, 5,6-Br_2_Bz and 4,5,6-Br_3_Bz remains virtually unaffected (Fig. 5A). So, the binding affinity of H115 variants decreases for ligands that putatively interact with the hinge region^40,41^. Moreover, the analysis of the enthalpic and entropic components (Fig. 5C) proves that binding of all benzimidazoles to the H115A mutant is much more entropy-driven, while for two benzotriazoles possibly interacting with the hinge region, this trend is reversed. Interestingly, 5,6-Br_2_Bt, which binds distantly to the hinge, follows the trend observed for neutral benzimidazoles.

Upon His160 replacements, the binding affinities of the studied ligands do not significantly change at pH 8 (Fig. 5B), and no significant change is also observed for the enthalpy-entropy balance (Fig. 5D).

### pH-dependent binding

**Figure 6:**
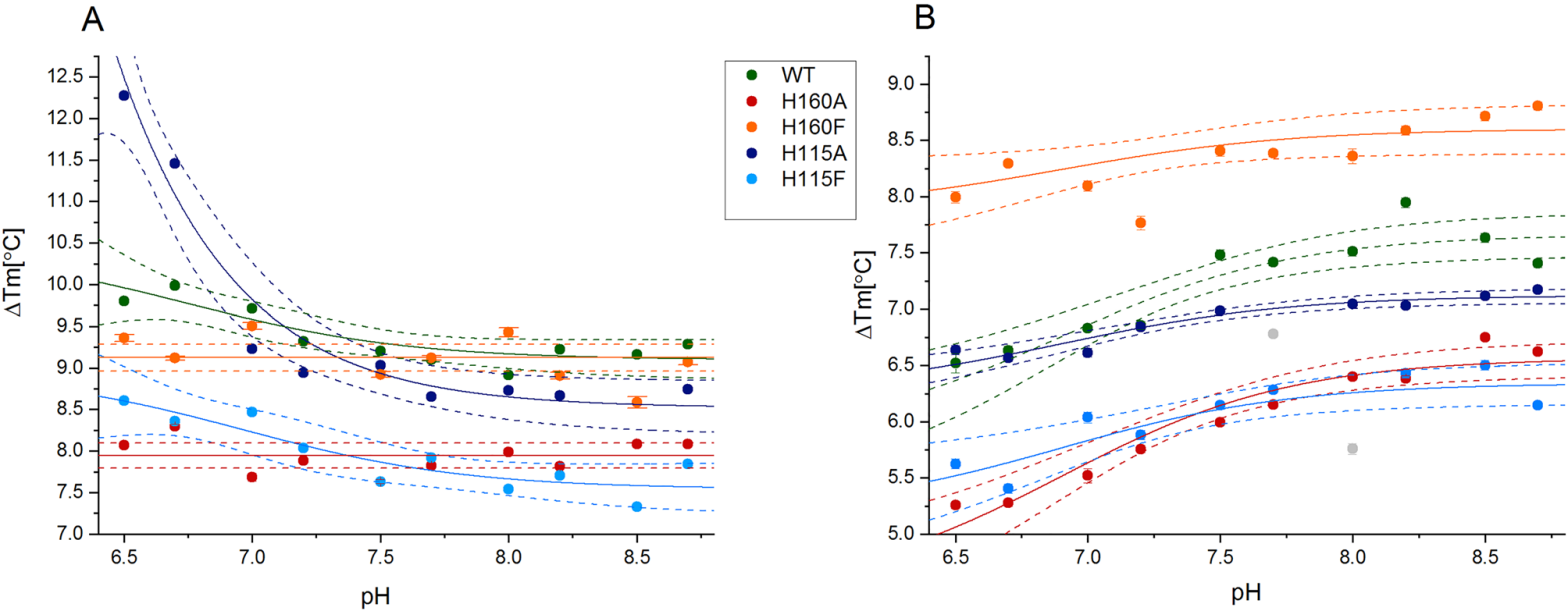
pH-dependence of ΔT_m_ for the complexes of different variants of hCK2α with TBBt (**A**) and 5,6- Br_2_Bt (**B**) determined using nanoDSF.

Our recent study demonstrated that pH-dependent binding of 5,6-Br_2_Bt is due to forced ligand deprotonation upon the binding event^18^. However, a slight opposite trend was observed for TBBt binding, for which anionic form predominates at all tested pH. We had assigned this apparent variation in TBBt binding by WT protein to the pH-induced (de)protonation of the neighboring histidine residue (putatively His160, with a theoretical pK_a_ of ~ 7)^18^. To confirm this hypothesis, we analyzed a series of DSF data collected at different pH for both histidine mutants against the values previously reported for the WT protein (Table 4 and Fig. 6). We verified whether the pH-dependent change in ΔT_m_ can be described by simple sigmoidal relation (1):

1 $${\Delta T}_{m}={\Delta T}_{m0}+\frac{{\Delta \Delta T}_{m}}{1+{10}^{(pX-pH)\cdot p}}$$

where ${\Delta T}_{m0}$ is the estimate of melting temperature unaffected by protonation/deprotonation event, ${\Delta \Delta T}_{m}$ is a difference between ${\Delta T}_{m}$ between two extreme states, pX is the pH of the inflection point, and p is a measure of (de)protonation cooperativity (Hill coefficient).

**Table 4:** Thermal stabilization of four variants of hCK2α by the presence of tenfold excess of tested ligands at different pH, assessed by nanoDSF.

pH	6.5	6.7	7	7.2	7.5	7.7	8	8.2	8.5	8.7
WT^c^										
apo	41.7 ± 0.1	42.4 ± 0.1	43.6 ± 0.1	44.9 ± 0.1	45.4 ± 0.1	46.1 ± 0.1	46.3 ± 0.1	46.4 ± 0.1	46.2 ± 0.1	46.2 ± 0.1
TBBt	9.8 ± 0.1	10.0 ± 0.1	9.7 ± 0.1	9.3 ± 0.1	9.2 ± 0.1	9.1 ± 0.1	8.9 ± 0.1	9.2 ± 0.1	9.2 ± 0.1	9.3 ± 0.1
5,6-Br_2_Bt	6.5 ± 0.1	6.6 ± 0.1	6.8 ± 0.1	6.9 ± 0.1	7.5 ± 0.1	7.4 ± 0.1	7.5 ± 0.1	8.0 ± 0.1	7.6 ± 0.1	7.4 ± 0.1
H160A										
apo	44.5 ± 0.1	45.2 ± 0.1	46.1 ± 0.1	46.3 ± 0.1	47.2 ± 0.1	47.6 ± 0.1	47.4 ± 0.1	47.5 ± 0.1	47.5 ± 0.1	47.5 ± 0.1
TBBt	8.1 ± 0.1	8.3 ± 0.1	7.7 ± 0.1	7.9 ± 0.1	7.6 ± 0.1	7.8 ± 0.1	8.0 ± 0.1	7.8 ± 0.1	8.1 ± 0.1	8.1 ± 0.1
5,6-Br_2_Bt	5.3 ± 0.1	5.3 ± 0.1	5.5 ± 0.1	5.8 ± 0.1	6.0 ± 0.1	6.2 ± 0.1	6.4 ± 0.1	6.4 ± 0.1	6.8 ± 0.1	6.62 ± 0.1
H160F										
apo	38.9 ± 0.1	41.0 ± 0.1	42.3 ± 0.1	43.7 ± 0.1	45.9 ± 0.1	44.6 ± 0.1	45.0 ± 0.1	45.4 ± 0.1	45.0 ± 0.1	45.0 ± 0.1
TBBt	9.4 ± 0.1	9.1 ± 0.1	9.5 ± 0.1	–	8.9 ± 0.1	9.1 ± 0.1	9.4 ± 0.1	8.9 ± 0.1	8.6 ± 0.1	9.1 ± 0.1
5,6-Br_2_Bt	8.0 ± 0.1	8.3 ± 0.1	8.1 ± 0.1	7.6 ± 0.1	8.4 ± 0.1	8.4 ± 0.1	8.3 ± 0.1	8.6 ± 0.1	8.7 ± 0.1	8.8 ± 0.1
H115A										
apo	34.9 ± 0.1	35.6 ± 0.1	36.8 ± 0.1	37.3 ± 0.1	37.9 ± 0.1	38.1 ± 0.1	37.9 ± 0.1	37.9 ± 0.1	38.1 ± 0.1	38.0 ± 0.1
TBBt	12.3 ± 0.1	11.5 ± 0.1	9.2 ± 0.1	8.9 ± 0.1	9.0 ± 0.1	8.7 ± 0.1	8.7 ± 0.1	8.7 ± 0.1	8.6 ± 0.1	8.7 ± 0.1
5,6-Br_2_Bt	6.6 ± 0.1	6.6 ± 0.1	6.6 ± 0.1	6.8 ± 0.1	7.0 ± 0.1	6.8 ± 0.1	7.1 ± 0.1	7.0 ± 0.1	7.1 ± 0.1	7.2 ± 0.1
H115F										
apo	43.1 ± 0.1	43.6 ± 0.1	44.6 ± 0.1	46.1 ± 0.1	48.3 ± 0.1	47.1 ± 0.1	48.1 ± 0.1	47.6 ± 0.1	47.2 ± 0.1	47.3 ± 0.1
TBBt	8.6 ± 0.1	8.4 ± 0.1	8.5 ± 0.1	8.0 ± 0.1	7.6 ± 0.1	7.9 ± 0.1	7.5 ± 0.1	7.7 ± 0.1	7.3 ± 0.1	7.8 ± 0.1
5,6-Br_2_Bt	5.6 ± 0.1	5.4 ± 0.1	6.0 ± 0.1	5.9 ± 0.1	6.1 ± 0.1	6.3 ± 0.1	5.8 ± 0.1	6.4 ± 0.1	6.5 ± 0.1	6.1 ± 0.1

^c^data from Ref.^18^.

As expected, for complexes formed with H160A and H160F, the ligand-induced changes in ΔT_m_ remain pH-independent for TBBt at the significance level of 0.05. Contrary to the latter, for H115A complexes with TBBt, a similar trend as in WT is preserved. For H115A, the changes in ΔT_m_ in the presence of TBBt at low pH are even more significant, reaching 12 °C and pH-dependence of ΔT_m_ in the presence of 5,6-Br_2_Bt also minutely differs from WT. Such a discrepancy results putatively from temperature-dependent effects since the H115A variant displays a significantly lower mid-point temperature of the thermal denaturation than the WT protein. At the same time, no such differences are observed for the H115F variant.

We further applied MST to analyze quantitatively the pH dependence of 5,6-Br_2_Bt and TBBt binding affinities identified in the nanoDSF. Such an approach enables precise determination of K_diss_ values for protein–ligand systems not affected by the slight heat effect associated with TBBt—hCK2α binding that precluded the application of ITC. Results for 5,6-Br_2_Bt binding to WT protein agree with those determined previously with ITC^18^. The pH-dependent change of TBBt binding affinity is confirmed for WT protein with MST, while no such effect is observed for the H160A variant. Based on these observations, the pH-dependent change of the ligand-binding affinity is most likely associated with the binding-induced histidine protonation that can be described according to Eq. (2)^44^

2 $${K}_{diss}={K}_{intd}\cdot \frac{1+{10}^{{(pK}_{a}^{f}-pH)}}{1+{10}^{{(pK}_{a}^{c}-pH)}}$$

where K_intd_ is the intrinsic dissociation constant for the neutral His160, pK_a_^f^, and pK_a_^c^ are pK_a_ values for His160 protonation in the *apo* protein and complex with TBBt, respectively. The same equation may be applied (as reported previously^18^) to describe the binding-induced 5,6-Br_2_Bt deprotonation where pK_a_ values refer to the ligand properties, and K_intd_ is the intrinsic dissociation constant for the deprotonated ligand. The intrinsic dissociation constant for the cationic form of His160 in the presence of the neutral ligand (K_intp_) may be further calculated as ${K}_{intp}={K}_{intd}\cdot {10}^{{pK}_{a}^{c}-{pK}_{a}^{f}}$.

We tested two alternative models for each protein–ligand system, assuming either pH-dependent (according to Eq. (2)) or pH-independent affinities for the strongly-binding site. The model that assumed pH-dependent K_d1_ values (Eq. (2)) was scored better than the alternative for both 5,6-Br_2_Bt and TBBt binding to WT protein (p < 10^–8^). For 5,6-Br_2_Bt, we assumed pK_a_ values determined by us recently^18^. For TBBt binding, we used the PlayMolecule ProteinPrepare application^45^ to estimate His160 pK_a_ in the *apo* hCK2α based on the available protein structures (1JWH and 1NA7). According to these calculations, the pK_a_ of His160 in the *apo* hCK2α was estimated to be 4.7, which was used in the analysis of MST data (pK_a_^f^ in Eq. (2)). Interestingly, the estimated from the MST data value of His160 pK_a_ in the complex (6.9 ± 0.7; see Table 5) corresponds to the values estimated in silico with ProteinPrepare for the hCK2α complexes with TBBt (6TLL) and 5,6-Br_2_Bt (6TLP): 5.69 and 6.62, respectively. Analogous analysis performed for CK2-TBBz complex (2OXY) confirms that the pK_a_ of His160 is unaffected by the binding of that neutral ligand.

**Table 5:** pH-dependence of ligand binding by the WT CK2 and H160A mutant monitored by MST (see Eq. (2)).

WT								
pH	TBBt				5,6-Br_2_Bt			
	Kd_1_[nM]	ΔG_1_[kJ∙mol^−1^]	Kd_2_[nM]	ΔG_2_[kJ∙mol^−1^]	Kd_1_[nM]	ΔG_1_[kJ∙mol^−1^]	Kd_2_[nM]	ΔG_2_[kJ∙mol^−1^]
6.5	5 ± 9	− 47.4 ± 4.5	320 ± 190	− 37.1 ± 1.5	240 ± 110	− 37.8 ± 1.1	1150 ± 560	− 33.9 ± 1.2
6.7	3 ± 6	− 48.6 ± 5.0	158 ± 83	− 38.8 ± 1.3	74 ± 53	− 40.7 ± 1.8	710 ± 390	− 35.1 ± 1.4
7.0	18 ± 24	− 44.2 ± 3.3	340 ± 170	− 36.9 ± 1.2	45 ± 38	− 41.9 ± 2.1	600 ± 370	− 35.5 ± 1.5
7.5	11 + 13	− 45.4 ± 2.9	380 ± 190	− 36.6 ± 1.2	26 ± 21	− 43.3 ± 2.0	670 ± 280	− 35.2 ± 1.0
8.0	16 ± 18^c^	− 44.5 ± 2.8	130 ± 88^c^	− 39.3 ± 1.7	29 ± 13^c^	− 43.0 ± 1.1	327 ± 95^c^	− 37.0 ± 0.7
Global analysis								
K_intp_*	0.2 ± 0.3	− 55.3 ± 3.7	217 ± 97	− 38.0 ± 1.1	760 ± 340	− 34.9 ± 1.1	4600 ± 1600	− 30.4 ± 0.9
K_intd_*	30 ± 19	− 42.9 ± 1.6	217 ± 97	− 38.0 ± 1.1	38 ± 17	− 42.3 ± 1.1	232 ± 78	− 37.9 ± 0.8
pK^f^; pK^c^	4.7^a^; 6.9 ± 0.6				6.9;5.6^b^			

H160A								
	TBBt				5,6-Br_2_Bt			
	Kd_1_[nM]	ΔG_1_[kJ∙mol^−1^]	Kd_2_[nM]	ΔG_2_[kJ∙mol^−1^]	Kd_1_[nM]	ΔG_1_[kJ∙mol^−1^]	Kd_2_[nM]	ΔG_2_[kJ∙mol^−1^]
6.5	15 ± 18	− 44.6 ± 3.0	290 ± 200	− 37.3 ± 1.7	1110 ± 260	− 34.0 ± 0.6	> 10,000	> − 28.5
6.7	26 ± 21	− 43.3 ± 2.0	540 ± 370	− 35.8 ± 1.7	560 ± 130	− 35.7 ± 0.6	> 10,000	> − 28.5
7.0	15 ± 18	− 44.6 ± 3.0	690 ± 470	− 35.1 ± 1.7	51 ± 49	− 41.6 ± 2.4	710 ± 490	− 35.1 ± 1.7
7.5	18 ± 16	− 44.2 ± 2.2	230 ± 120	− 37.9 ± 1.3	31 ± 37	− 42.8 ± 3.0	360 ± 290	− 36.8 ± 2.0
8.0	14 ± 10	− 44.8 ± 1.8	440 ± 210	− 36.3 ± 1.2	45 ± 33	− 41.9 ± 1.8	410 ± 290	− 36.4 ± 1.8
Global analysis								
K_int_ [nM]	17 ± 7	− 44.3 ± 1.0	400 ± 100	− 36.5 ± 0.6				
pK^f^; pK^c^	ΔpK_a_ = 0							

^a^Value calculated using PlayMolecule ProteinPrepare application^45^ based on the 1JWH and 1NA7 structures.

^b^pK values reported previously^18^.

*K_intp_ and K_intd_ refer to protonation state: protonated (cation for His160 and neutral for 5,6Br_2_Bt) and deprotonated (neutral for His160 and anion for 5,6Br_2_Bt), respectively.

In contrast to WT protein, TBBt binding by H160A was found pH-independent, while the alternative hypothesis (i.e., pH-dependent K_diss_) has to be rejected at the significance level of 0.05. Consistently, the binding of the titratable 5,6-Br_2_Bt by the H160A varies with pH (Table 5); however, the applied model assuming binding-induced shift of ligand protonation equilibrium does not satisfactorily describe the observed effect. The disagreement between the model and experimental data indicates that the binding geometry for neutral and anionic forms of 5,6-Br_2_Bt to the H160A variant of hCK2α may differ substantially.

## Discussion

Recent studies demonstrated that besides shape and hydrophobicity, the affinity of halogenated benzimidazoles and benzotriazoles to hCK2α (and similarly to other EPKs) is mainly driven by a balance of halogen/hydrogen bonding with the hinge region and hydrogen bonding/salt bridge formation with Lys68^18,40,46^. However, our recent studies^18^ pointed out that other factors might also contribute to the binding at the ATP-binding site of hCK2α, turning attention to the proximal histidine residues.

According to the available structures of hCK2α complexes, the His115 side chain is not directly involved in any interactions with ligands bound at the ATP-binding site. However, it is located just between the two residues that may form halogen/hydrogen bonds with the ligand (Glu114 and Val116 in hCK2α). Nevertheless, substituting His115 with alanine or phenylalanine affects the binding affinities of ligands interacting with the hinge region (TBBt and TBBz among the tested ones). Inspection of the hCK2α structures shows that His115 is partially solvent exposed but interacts with proximal residues (Glu114 and Val116) to place the hinge in the desired conformation. The conformation of the hinge region may directly interfere with ligand binding as it is for human and maize ATP-binding sites of CK2. In maize protein, the hinge displays solely a single, so-called open conformation, while in human protein, both open and closed conformations exist^47^. Since the only residue difference in the hinge region is His115 (human) vs. Tyr115 (maize), this residue may be directly involved in placing the hinge in open/closed conformations. The decrease of ligand-binding affinities is mutual for H115A and H115F variants. In maize CK2, there is tyrosine at this position, the direct electrostatic or hydrogen-bonding interactions involving histidine imidazole ring, rather than those with an aromatic system, seem crucial for the hinge positioning. Change in hinge conformation for the H115A variant explains the observed variations in enthalpy-entropy balance.

His160, the second of the studied histidine residues, was even more noteworthy. It may interact with numerous neighboring residues, including electrostatic interactions with Arg47, Asp120, Lys122, and Lys158, and π-π interactions formed with proximal Phe121and Asn161, and less possibly with Phe197 (Supp Fig. S46). Depending on the protonation state of His160, the thermodynamic contribution of these interactions may differ. However, the protonation state of histidine primarily affects the binding of charged ligands. Its compulsory protonation at neutral and basic conditions decreases the apparent binding affinity towards anionic ligands. We previously showed that for WT hCK2α, the pH-dependency of apparent binding affinity of 5,6-Br_2_Bt results from binding-induced perturbation of the pK_a_ of this ligand, which in the tested pH range of 6.5–8.7 predominantly exists in the anionic form^42^. Interestingly, for H160A and H160F hCK2α variants, the decrease of 5,6-Br_2_Bt binding affinity at acidic conditions is even more significant, which is reasonable since in WT, the two opposite pH-dependent effects may compensate for each other. Thus, at low pH, the estimated difference in free energy of binding caused by histidine protonation is ~ 3 kJ∙mol^−1^ (deduced from TBBt binding by WT protein), while caused by 5,6-Br_2_Bt deprotonation is from 3 to 5 kJ∙mol^−1^ (based on 5,6-Br_2_Bt binding affinities to WT). So, at moderately acidic conditions, the unfavorable contribution of forced 5,6-Br_2_Bt deprotonation is partly compensated by accompanying histidine protonation. One can thus assume that at pH of 6.5, the net change of the free energy of binding caused by 5,6-Br_2_Bt deprotonation is up to 8 kJ∙mol^−1^, and this is indeed the difference in free energy of binding for H160A-5,6-Br_2_Bt (i.e., ΔG_1_(pH 6.5)–ΔG_1_(pH 8.0)).

However, for the H160A variant, the observed change of 5,6-Br_2_Bt binding affinity with pH cannot be explained, assuming binding-induced deprotonation of the ligand model (Eq. (3)). At pH 6.5, the maximal penalty for deprotonation of 5,6-Br_2_Bt (with pK^f^_a_ 6.93) is ~ 3 kJ∙mol^−1^, as calculated according to relation:

3 $$\Delta \Delta G=RT\cdot ln\left(\frac{1+{10}^{{(pK}_{a}^{f}-pH)}}{1+{10}^{{(pK}_{a}^{c}-pH)}}\right)$$

The binding affinity of non-dissociable analog 1-CH_3_-5,6-Br_2_Bt was previously estimated with ITC to 1.56 ± 0.52 μM (− 33.1 ± 0.8 kJ·mol^−1^)^18^. K_d_ for the binding of the neutral form of 5,6-Br_2_Bt was estimated previously to ~ 1 µM^18^, and based on results presented here, ΔG_intp_ is − 34.9 ± 1.1 kJ·mol^−1^. All these values agree with the value estimated for the H160A variant at pH 6.5 (− 34.0 ± 0.6 kJ·mol^−1^). Altogether indicates that at moderately acidic conditions, the binding of an anionic ligand is thermodynamically less favored for hCK2α variants in which a neutral residue replaces His160. It could be that this replacement makes the binding pose of 5,6-Br_2_Bt at the ATP-binding site of hCK2α pH-controllable.

To summarize, the binding of the anionic ligand by WT hCK2α is preferred not only due to hydrogen bond/salt-bridge formation with Lys68 but also by long-range electrostatic interactions with positively charged His160. While His115 residue probably is directly involved in placing the hinge in open/closed conformations in hCK2α.

## Methods

This manuscript does not involve the use of any animal or human sample/data.

### Site-directed mutagenesis of hCK2α

hCK2α amino acid-substituted mutants (H115A, H115F, H160A, and H160F) were obtained via site-directed mutagenesis using the QuikChange Lightning Site-Directed Mutagenesis Kit (Agilent Technologies, Santa Clara, USA). The DNA primers used for mutagenesis are listed in Table S1. The starting DNA template pET28-hCK2α plasmid was used^39^. Following the manufacturer’s guidelines, mutagenesis reactions were performed in a 50-μL final volume containing 1 × QuikCHange Lightning reaction buffer, 50 ng template plasmid DNA, 125 ng of forward and reverse primers, 1 μL of 10 mM dNTP mix, 1. 5 μL QuikSolution and 1 μL (2.5 U) of Pfu DNA polymerase. The PCR cycling conditions were as follows: 95 °C for 2 min, 18 cycles of 95 °C for 20 s, 65 °C for 10 s, 68 °C for 2.5 min, and additionally 5 min at 68 °C. Next, methylated plasmid DNA was digested for 5 min at 37 °C with 2 μL of DpnI restriction enzyme. 2 µl of the Dpn I-treated DNA was transformed into *E. coli* XL10-Gold cells. After transformation, the plasmid was isolated from selected colonies using GeneJET Plasmid Miniprep Kit (ThermoFisher Scientific, Waltham, USA) and analyzed by DNA sequencing for desired mutations.

### Expression and purification of WT hCK2α and mutants

The catalytic subunit of human CK2 (hCK2α) and all its variants were expressed and purified as described previously^39^. Protein sample homogeneity was routinely confirmed by gel electrophoresis.

### Ligand synthesis

*5,6-dibromo-1H-benzimidazole (5,6-Br*_*2*_*Bz)*: 4,5-drobromophenyl-1,2-diamine (5 mmol, 1.32 g) was dissolved in 3 ml of formic acid and heated overnight at 100 °C. Afterward, the reaction was quenched by adding 30 mL of water. Precipitate was filtered, washed with saturated NaHCO_3_ solution (10 mL), then distilled water (3 × 10 mL), obtained 1.37 g of product (quantitative yield); ^1^H NMR (500 MHz, d-DMSO) δ 8.01 (2H, s, Ar*H*), 8.34 (1H, s, Ar*H*). ^1^H NMR spectrum shown in Supp Fig. S1 (top).

*5,6,7-tribromo-1H-benzimidazole (4,5,6-Br*_*3*_*Bz)*: 5,6-dibromo-1*H*-benzimidazole (0.5 mmol, 138 mg) was dissolved in 1.5 mL of conc. nitric acid, and Bromine (0.5 mmol, 40 mg) was added dropwise. The reaction mixture was heated at 60 °C overnight. Afterward, the reaction was cooled to room temperature, and a saturated NaHCO_3_ solution (10 mL) was added. Precipitate was filtered, then purified by column chromatography on silica gel using hexane/AcOEt (8:2; v/v) as eluent to give 4,5,6-tribromo-1*H*-benzimidazole (95 mg); yield = 53%; ^1^H NMR (500 MHz, d-DMSO) δ 8.04 (1H, s, Ar*H*), 8.38 (1H, s, Ar*H*). ^1^H NMR spectrum shown in Supp Fig. S1 (bottom).

*4,5,6,7-tetrabromo-1H-benzimidazole (4,5,6,7-Br*_*4*_*Bz, TBBz)*: synthesized according to the previously reported procedure^48^.

### UV-monitored titration

pK_a_ values of the newly synthesized compounds were determined at 298 K by spectrophotometric titration in the pH range of 13–3.5. Measurements were carried out on Perkin Elmer Lambda 25 UV–VIS spectrometer in the 200–500 nm range with a thermostated cell holder. The spectra corrected for the scattering caused by solute aggregates were further globally analyzed according to the Henderson–Hasselbach formula:

$$\varepsilon \left(\lambda ,pH\right)=\frac{{\varepsilon }_{n}\left(\lambda \right){\cdot 10}^{pH}+{\varepsilon }_{a}\left(\lambda \right){\cdot 10}^{{pK}_{a}}}{{10}^{pH}{+10}^{{pK}_{a}}}$$

where ε (λ, pH) is the spectrum recorded at a given pH, and ε_n_(λ), ε_a_(λ) are the reference spectra for the neutral and dissociated forms.

### Circular Dichroism spectroscopy (CD)

Circular dichroism spectra were recorded on a Jasco J-815 spectropolarimeter. Each CD spectrum was measured thrice at 25 °C in the 195–270 nm range. All the spectra were recorded using a 1-mm path-length quartz cell for the ~ 2 μM protein solution in 25 mM Tris–HCl pH 8 buffer containing 0.5 M NaClO_4_. The exact protein concentration was determined for each sample based on UV absorbance at 280 nm, assuming a molar extinction coefficient ε = 61,895 M^−1^ cm^−1^.

### Activity assay

The enzymatic activity was monitored based on the luminescence measured with the SpectraMax iD3 Multi-Mode Microplate Reader (Molecular Devices, San Jose, CA, USA), with the aid of ADP-Glo kinase assay (Promega, Walldorf, Germany). The measurements were carried in a 96-well plate in a volume of 25 μl in 20 mM Tris–HCl buffer (pH 7.5), containing 100 ng of tested hCK2α variant (1 μl), 10 μM CK2 substrate peptide RRRDDDSDDD (Biaffin GmbH & Co KG), 10 μM ATP and 20 mM MgCl_2_. The reaction was initiated by adding the enzyme and continued for 20 min at 30 °C. For each protein variant, at least three independent experiments were performed. The activity was calculated as average with standard deviation and presented as a percent of WT protein activity.

### Low-volume differential scanning fluorimetry (nanoDSF)

All experiments were carried out with constant protein and ligand concentrations of 2.5 μM and 25 μM, respectively. The effect of ligand was measured in 25 mM Tris–HCl pH 8, 0.5 NaCl buffer, and pH-dependent binding was monitored for a pH range of 6.5–8.7 in 25 mM Bis–Tris Propane, 0.5 M NaCl buffer. The samples were loaded into nanoDSF Grade Standard Capillaries (NanoTemper Technologies) and analyzed using the Prometheus NT.48 nanoDSF device (NanoTemper Technologies). Detailed experimental setup and data analysis procedure was described previously^41^.

### Microscale thermophoresis (MST)

Experiments were carried out in two different buffers depending on the pH (MES for pH < 7 and HEPES for pH ≥ 7) with 0.5 M NaCl. The protein sample was initially labeled with the His-Tag Labeling Kit RED-tris-NTA 2nd Generation. In agreement with the manufacturer’s guidelines, 16 serial two-fold dilutions of the ligand were tested in each MST experiment. The concentration of labeled protein was kept constant at 50 nM. The ligand-to-protein concentration ratio was tested up to 25 µM ligand. Samples were loaded into Monolith™ NT.115 Premium Capillaries (NanoTemper). After a short incubation period, MST analysis was performed using the Monolith™ NT.115 (NanoTemper Technologies). A numerical model of two independent binding sites described before^19^ was globally fitted to at least 3 MST pseudo-titration experiments (for each ligand at each pH). All the pseudo-titration data are shown in Supplementary Figs. S2–S27.

### Isothermal titration calorimetry (ITC)

ITC measurements were carried out using MicroCal iTC200 (Malvern). The protein samples were transferred to the appropriate buffer using Pierce™ Polyacrylamide Spin Desalting Columns (Thermo Scientific). Stock ligand DMSO solutions were diluted with the appropriate DMSO volume before mixing with the buffer to obtain the required ligand concentration with a final DMSO content of 1%. For each ligand, at least two independent titration experiments were done. Details of the experimental setup and the analysis algorithm were previously described^49^. For binding of TBBt and 4,5,6-Br_3_Bt by WT and H160A variant, the model of two independent binding sites was applied. However, contrary to our previous approach^49^, due to the low heat of binding, the correction for enthalpy of ligand mixing with buffer was constrained to the value determined from the single-binding site model applied to the same system. All the titration data are shown in Supplementary Figs. S28–S45.

### Numeric analysis of presented data

All appropriate models were fitted to the experimental data using the Levenberg–Marquardt algorithm implemented in the Origin package (ver. 9.9, www.originlab.com). The standard errors for the derived parameters were estimated according to the Error Propagation formula (https://www.originlab.com/doc/origin-help/nlfit-theory).

### Supplementary Information

Supplementary Information.

## Data Availability

All raw datasets generated within this study are available from the corresponding author on a request.

## References

[1] Martínez A (1995). Evidence for a functionally important histidine residue in human tyrosine hydroxylase. Amino Acids.

[2] Noh H (2020). Histidine residues at the copper-binding site in human tyrosinase are essential for its catalytic activities. J. Enzyme Inhib. Med. Chem..

[3] Gasparini S (1991). Identification of structurally and functionally important histidine residues in cytoplasmic aspartyl-tRNA synthetase from Saccharomyces cerevisiae. Biochemistry.

[4] Bhattacharyya DK, Kwon O, Meganathan R (1997). Vitamin K2 (menaquinone) biosynthesis in Escherichia coli: Evidence for the presence of an essential histidine residue in o-succinylbenzoyl coenzyme A synthetase. J. Bacteriol..

[5] Rötzschke O, Lau JM, Hofstätter M, Falk K, Strominger JL (2002). A pH-sensitive histidine residue as control element for ligand release from HLA-DR molecules. Proc. Natl. Acad. Sci..

[6] Zhang L (2015). Functional role of histidine in the conserved his-x-asp motif in the catalytic core of protein kinases. Sci. Rep..

[7] Malinska M, Dauter M, Kowiel M, Jaskolski M, Dauter Z (2015). Protonation and geometry of histidine rings. Acta Crystallogr. Sect. D.

[8] Liao S-M, Du Q-S, Meng J-Z, Pang Z-W, Huang R-B (2013). The multiple roles of histidine in protein interactions. Chem. Central J..

[9] Hunter CA, Lawson KR, Perkins J, Urch CJ (2001). Aromatic interactions. J. Chem. Soc. Perkin Trans..

[10] Reddy AS, Sastry GN (2005). Cation [M = H+, Li+, Na+, K+, Ca2+, Mg2+, NH4+, and NMe4+] interactions with the aromatic motifs of naturally occurring amino acids: A theoretical study. J. Phys. Chem. A.

[11] Burley SK, Petsko GA (1986). Amino-aromatic interactions in proteins. FEBS Lett..

[12] Matsumura H (2008). Novel cation-π interaction revealed by crystal structure of thermoalkalophilic lipase. Proteins Struct. Funct. Bioinform..

[13] Rutledge LR, Churchill CDM, Wetmore SD (2010). A preliminary investigation of the additivity of π−π or π+−π stacking and T-shaped Interactions between natural or damaged DNA nucleobases and histidine. J. Phys. Chem. B.

[14] Bhattacharyya R, Saha RP, Samanta U, Chakrabarti P (2003). Geometry of interaction of the histidine ring with other planar and basic residues. J. Proteome Res..

[15] Churchill CDM, Wetmore SD (2009). Noncovalent interactions involving histidine: The effect of charge on π−π stacking and T-shaped interactions with the DNA nucleobases. J. Phys. Chem. B.

[16] Perutz MF (1993). The role of aromatic rings as hydrogen-bond acceptors in molecular recognition. Philos. Trans. Phys. Sci. Eng..

[17] Chakrabarti P (1990). Geometry of interaction of metal ions with histidine residues in protein structures. Protein Eng. Design Select..

[18] Winiewska-Szajewska M (2022). Competition between electrostatic interactions and halogen bonding in the protein–ligand system: Structural and thermodynamic studies of 5,6-dibromobenzotriazole-hCK2α complexes. Sci. Rep..

[19] Winiewska M, Bugajska E, Poznański J (2017). ITC-derived binding affinity may be biased due to titrant (nano)-aggregation. Binding of halogenated benzotriazoles to the catalytic domain of human protein kinase CK2. PLOS ONE.

[20] Winiewska M (2015). Thermodynamic parameters for binding of some halogenated inhibitors of human protein kinase CK2. Biochem. Biophys. Res. Commun..

[21] Pinna LA (2012). Protein Kinase CK2.

[22] Okochi M (2000). Constitutive phosphorylation of the Parkinson's disease associated alpha-synuclein. J. Biol. Chem..

[23] Ishii A (2007). Casein kinase 2 is the major enzyme in brain that phosphorylates Ser129 of human alpha-synuclein: Implication for alpha-synucleinopathies. FEBS Lett..

[24] Iimoto DS, Masliah E, DeTeresa R, Terry RD, Saitoh T (1990). Aberrant casein kinase II in Alzheimer's disease. Brain Res..

[25] Masliah E (1992). Casein kinase II alteration precedes tau accumulation in tangle formation. Am. J. Pathol..

[26] Martin L, Latypova X, Terro F (2011). Post-translational modifications of tau protein: Implications for Alzheimer's disease. Neurochem. Int..

[27] Hubert A (2006). Casein kinase 2 inhibition decreases hypoxia-inducible factor-1 activity under hypoxia through elevated p53 protein level. J. Cell Sci..

[28] Mottet D, Ruys SP, Demazy C, Raes M, Michiels C (2005). Role for casein kinase 2 in the regulation of HIF-1 activity. Int. J. Cancer.

[29] Harvey EJ, Li N, Ramji DP (2007). Critical role for casein kinase 2 and phosphoinositide-3-kinase in the interferon-gamma-induced expression of monocyte chemoattractant protein-1 and other key genes implicated in atherosclerosis. Arterioscler. Thromb. Vasc. Biol..

[30] Guerra B, Issinger OG (2008). Protein kinase CK2 in human diseases. Curr. Med. Chem..

[31] Borgo C, D’Amore C, Sarno S, Salvi M, Ruzzene M (2021). Protein kinase CK2: a potential therapeutic target for diverse human diseases. Signal Transduct. Target. Ther..

[32] Seldin DC, Landesman-Bollag E, Pinna LA (2013). The oncogenic potential of CK2. Protein Kinase CK2.

[33] Gordon DE (2020). A SARS-CoV-2 protein interaction map reveals targets for drug repurposing. Nature.

[34] Bouhaddou M (2020). The global phosphorylation landscape of SARS-CoV-2 infection. Cell.

[35] Pagano MA (2004). 2-Dimethylamino-4,5,6,7-tetrabromo-1H-benzimidazole: A novel powerful and selective inhibitor of protein kinase CK2. Biochem. Biophys. Res. Commun..

[36] Szyszka R, Grankowski N, Felczak K, Shugar D (1995). Halogenated benzimidazoles and benzotriazoles as selective inhibitors of protein kinases CK I and CK II from Saccharomyces cerevisiae and other sources. Biochem. Biophys. Res. Commun..

[37] Meggio F, Shugar D, Pinna LA (1990). Ribofuranosyl-benzimidazole derivatives as inhibitors of casein kinase-2 and casein kinase-1. Eur. J. Biochem..

[38] Łukowska-Chojnacka E, Wińska P, Wielechowska M, Poprzeczko M, Bretner M (2016). Synthesis of novel polybrominated benzimidazole derivatives—Potential CK2 inhibitors with anticancer and proapoptotic activity. Bioorg. Med. Chem..

[39] Winiewska M, Kucińska K, Makowska M, Poznański J, Shugar D (2015). Thermodynamics parameters for binding of halogenated benzotriazole inhibitors of human protein kinase CK2α. Biochim. Biophys. Acta.

[40] Battistutta R (2007). The ATP-binding site of protein kinase CK2 holds a positive electrostatic area and conserved water molecules. ChemBioChem.

[41] Czapinska H (2021). Halogen atoms in the protein-ligand system. Structural and thermodynamic studies of the binding of bromobenzotriazoles by the catalytic subunit of human protein kinase CK2. The Journal of Physical Chemistry B.

[42] Wasik R, Winska P, Poznanski J, Shugar D (2012). Synthesis and physico-chemical properties in aqueous medium of all possible isomeric brom analogues of benzo-1H-triazole, potential inhibitors of protein kinases. J. Phys. Chem. B.

[43] Czapinska H (2021). Halogen atoms in the protein-ligand system. Structural and thermodynamic studies of the binding of bromobenzotriazoles by the catalytic subunit of human protein kinase CK2. J. Phys. Chem. B..

[44] Baker BM, Murphy KP (1996). Evaluation of linked protonation effects in protein binding reactions using isothermal titration calorimetry. Biophys. J..

[45] Martínez-Rosell G, Giorgino T, De Fabritiis G (2017). PlayMolecule ProteinPrepare: A web application for protein preparation for molecular dynamics simulations. J. Chem. Inform. Model..

[46] Battistutta R, De Moliner E, Sarno S, Zanotti G, Pinna LA (2001). Structural features underlying selective inhibition of protein kinase CK2 by ATP site-directed tetrabromo-2-benzotriazole. Protein Sci..

[47] Raaf J, Brunstein E, Issinger OG, Niefind K (2008). The CK2 alpha/CK2 beta interface of human protein kinase CK2 harbors a binding pocket for small molecules. Chem. Biol..

[48] Zien P, Bretner M, Zastapilo K, Szyszka R, Shugar D (2003). Selectivity of 4,5,6,7-tetrabromobenzimidazole as an ATP-competitive potent inhibitor of protein kinase CK2 from various sources. Biochem. Biophys. Res. Commun..

[49] Paprocki D, Winiewska-Szajewska M, Speina E, Kucharczyk R, Poznański J (2021). 5,6-diiodo-1H-benzotriazole: New TBBt analogue that minutely affects mitochondrial activity. Sci. Rep..

