# Supplementary material for: Effect of histidine protonation state on ligand binding at the ATP-binding site of human protein kinase CK2
Source: Sci Rep. 2024 Jan 17;14:1463. doi: 10.1038/s41598-024-51905-y (PMC10794401; doi:10.1038/s41598-024-51905-y)
Supplement: Supplementary file 1 — Supplementary Information. [file 41598_2024_51905_MOESM1_ESM.docx]

**Supplementary material for:**

Maria Winiewska-Szajewska^1,2*^, Daniel Paprocki^1^, Ewa Marzec^1^, Jarosław Poznański^1*^

^1^Institute of Biochemistry and Biophysics PAS, Pawinskiego 5a, 02-106 Warsaw, Poland

^2^Division of Biophysics, Institute of Experimental Physics, University of Warsaw, Pasteura 5, 02-089 Warsaw, Poland;

*Correspondence to be addressed to:

jarek@ibb.waw.pl; mwin@ibb.waw.pl

**Supplementary Tables**

**Table S1.** DNA primers used in mutagenesis

| **Introduced mutation** | **Designed primers for site-directed mutagenesis** |
| --- | --- |
| H115A (CAC → GCC) | Forward: 5' ACCCCCGCCTTGGTTTTTGAAGCCGTAAACAACACAGAC 3'  Reverse: 5' GTCTGTGTTGTTTACGGCTTCAAAAACCAAGGCGGGGGT 3' |
| H115F (CAC → TTC) | Forward: 5' AACCCCCGCCTTGGTTTTTGAATTCGTAAACAACACAGACT 3'  Reverse: 5' AGTCTGTGTTGTTTACGAATTCAAAAACCAAGGCGGGGGTT 3' |
| H160A (CAT → GCT) | Forward: 5' TGCACAGAGATGTCAAGCCCGCTAATGTCATGATTGATCATG 3'  Reverse: 5' CATGATCAATCATGACATTAGCGGGCTTGACATCTCTGTGCA 3' |
| H160F (CAT → TTT) | Forward: 5’AGAGATGTCAAGCCCTTCAATGTCATG 3’  Reverse: 5’CATGACATTGAAGGGCTTGACATCTCT 3’ |

**Table S2.** Comparison of two fitting method

|  | | | |  | | |
| --- | --- | --- | --- | --- | --- | --- |
|  | **Parameter estimated using one binding site model** | | | **Parameter estimated using two binding sites model** | | |
|  | **TBBt+WT** | **4,5,6-Br_3_Bt+WT** | **TBBt+H160A** | **TBBt+WT** | **4,5,6-Br_3_Bt+WT** | **TBBT+H160A** |
| Kd_1_[nM] | 293±58 | 113±15 | 373±235 | 24±8 | 29±45 | 3±2 |
| ΔG_1_[kJ∙mol^-1^] | -37.3±0.5 | -39.6±0.3 | -36.7±1.6 | -43.5±0.8 | -43.1±3.9 | -48.4±1.5 |
| ΔH_1_[kJ∙mol^-1^] | -31±1 | -50±1 | -26±3 | -20±3 | -34±5 | -21±1 |
| ΔS_1_[J∙mol^-1^∙K^-1^] | 22±5 | -36±4 | 36±15 | 78±13 | 30±29 | 93±6 |
| Kd_2_[nM] | - | | | 82±33 | 161±72 | 169±66 |
| ΔG_2_[kJ∙mol^-1^] |  |  |  | -40.4±1.0 | -38.8±1.1 | -38.7±1.0 |
| ΔH_2_[kJ∙mol^-1^] |  |  |  | -5±3 | -29±4 | -8±1 |
| ΔS_2_[J∙mol^-1^∙K^-1^] |  |  |  | 120±14 | 33±18 | 102±5 |
|  | Fitting statistics | | | | | |
| R-square | 0.9896 | 0.99668 | 0.89654 | 0.99369 | 0.99675 | 0.98944 |
| RSS | 2.60004 | 0.30441 | 13.87023 | 1.57777 | 0.29849 | 1.29258 |
| Bayesian Information Criterion (BIC) | -90.68628 | -106.61071 | -1.5053 | -115.40349 | -110.49346 | -58.98755 |
| F-test at a significance level 0.05 | worse | worse | worse | better | better | better |


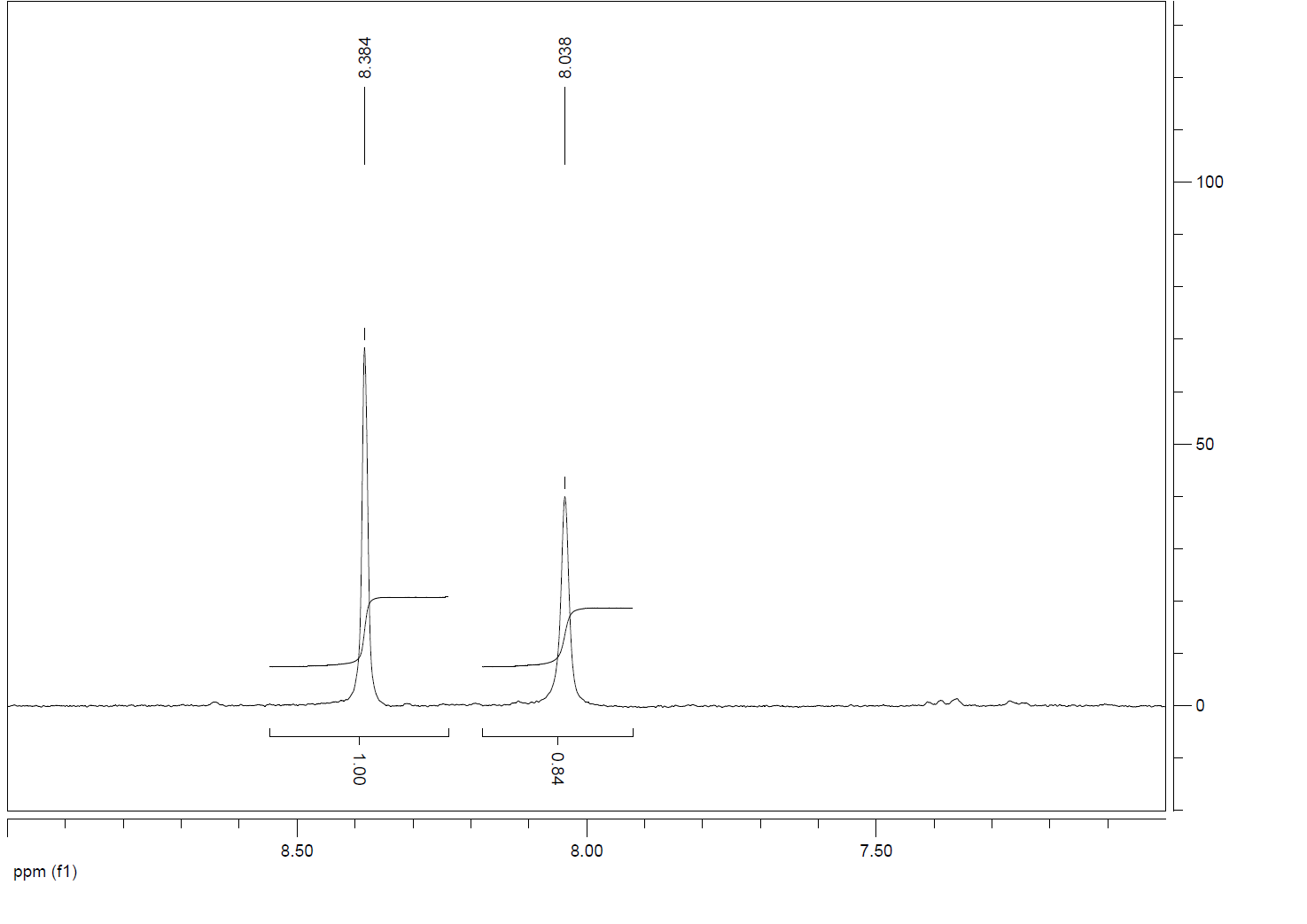


**Figure S1.** Aromatic region of ^1^H NMR spectra of 5,6-Br_2_Bz (top) and 4,5,6-Br_3_Bz (down). The not shown remaining signals are assigned to the solvent.

**
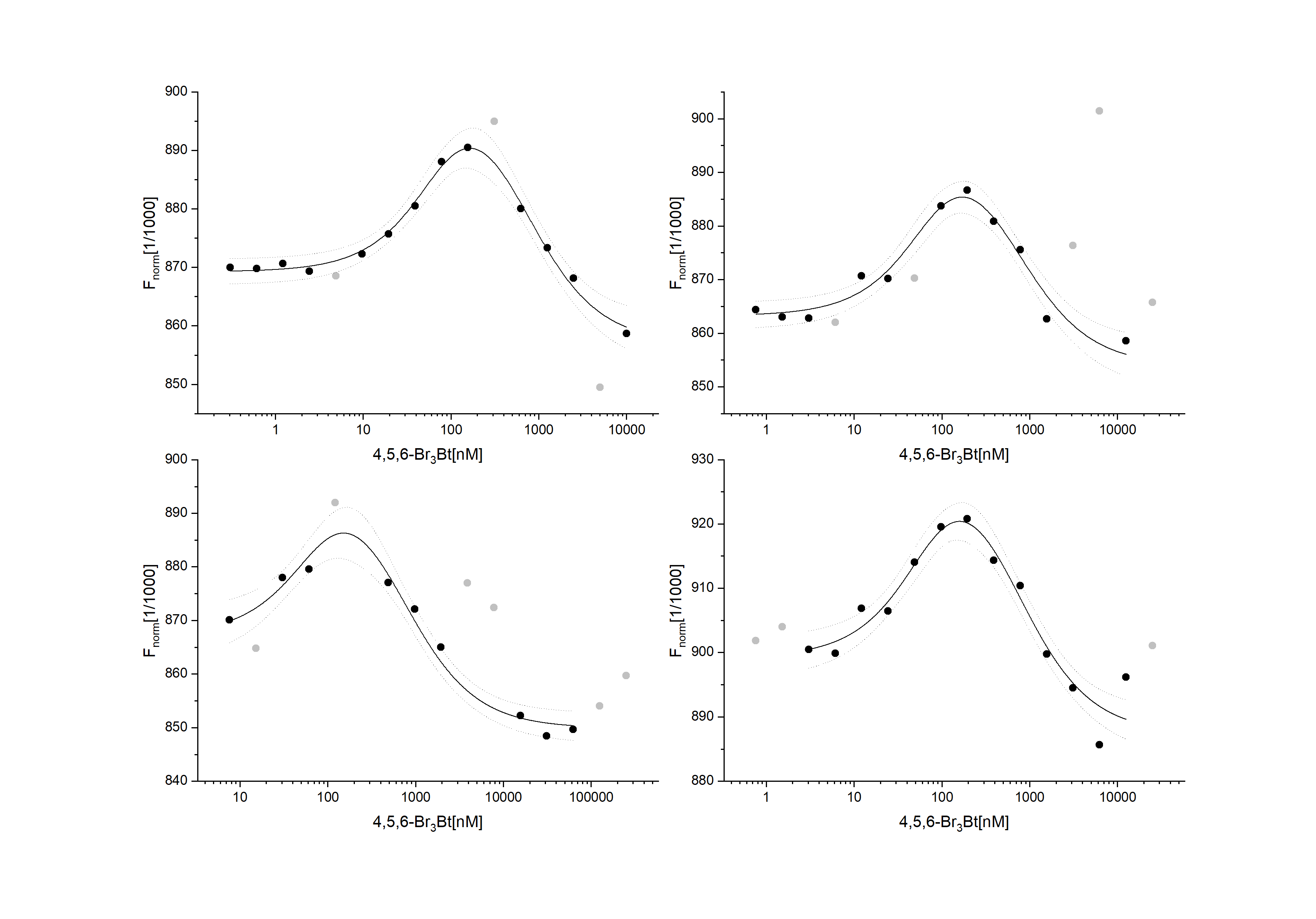
**

**Figure S2.** MST pseudo-titration data collected for **WT hCK2α and 4,5,6-Br_3_Bt**. Black circles shows experimental data, grey ones indicate data removed from the analysis, thick line represents the fitted model of two independent biding sites and dotted ones boarder the 95% confidence limits for the model.

**
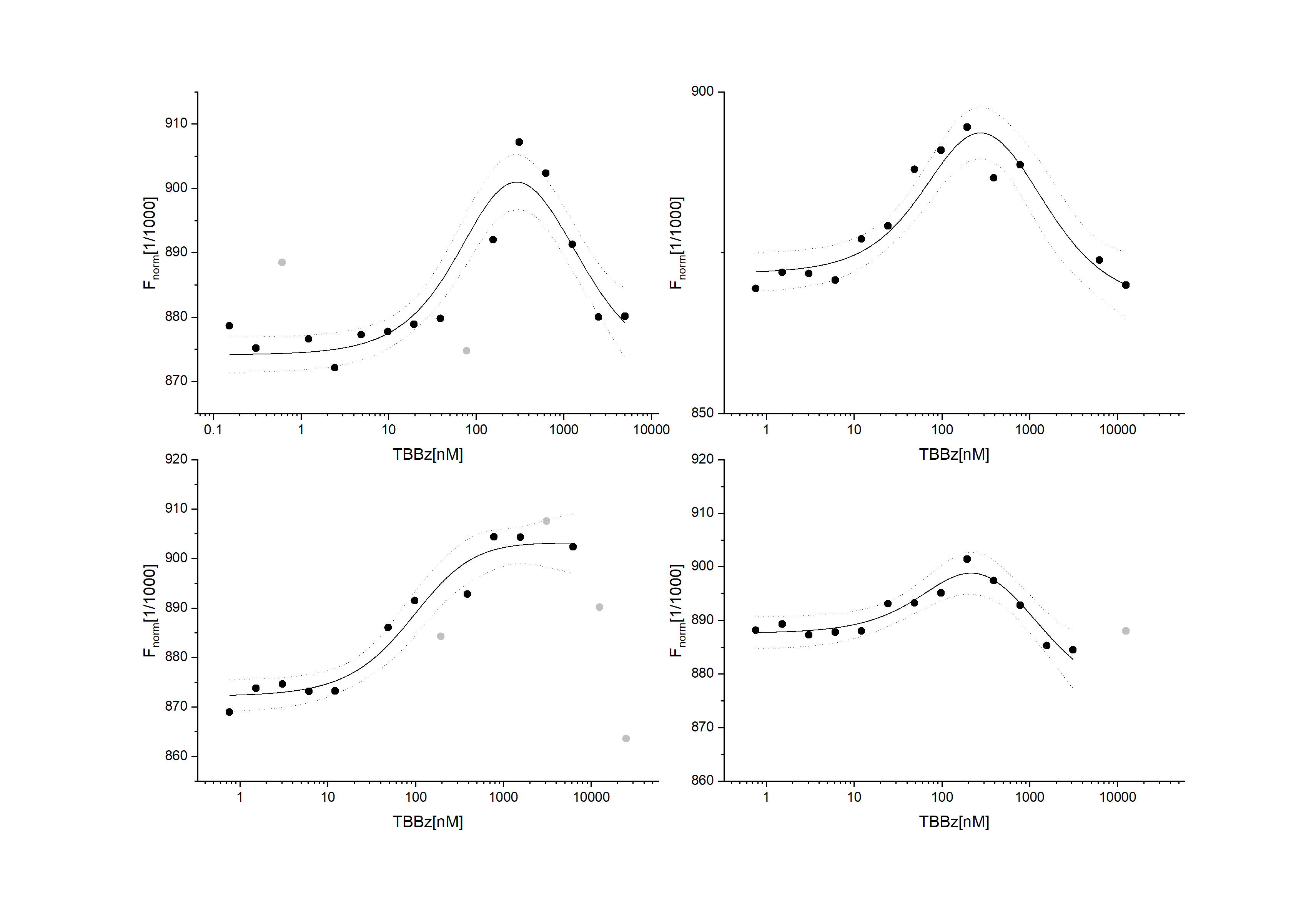
**

**Figure S3.** MST pseudo-titration data collected for **WT hCK2α and TBBz**. Black circles shows experimental data, grey ones indicate data removed from the analysis, thick line represents the fitted model of two independent biding sites and dotted ones boarder the 95% confidence limits for the model.


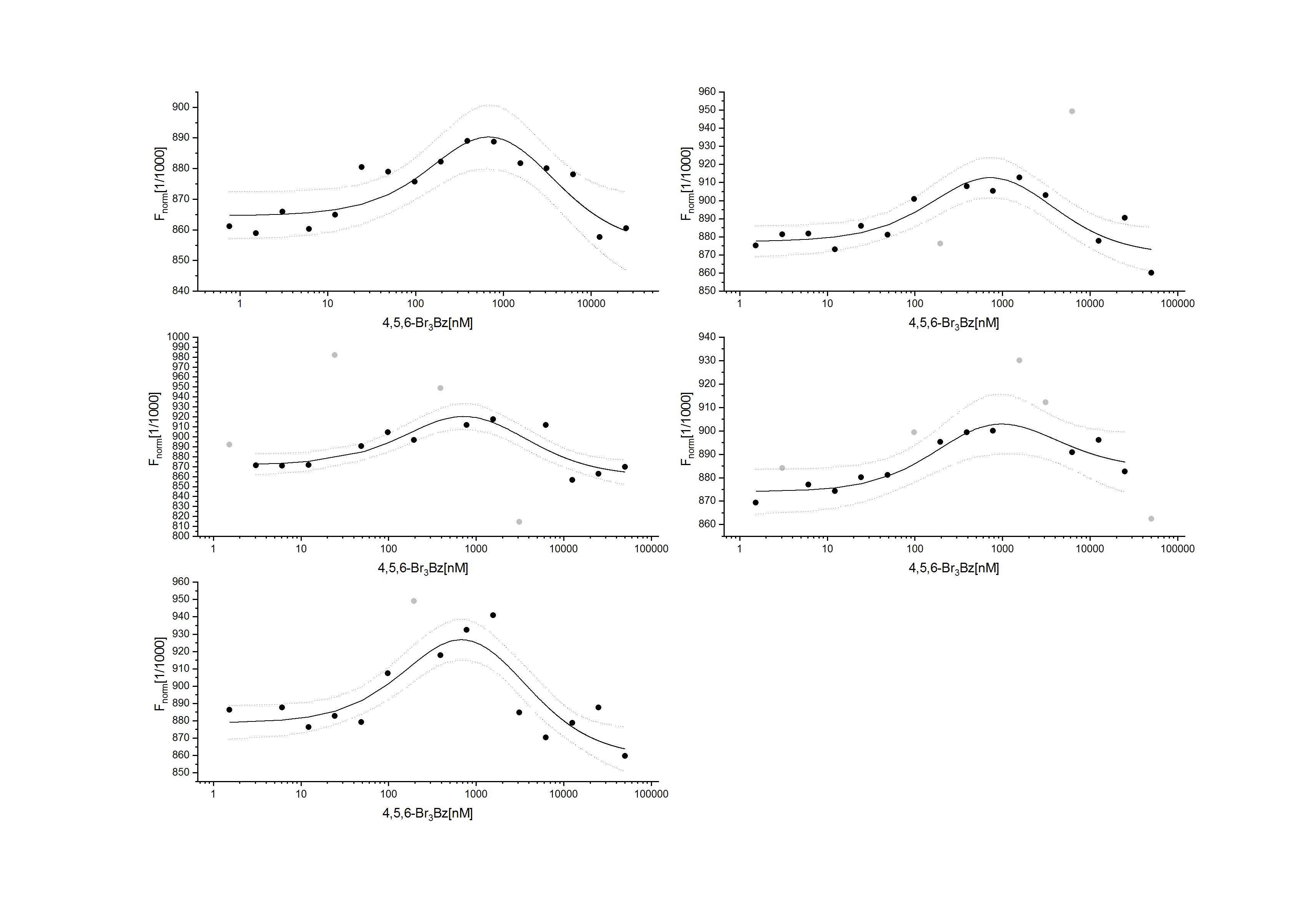


**Figure S4.** MST pseudo-titration data collected for **WT hCK2α and 4,5,6-Br_3_Bz**. Black circles shows experimental data, grey ones indicate data removed from the analysis, thick line represents the fitted model of two independent biding sites and dotted ones boarder the 95% confidence limits for the model.

**
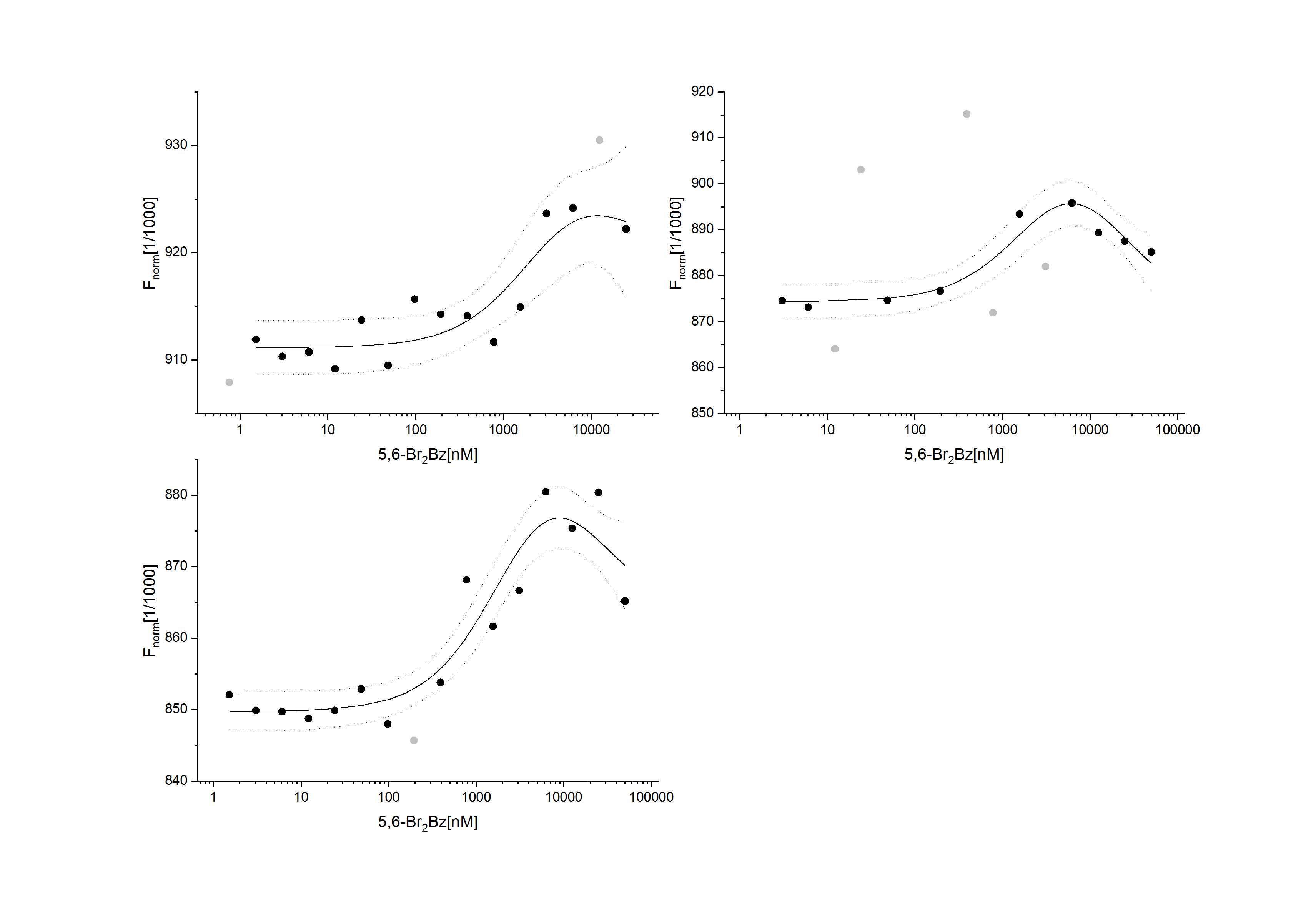
**

**Figure S5.** MST pseudo-titration data collected for **WT hCK2α and 5,6-Br_2_Bz**. Black circles shows experimental data, grey ones indicate data removed from the analysis, thick line represents the fitted model of two independent biding sites and dotted ones boarder the 95% confidence limits for the model.

**
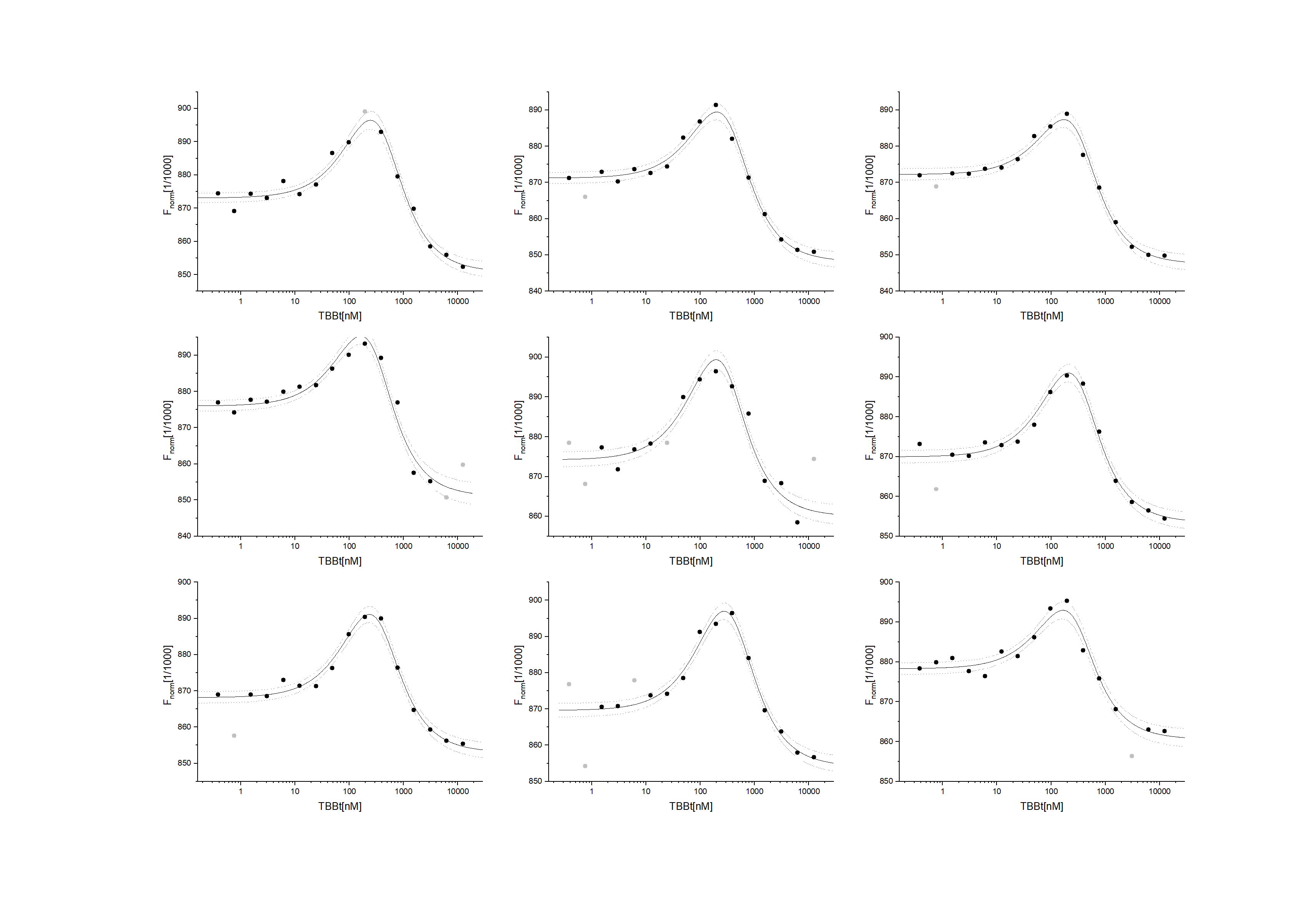
**

**Figure S6.** MST pseudo-titration data collected for **H115F hCK2α and TBBt**. Black circles shows experimental data, grey ones indicate data removed from the analysis, thick line represents the fitted model of two independent biding sites and dotted ones boarder the 95% confidence limits for the model.

**
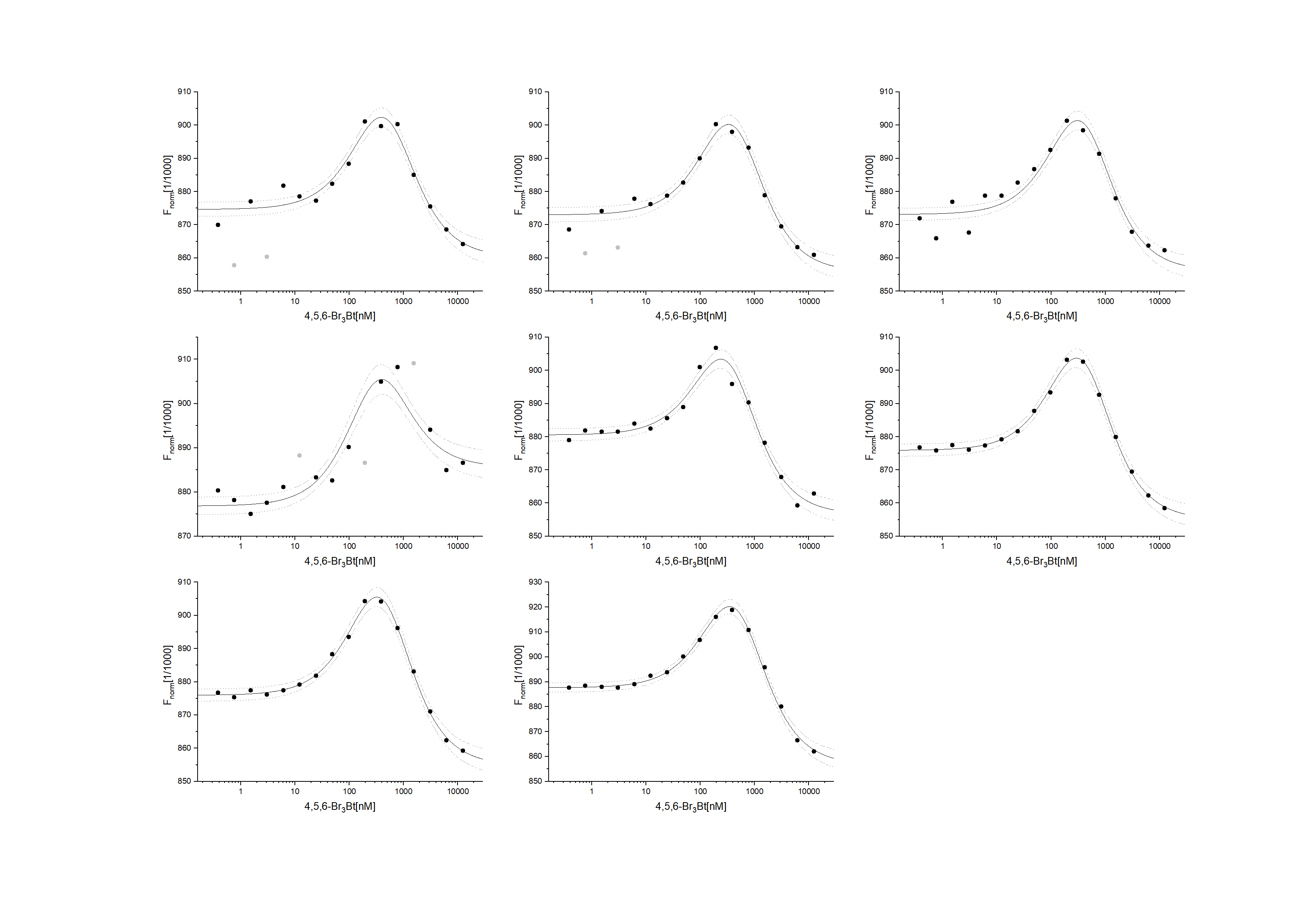
**

**Figure S7.** MST pseudo-titration data collected for **H115F hCK2α and 4,5,6-Br_3_Bt**. Black circles shows experimental data, grey ones indicate data removed from the analysis, thick line represents the fitted model of two independent biding sites and dotted ones boarder the 95% confidence limits for the model.

**
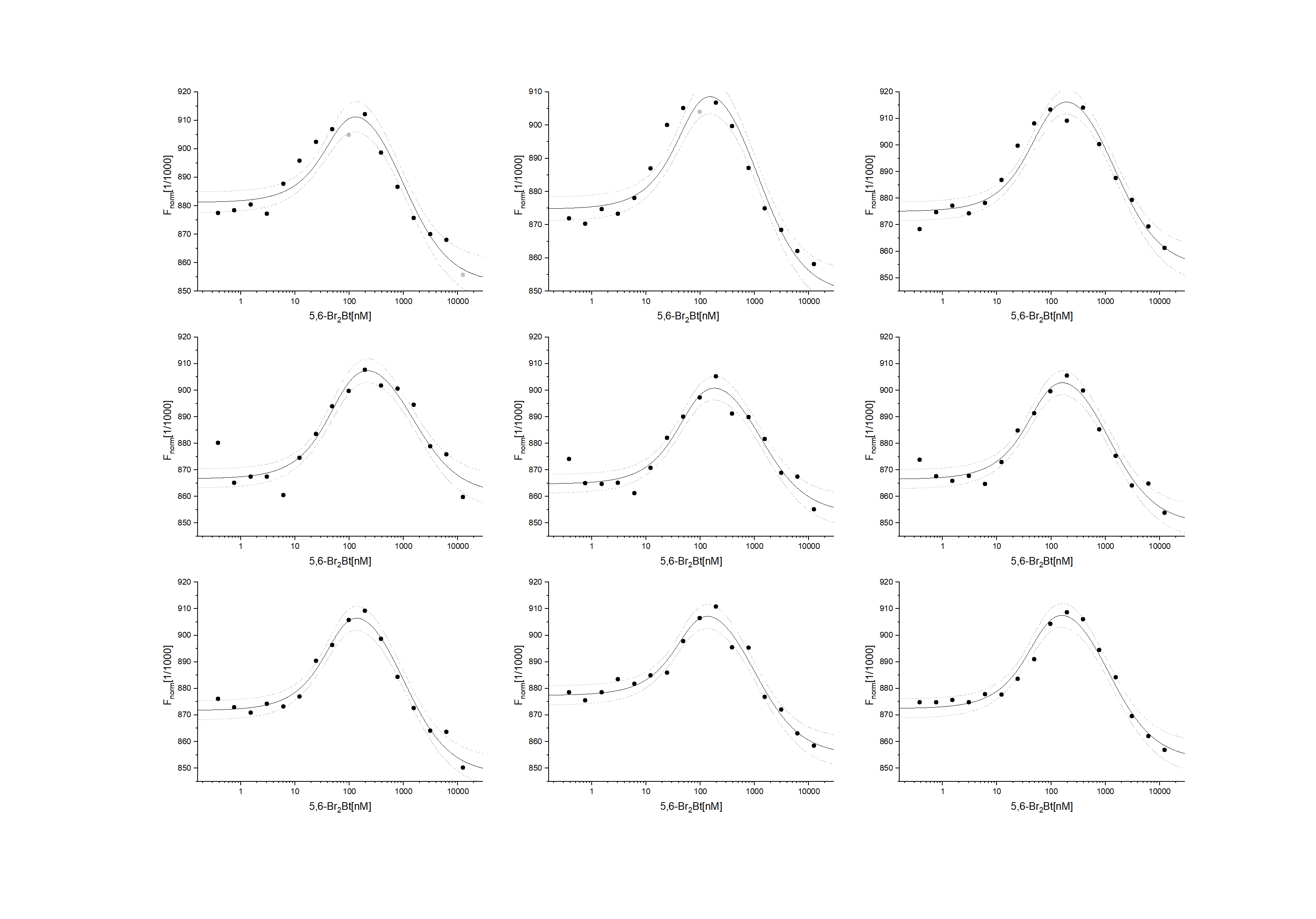
**

**Figure S8.** MST pseudo-titration data collected for **H115F hCK2α and 5,6-Br_2_Bt**. Black circles shows experimental data, grey ones indicate data removed from the analysis, thick line represents the fitted model of two independent biding sites and dotted ones boarder the 95% confidence limits for the model.

**
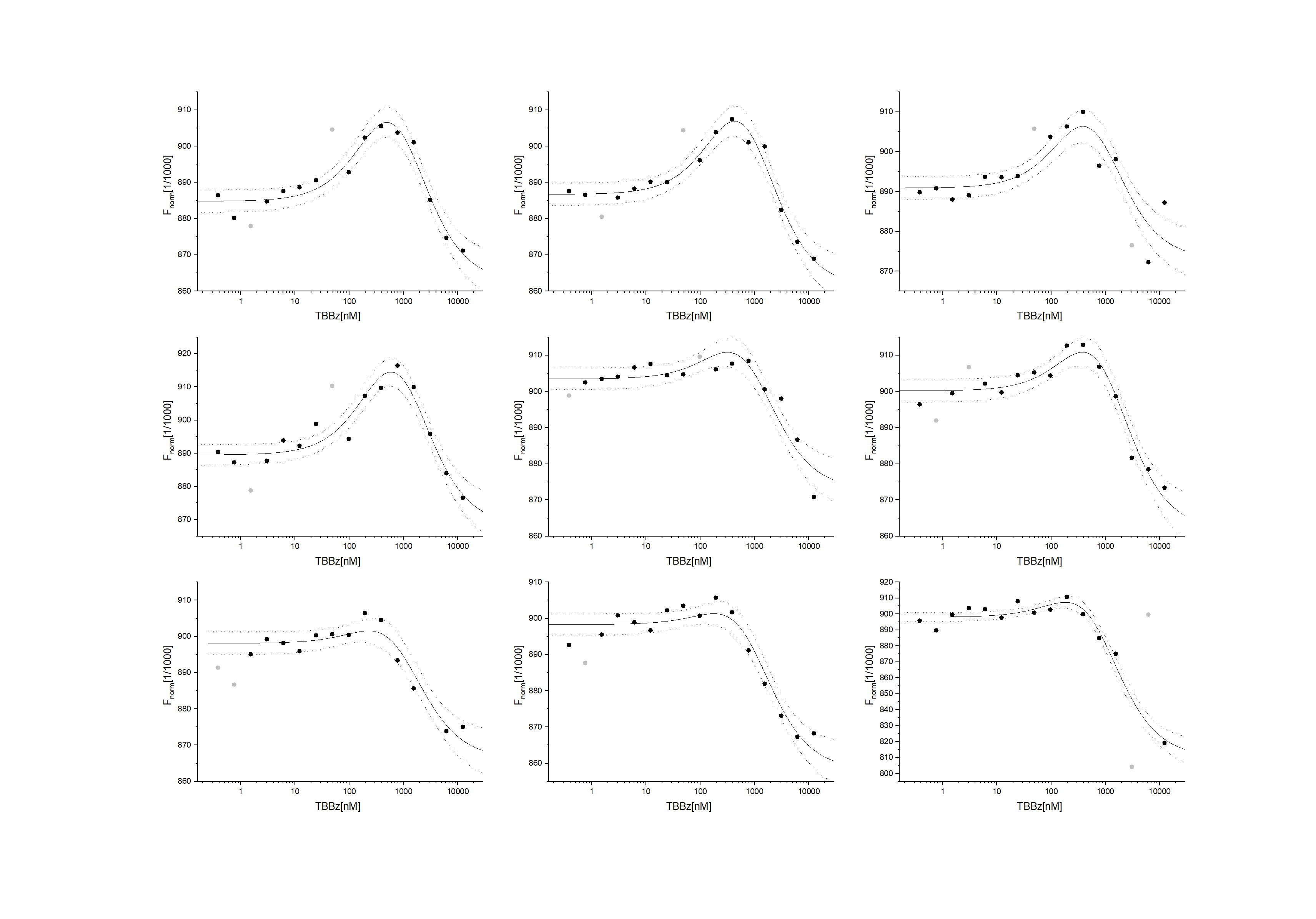
**

**Figure S9.** MST pseudo-titration data collected for **H115F hCK2α and TBBz**. Black circles shows experimental data, grey ones indicate data removed from the analysis, thick line represents the fitted model of two independent biding sites and dotted ones boarder the 95% confidence limits for the model.

**
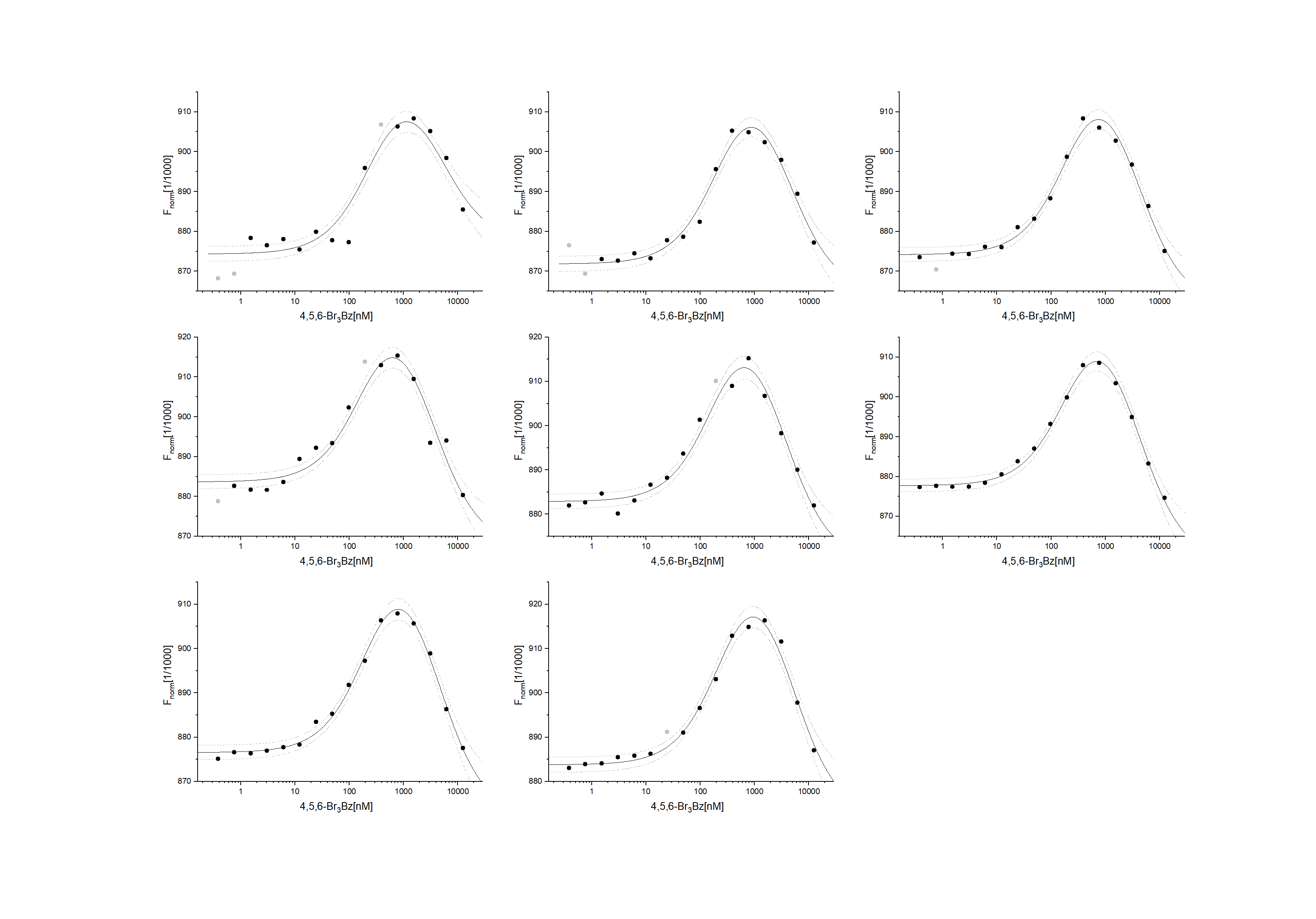
**

**Figure S10.** MST pseudo-titration data collected for **H115F hCK2α and 4,5,6-Br_3_Bz**. Black circles shows experimental data, grey ones indicate data removed from the analysis, thick line represents the fitted model of two independent biding sites and dotted ones boarder the 95% confidence limits for the model.

**
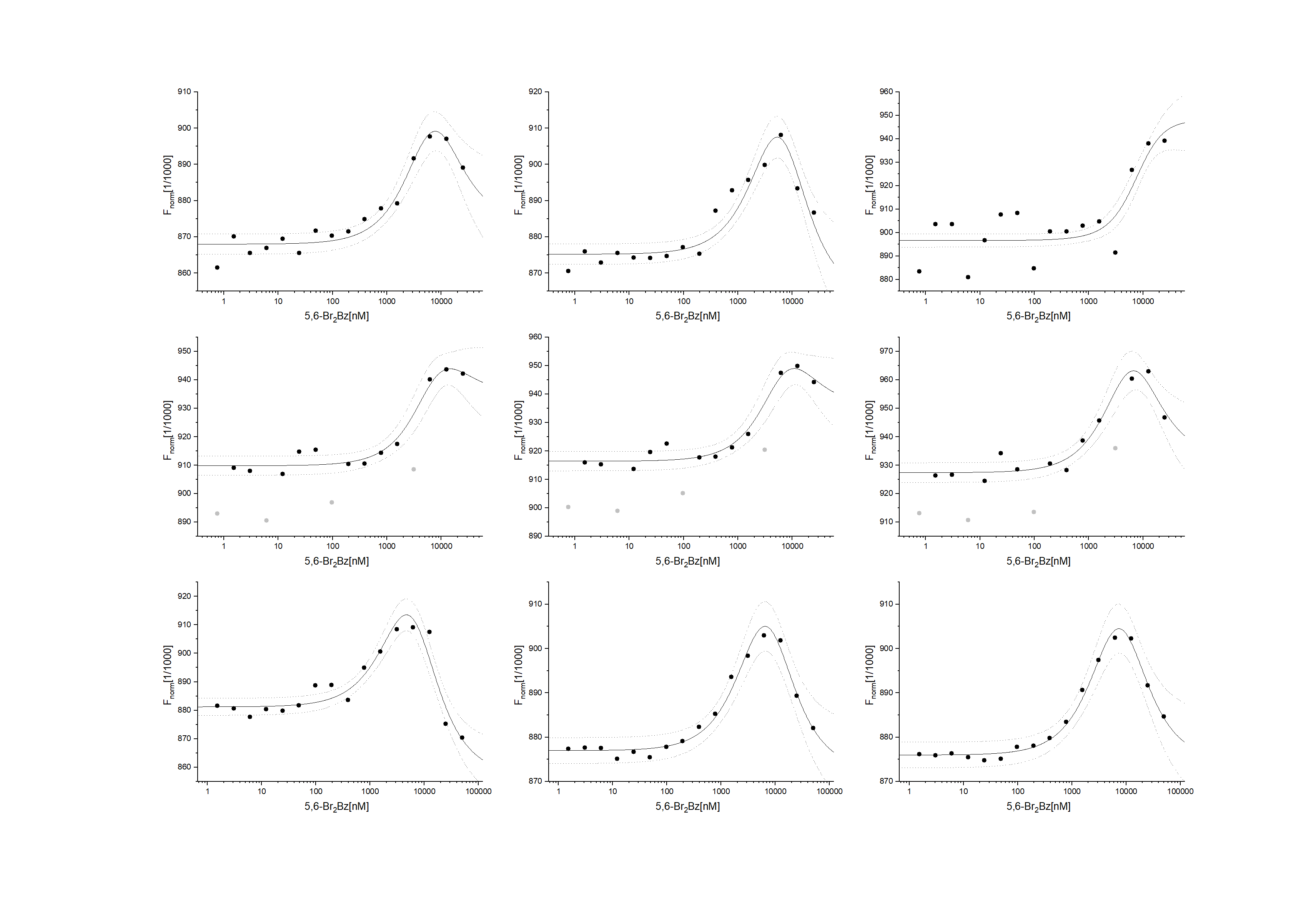
**

**Figure S11.** MST pseudo-titration data collected for **H115F hCK2α and 5,6-Br_2_Bz**. Black circles shows experimental data, grey ones indicate data removed from the analysis, thick line represents the fitted model of two independent biding sites and dotted ones boarder the 95% confidence limits for the model.

**
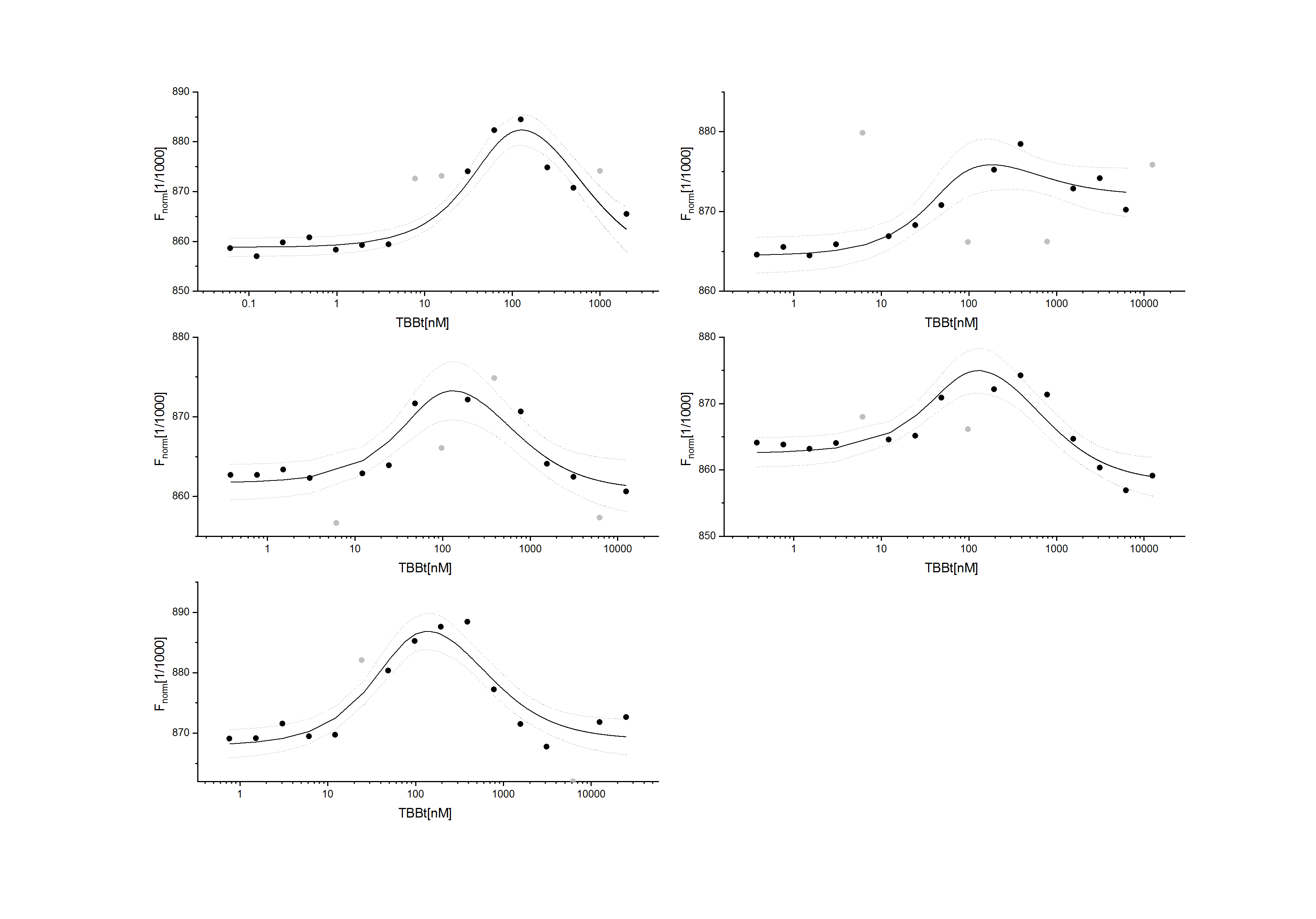
**

**Figure S12.** MST pseudo-titration data collected for **H160A hCK2α and TBBt**. Black circles shows experimental data, grey ones indicate data removed from the analysis, thick line represents the fitted model of two independent biding sites and dotted ones boarder the 95% confidence limits for the model.

**
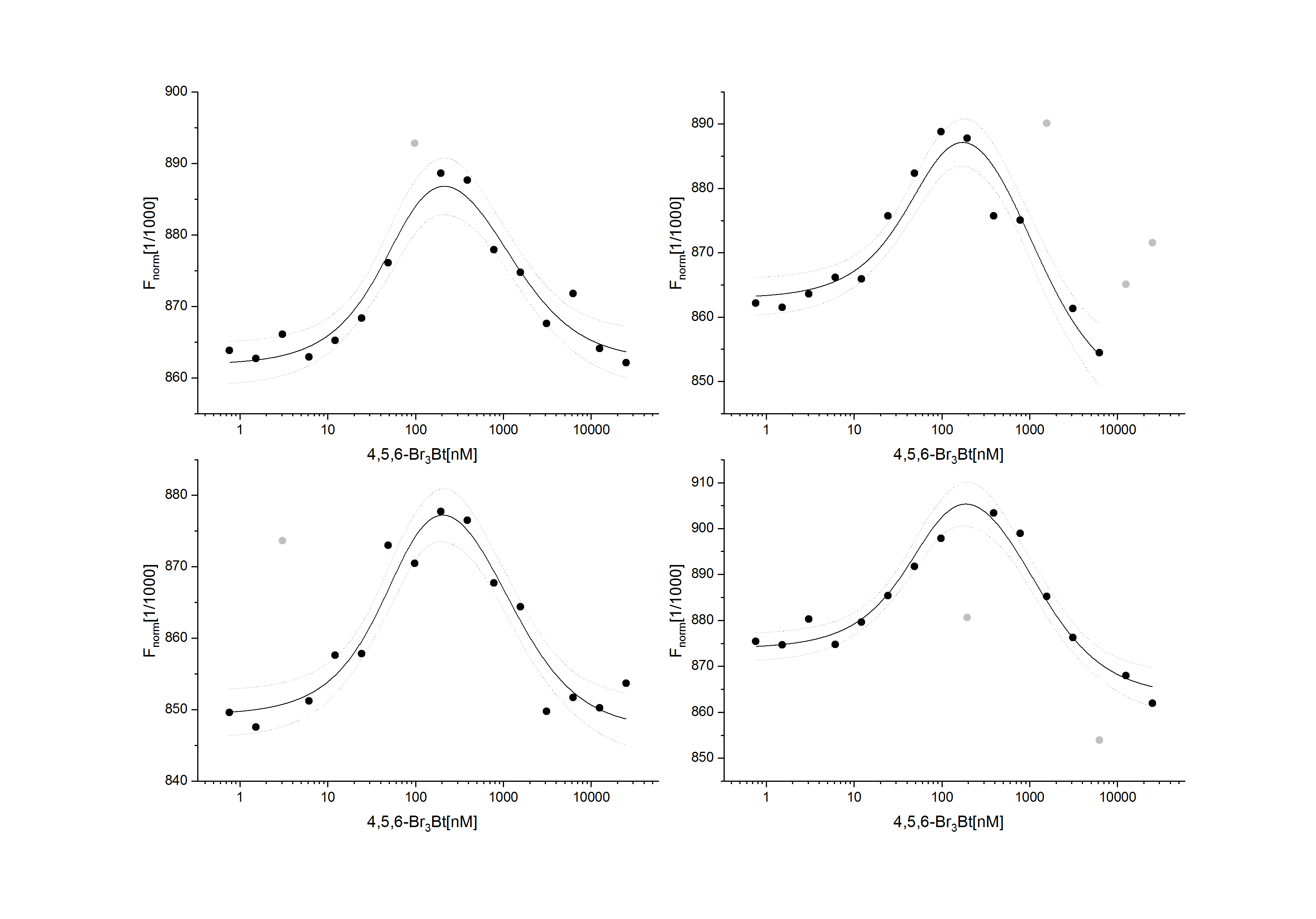
**

**Figure S13.** MST pseudo-titration data collected for **H160A hCK2α and 4,5,6-Br_3_Bt**. Black circles shows experimental data, grey ones indicate data removed from the analysis, thick line represents the fitted model of two independent biding sites and dotted ones boarder the 95% confidence limits for the model.

**
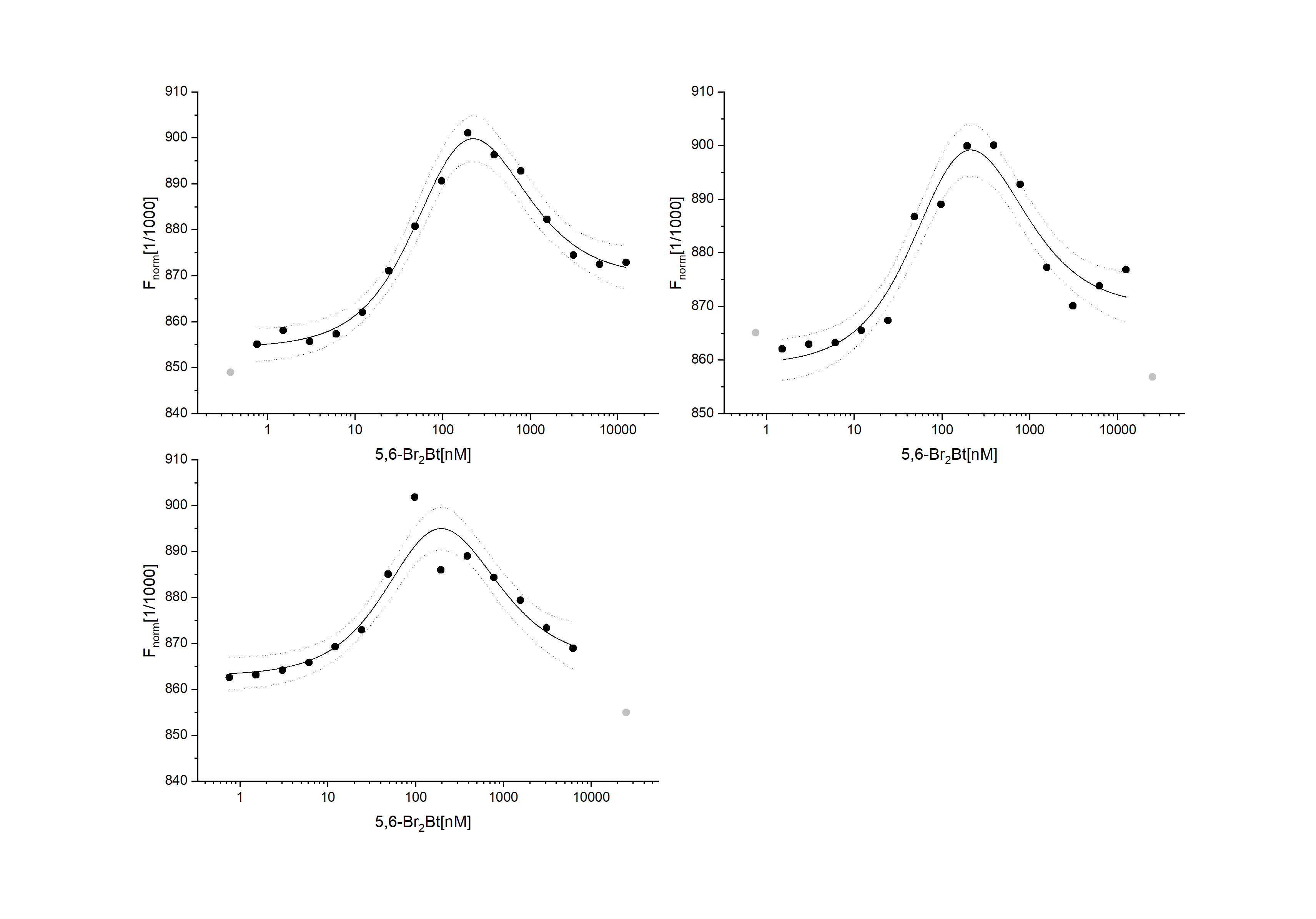
**

**Figure S14.** MST pseudo-titration data collected for **H1160A hCK2α and 5,6-Br_2_Bt**. Black circles shows experimental data, grey ones indicate data removed from the analysis, thick line represents the fitted model of two independent biding sites and dotted ones boarder the 95% confidence limits for the model.

**
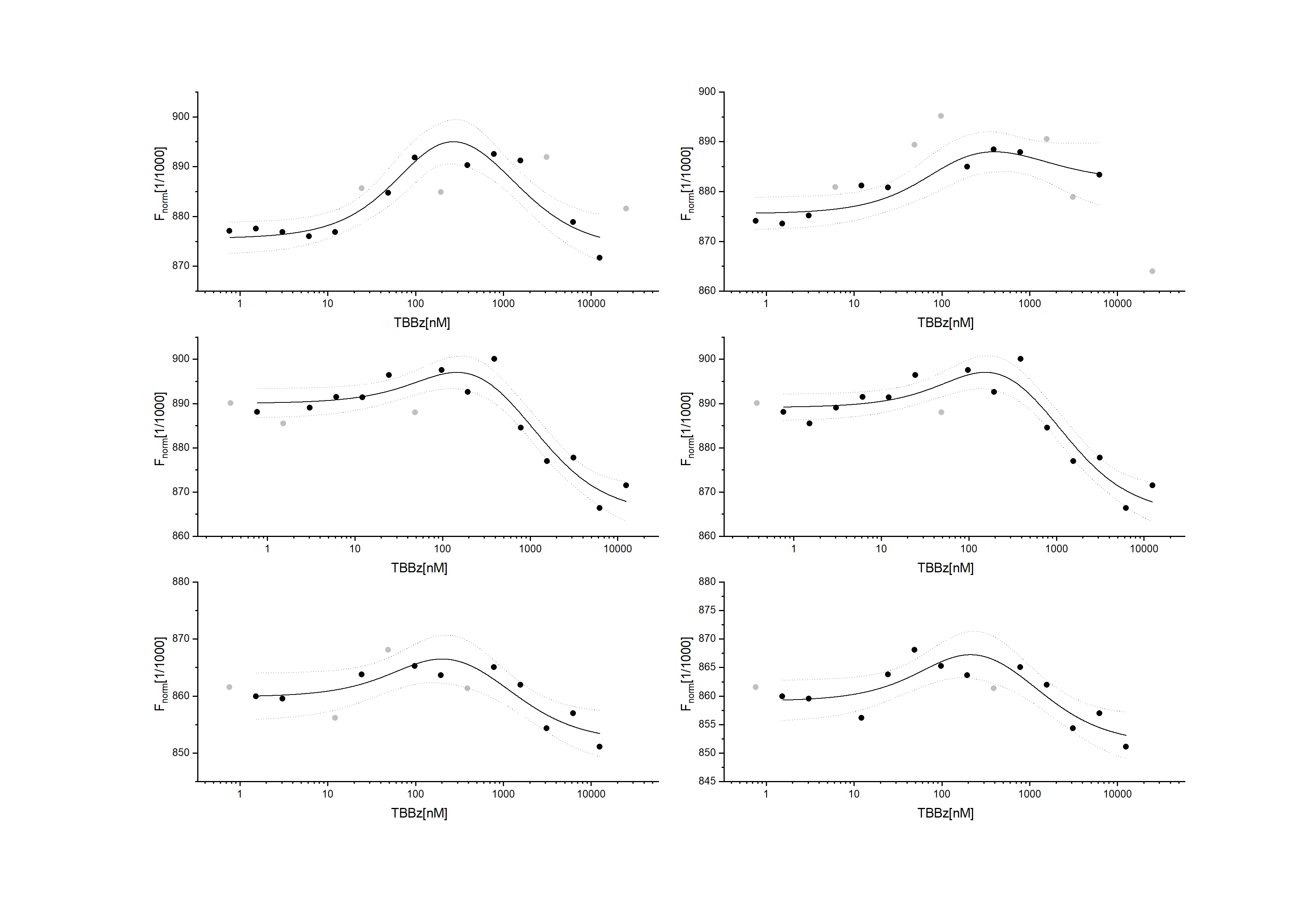
**

**Figure S15.** MST pseudo-titration data collected for **H160A hCK2α and TBBz**. Black circles shows experimental data, grey ones indicate data removed from the analysis, thick line represents the fitted model of two independent biding sites and dotted ones boarder the 95% confidence limits for the model.

**
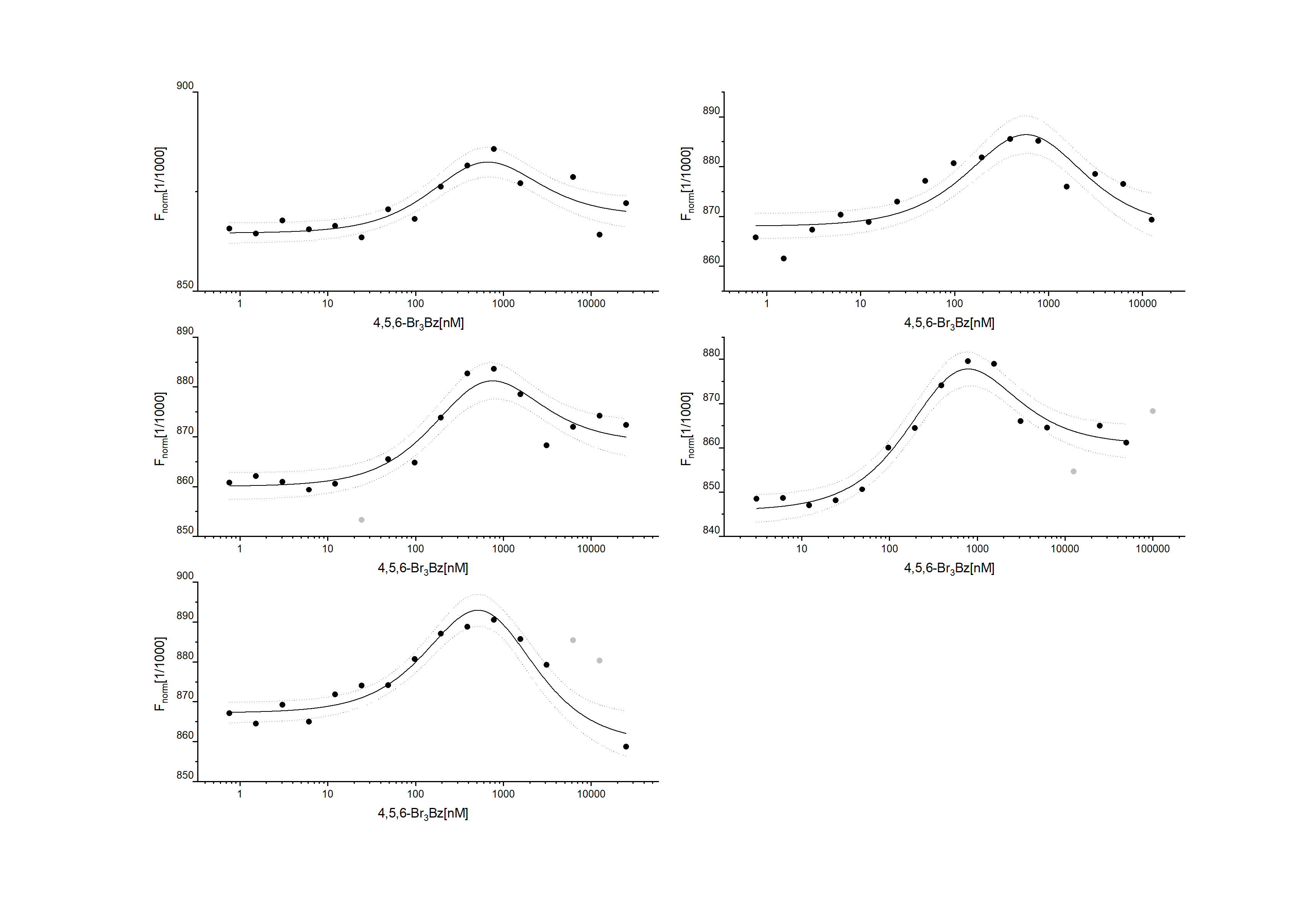
**

**Figure S16.** MST pseudo-titration data collected for **H160A hCK2α and 4,5,6-Br_3_Bz**. Black circles shows experimental data, grey ones indicate data removed from the analysis, thick line represents the fitted model of two independent biding sites and dotted ones boarder the 95% confidence limits for the model.

**
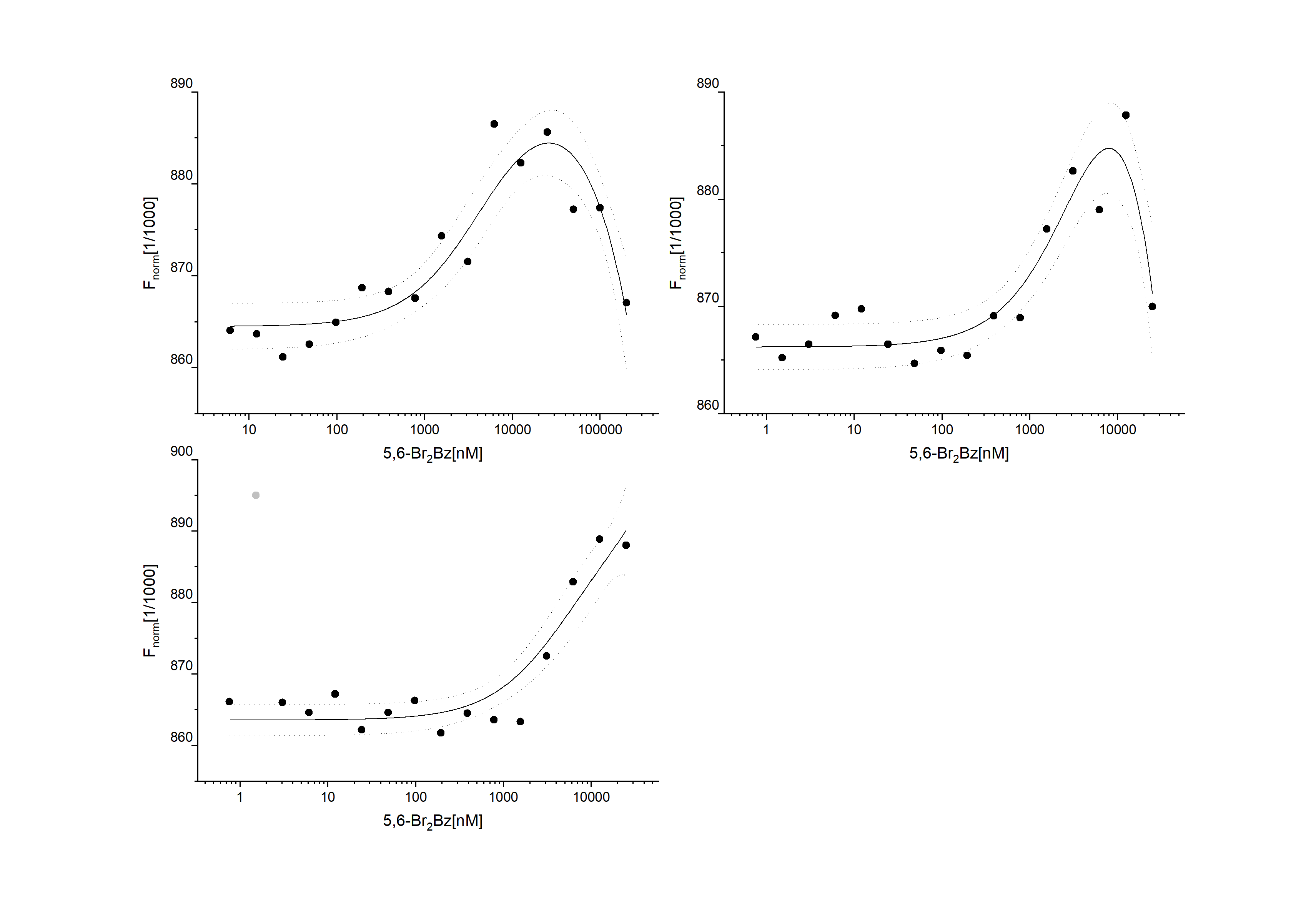
**

**Figure S17.** MST pseudo-titration data collected for **H160A hCK2α and 5,6-Br_2_Bz**. Black circles shows experimental data, grey ones indicate data removed from the analysis, thick line represents the fitted model of two independent biding sites and dotted ones boarder the 95% confidence limits for the model.


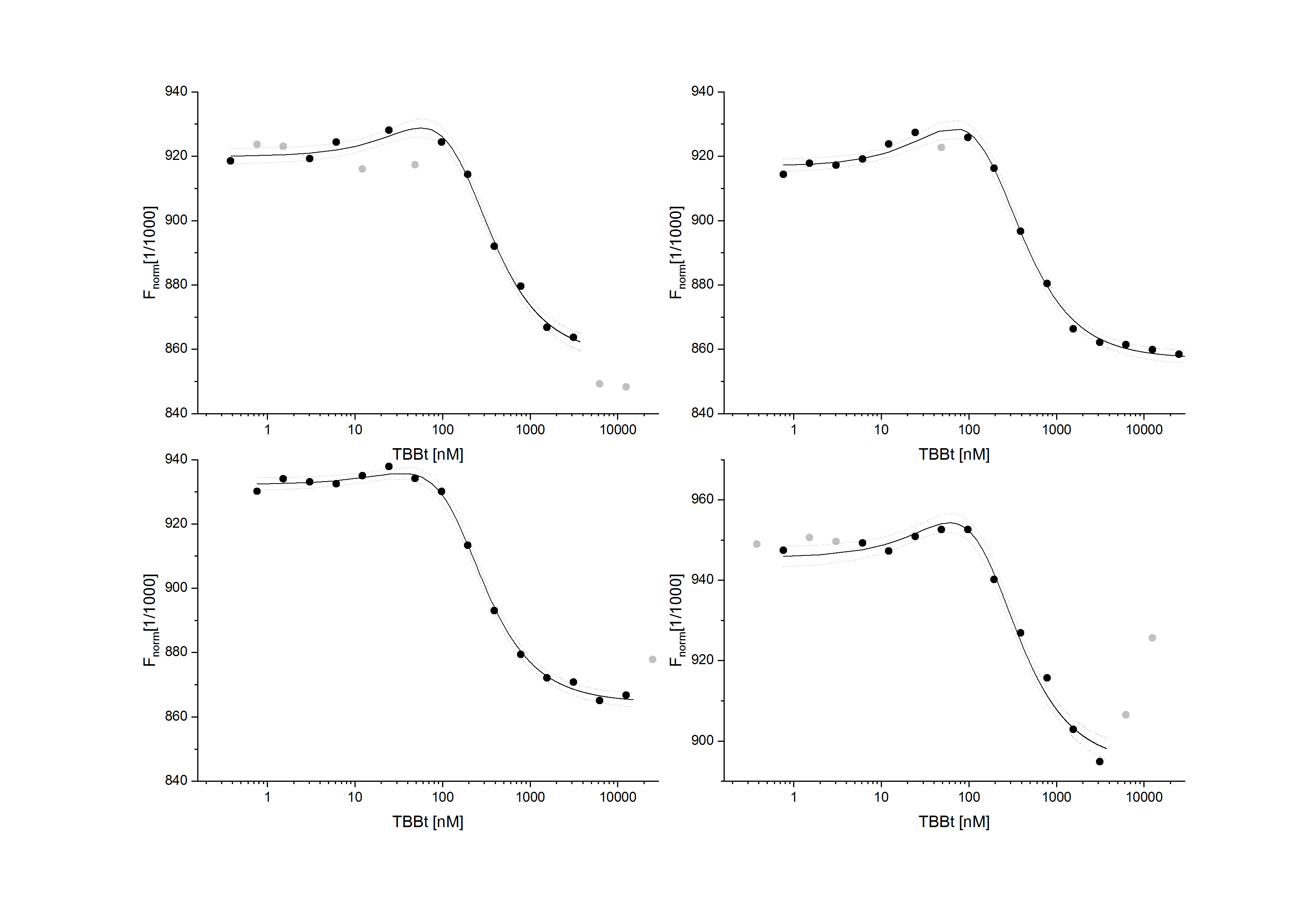


**Figure S18.** MST pseudo-titration data collected for **H160F hCK2α and TBBt**. Black circles shows experimental data, grey ones indicate data removed from the analysis, thick line represents the fitted model of two independent biding sites and dotted ones boarder the 95% confidence limits for the model.

**
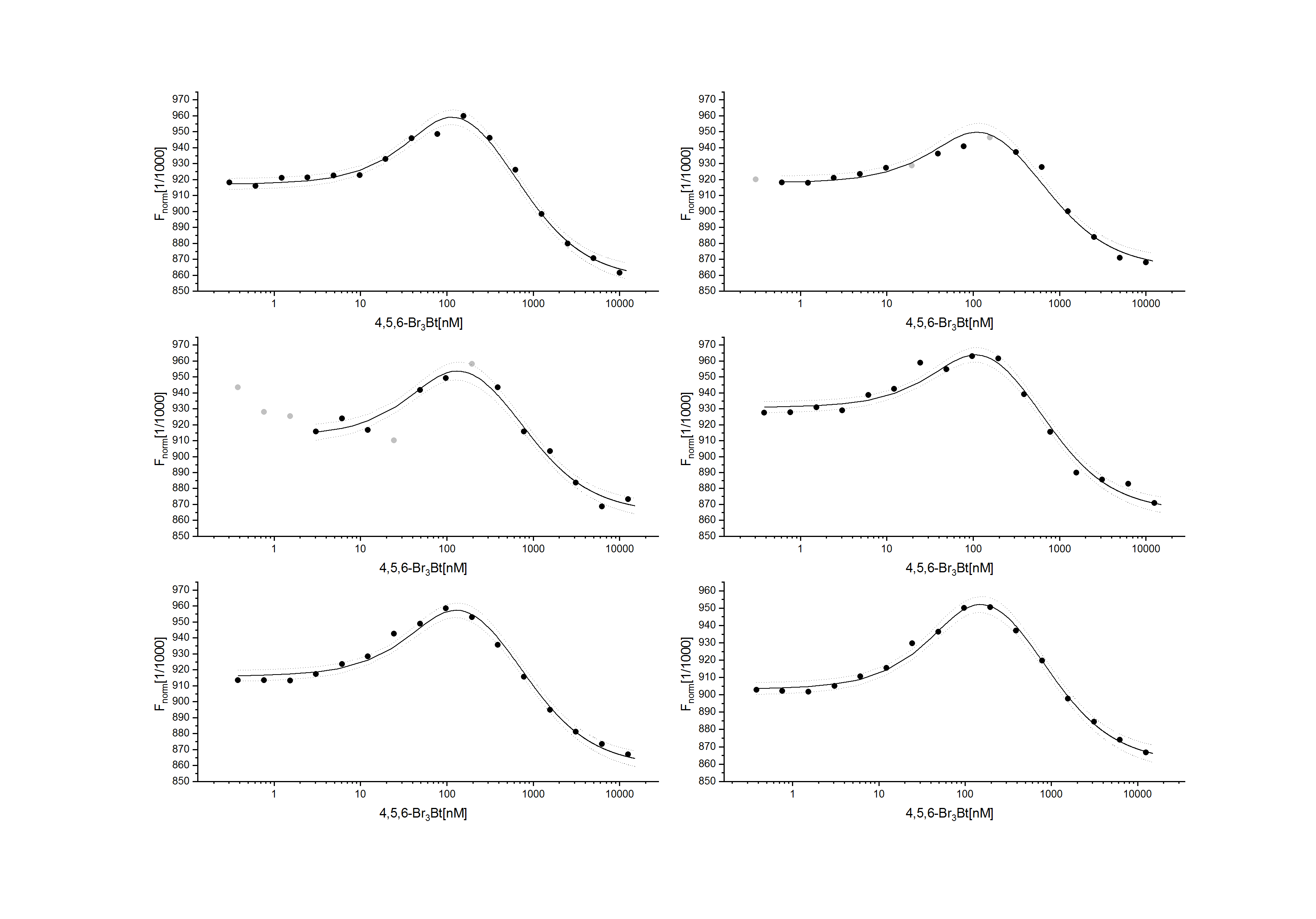
**

**Figure S19.** MST pseudo-titration data collected for **H160F hCK2α and 4,5,6-Br_3_Bt**. Black circles shows experimental data, grey ones indicate data removed from the analysis, thick line represents the fitted model of two independent biding sites and dotted ones boarder the 95% confidence limits for the model.

**
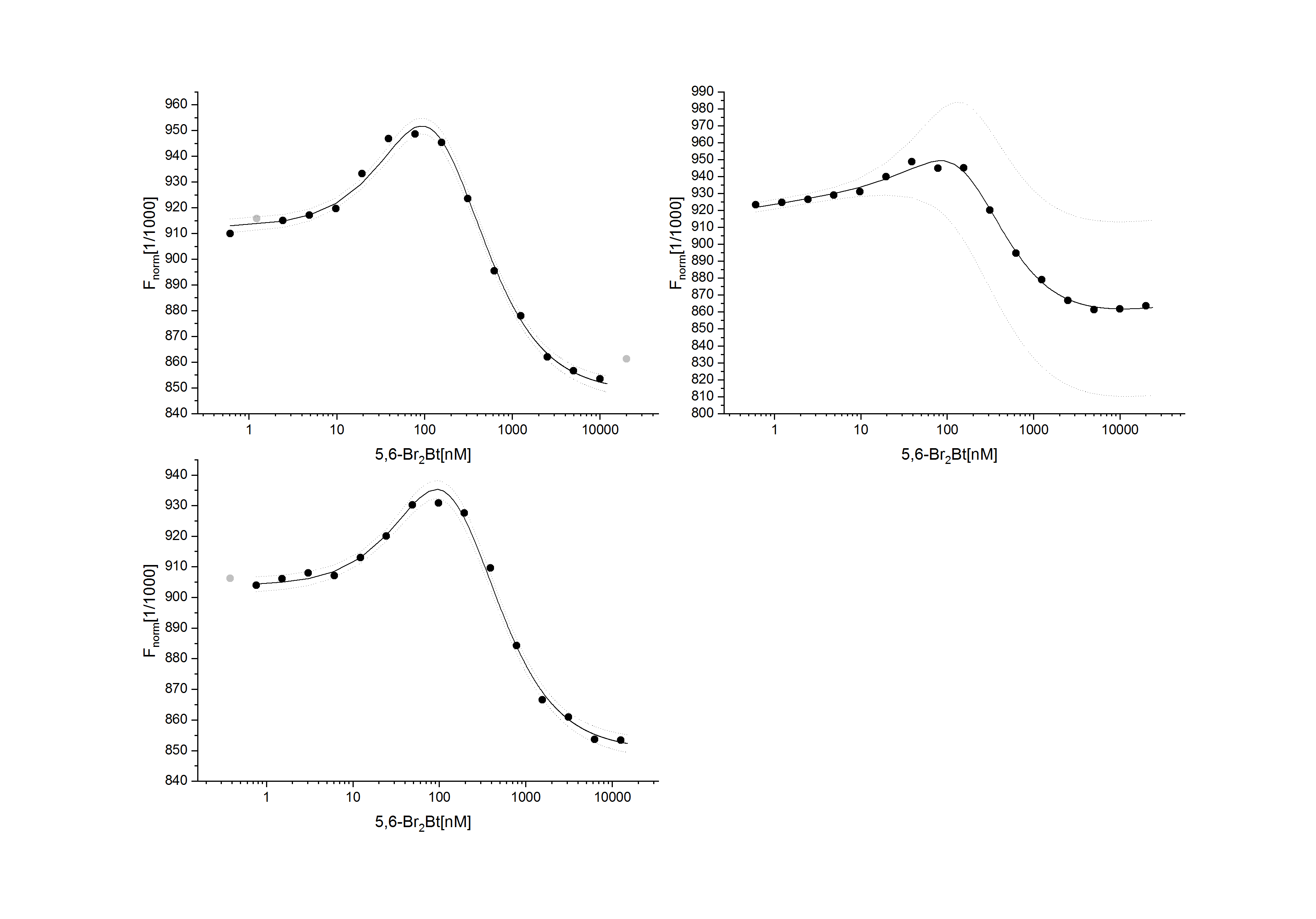
**

**Figure S20.** MST pseudo-titration data collected for **H1160F hCK2α and 5,6-Br_2_Bt**. Black circles shows experimental data, grey ones indicate data removed from the analysis, thick line represents the fitted model of two independent biding sites and dotted ones boarder the 95% confidence limits for the model.


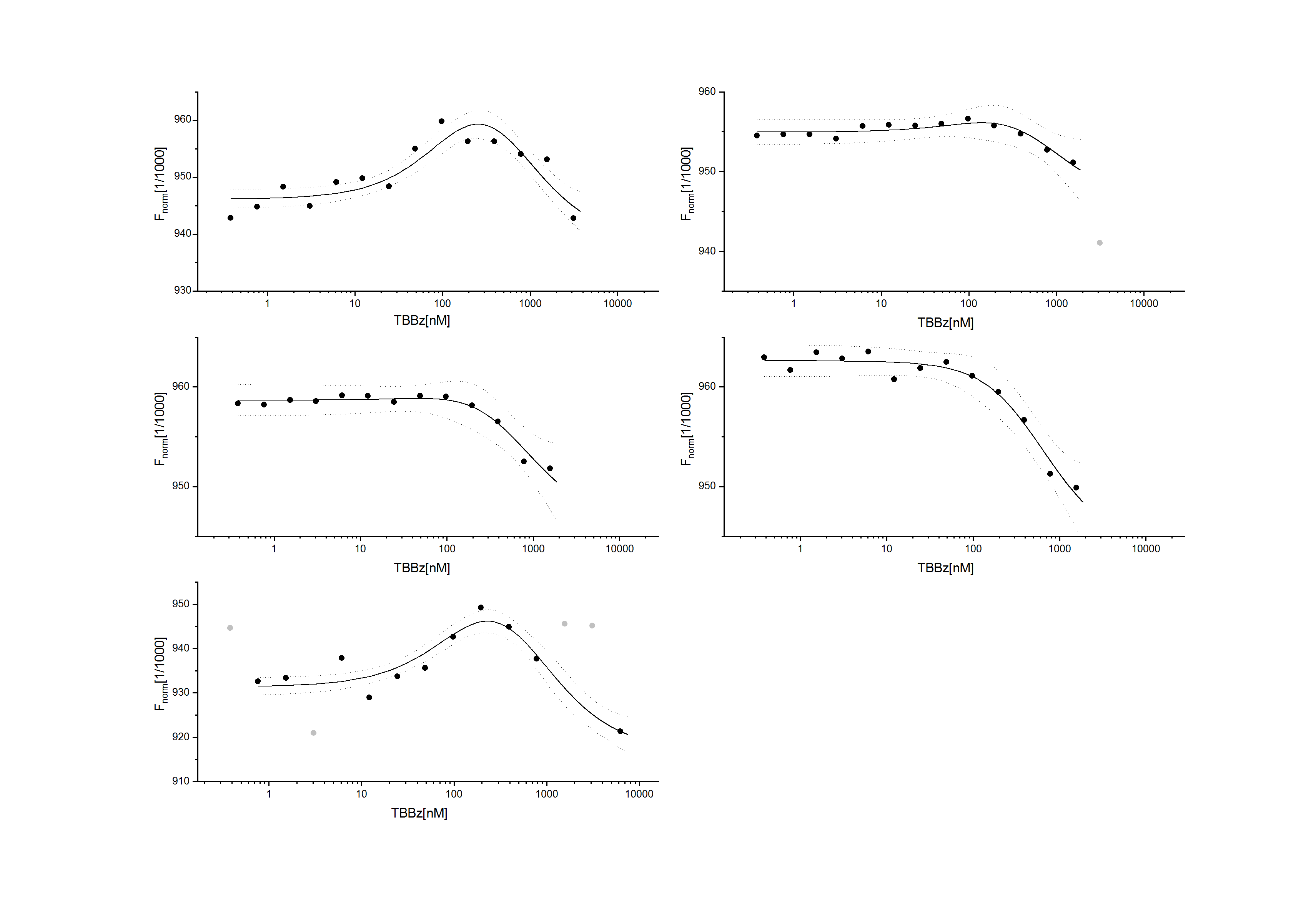


**Figure S21.** MST pseudo-titration data collected for **H160F hCK2α and TBBz**. Black circles shows experimental data, grey ones indicate data removed from the analysis, thick line represents the fitted model of two independent biding sites and dotted ones boarder the 95% confidence limits for the model.


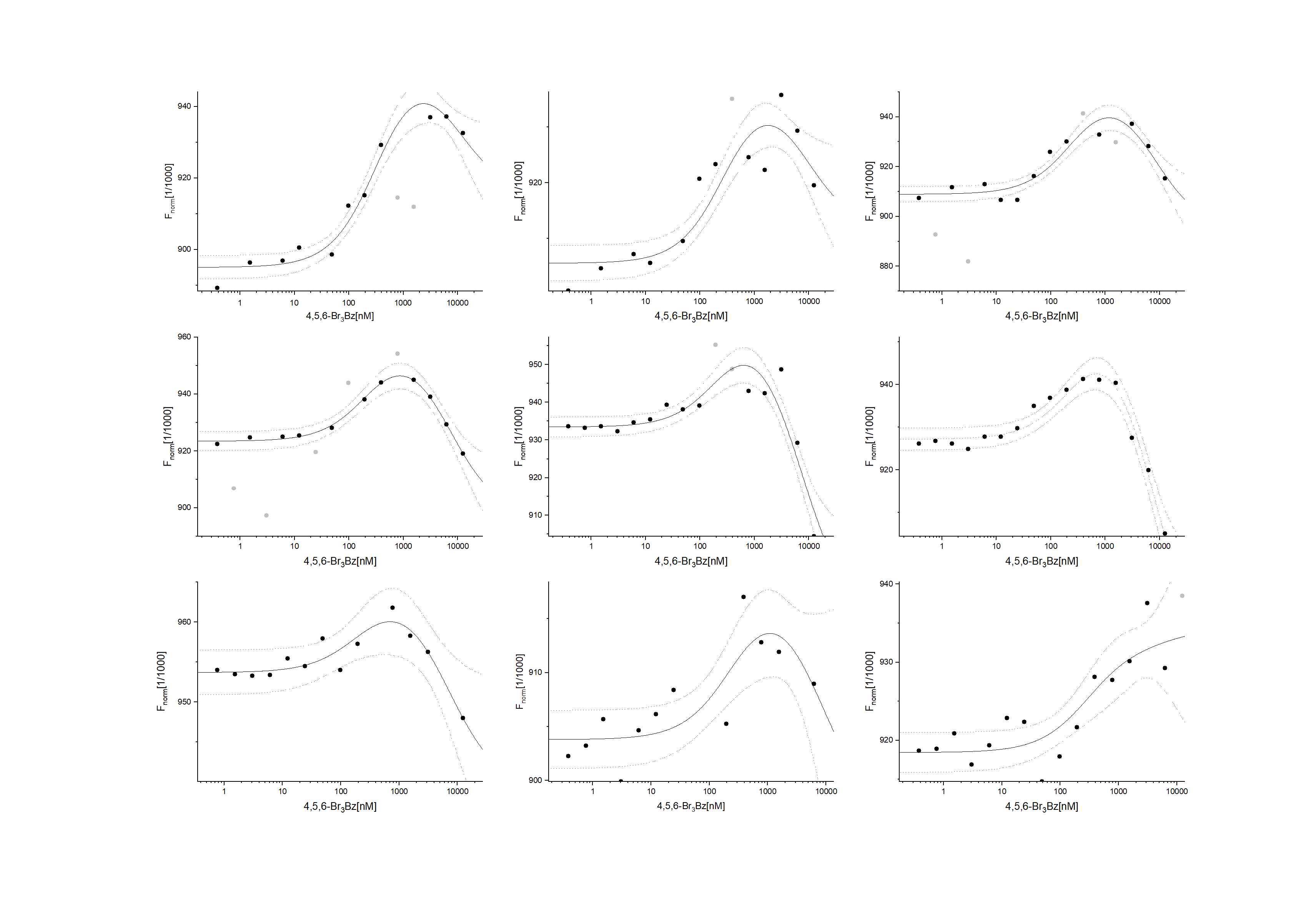


**Figure S22.** MST pseudo-titration data collected for **H160F hCK2α and 4,5,6-Br_3_Bz**. Black circles shows experimental data, grey ones indicate data removed from the analysis, thick line represents the fitted model of two independent biding sites and dotted ones boarder the 95% confidence limits for the model.

**
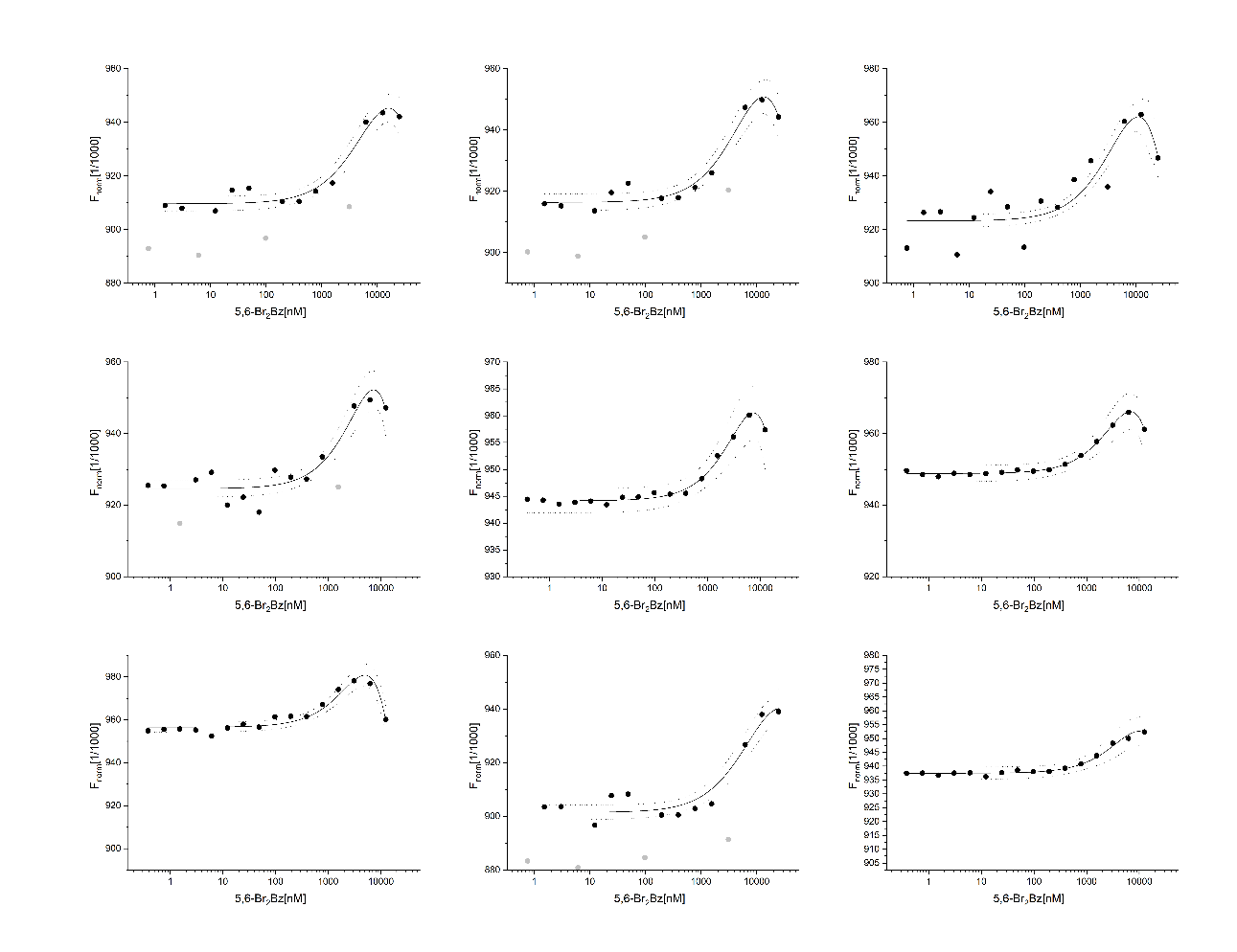
**

**Figure S23.** MST pseudo-titration data collected for **H160F hCK2α and 5,6-Br_2_Bz**. Black circles shows experimental data, grey ones indicate data removed from the analysis, thick line represents the fitted model of two independent biding sites and dotted ones boarder the 95% confidence limits for the model.


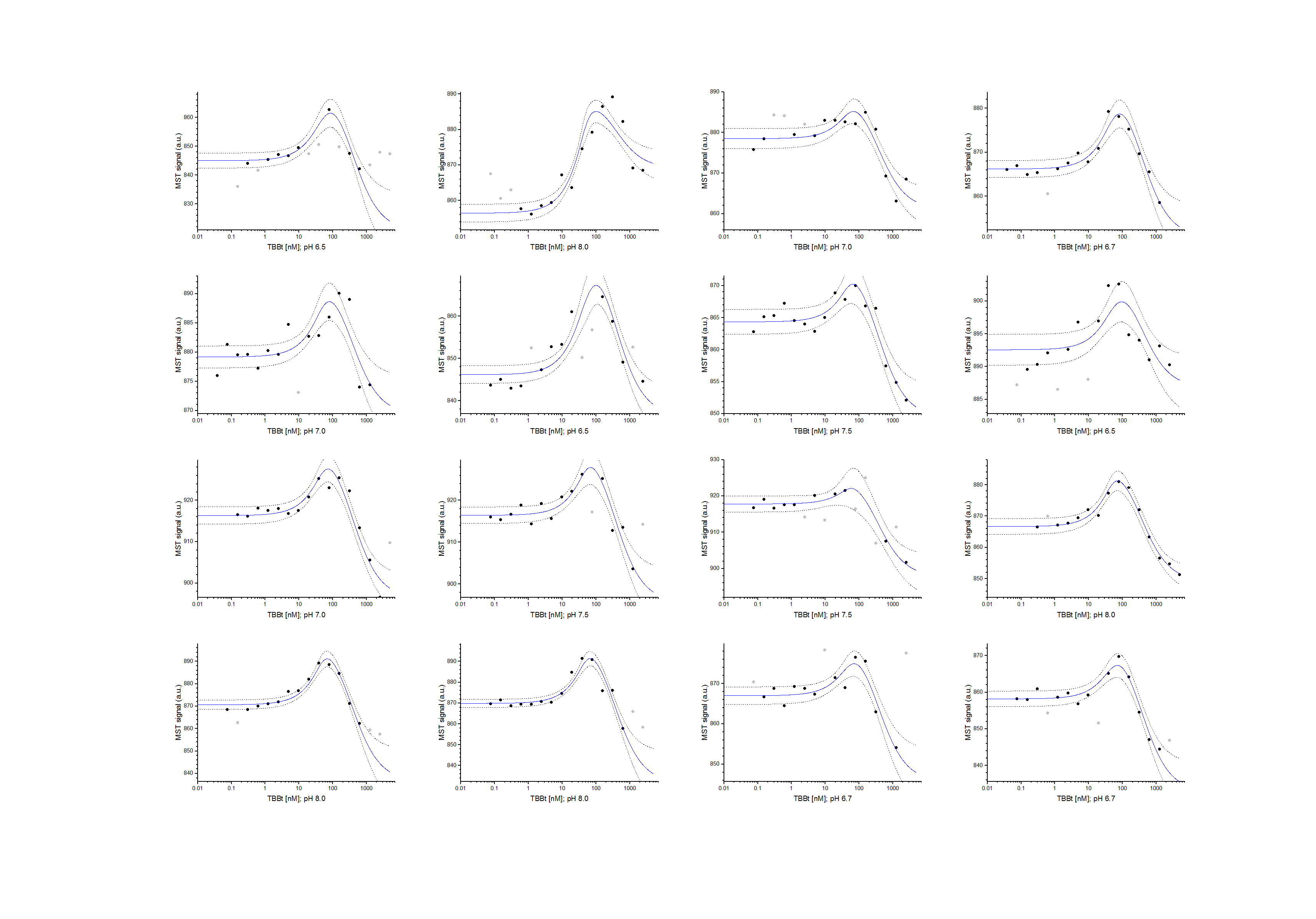


**Figure S24.** MST pseudo-titration data collected for **WT hCK2α and TBBt at different pH**. Black circles shows experimental data, grey ones indicate data removed from the analysis, thick line represents the fitted model of two independent biding sites and dotted ones boarder the 95% confidence limits for the model.

**
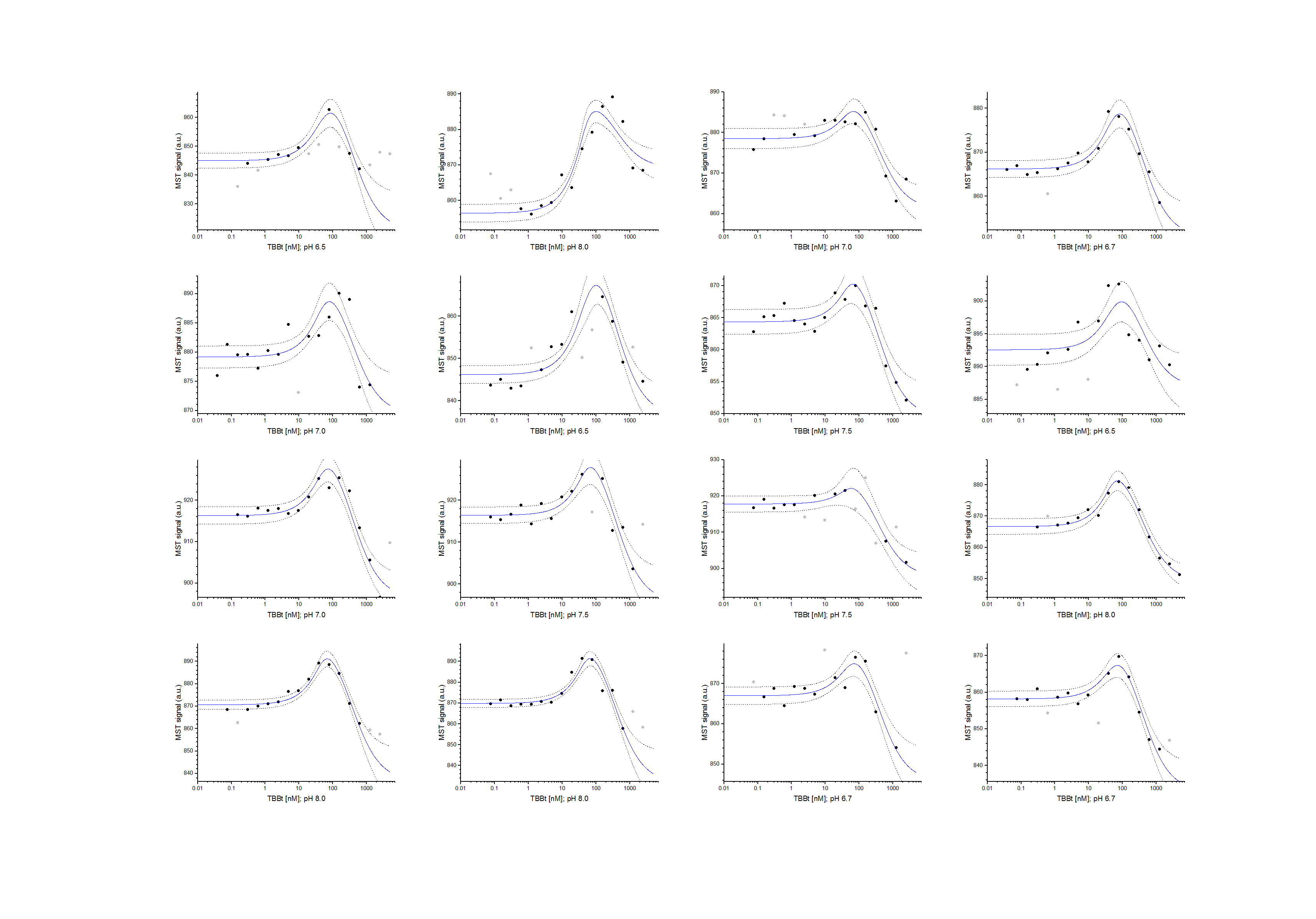
**

**Figure S25.** MST pseudo-titration data collected for **WT hCK2α and 5,6-Br_2_Bt at different pH**. Black circles shows experimental data, grey ones indicate data removed from the analysis, thick blue line represents the fitted model of two independent biding sites and dotted ones boarder the 95% confidence limits for the model.

**
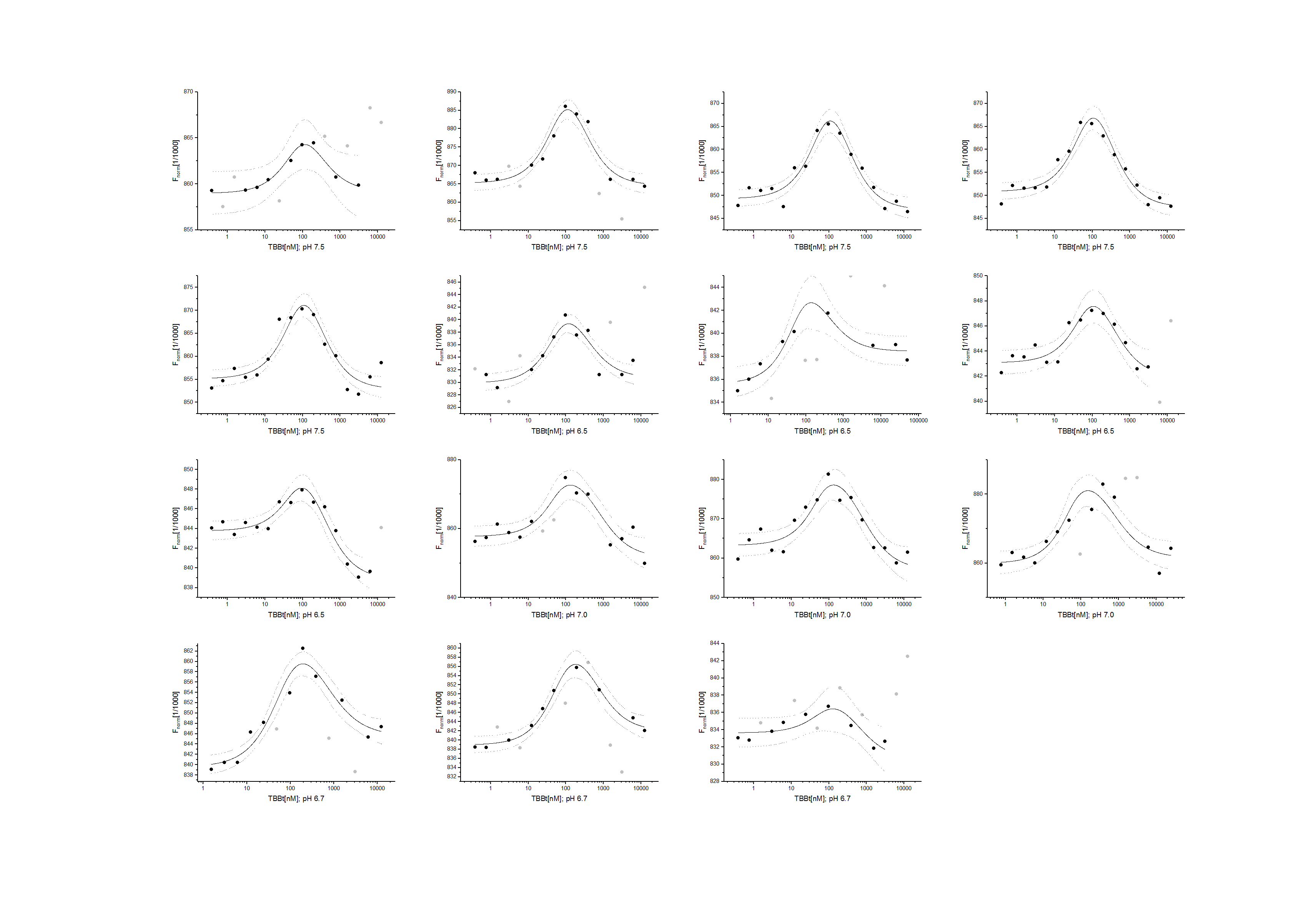
**

**Figure S26.** MST pseudo-titration data collected for **H160A hCK2α and TBBt at different pH**. Black circles shows experimental data, grey ones indicate data removed from the analysis, thick line represents the fitted model of two independent biding sites and dotted ones boarder the 95% confidence limits for the model.


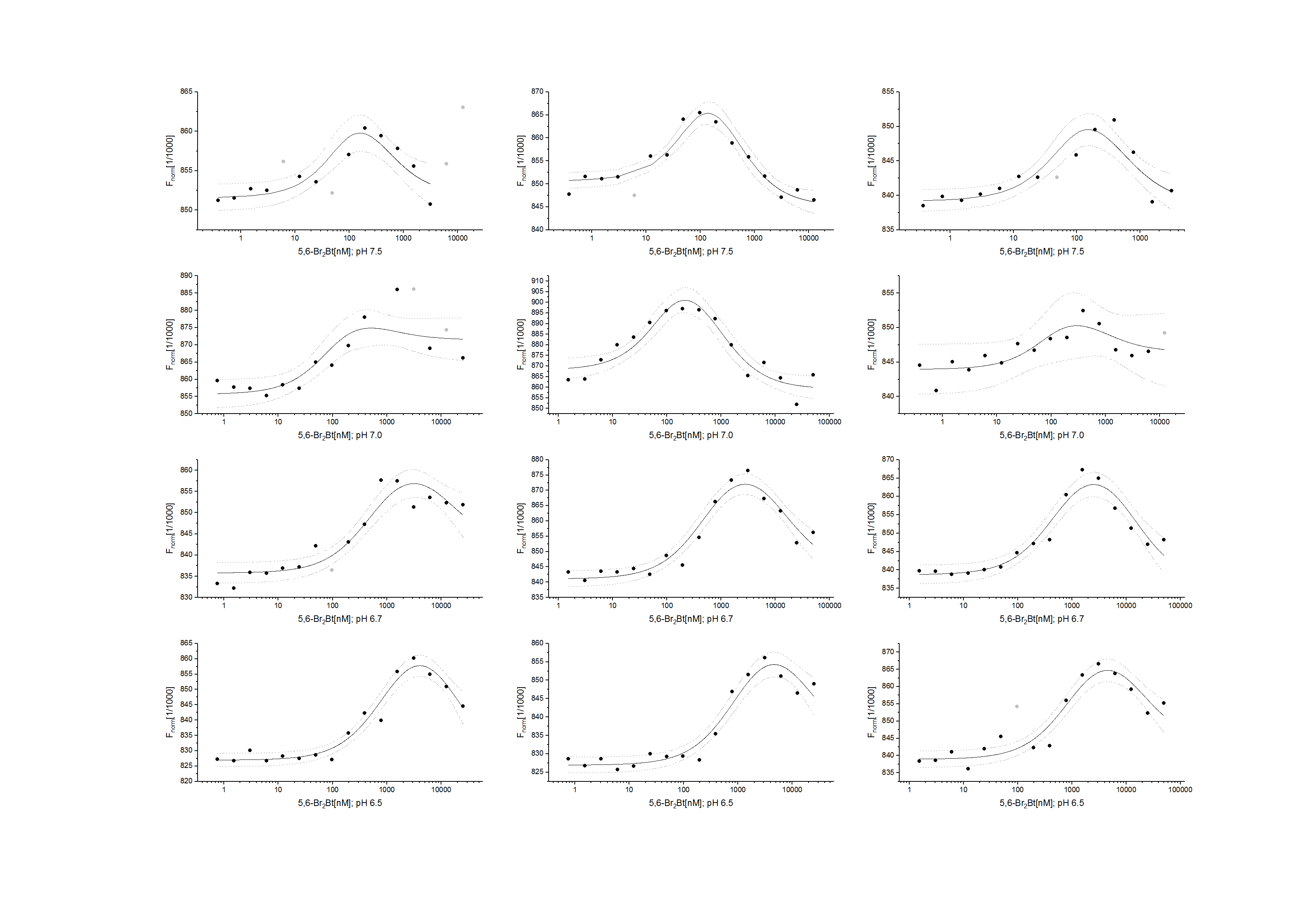


**Figure S27.** MST pseudo-titration data collected for **H160A hCK2α and 5,6-Br_2_Bt at different pH**. Black circles shows experimental data, grey ones indicate data removed from the analysis, thick line represents the fitted model of two independent biding sites and dotted ones boarder the 95% confidence limits for the model.

**
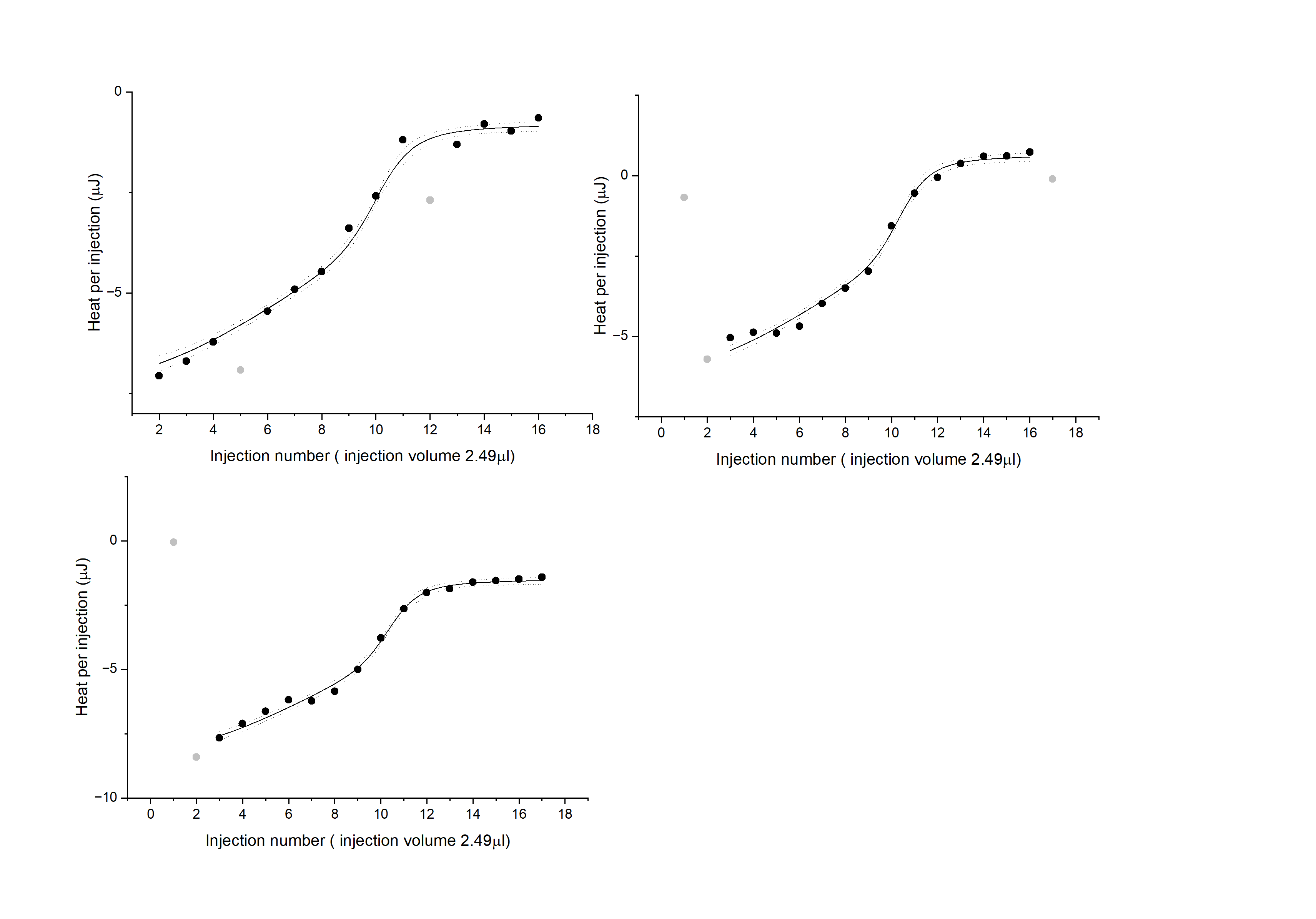
**

**Figure S28.** ITC titration data collected for **WT hCK2α and TBBt**. Black circles shows experimental data, grey ones indicate data removed from the analysis, thick line represents the fitted model of two independent biding sites and dotted ones boarder the 95% confidence limits for the model. First injection with volume 0.1μl was always removed from the analysis.


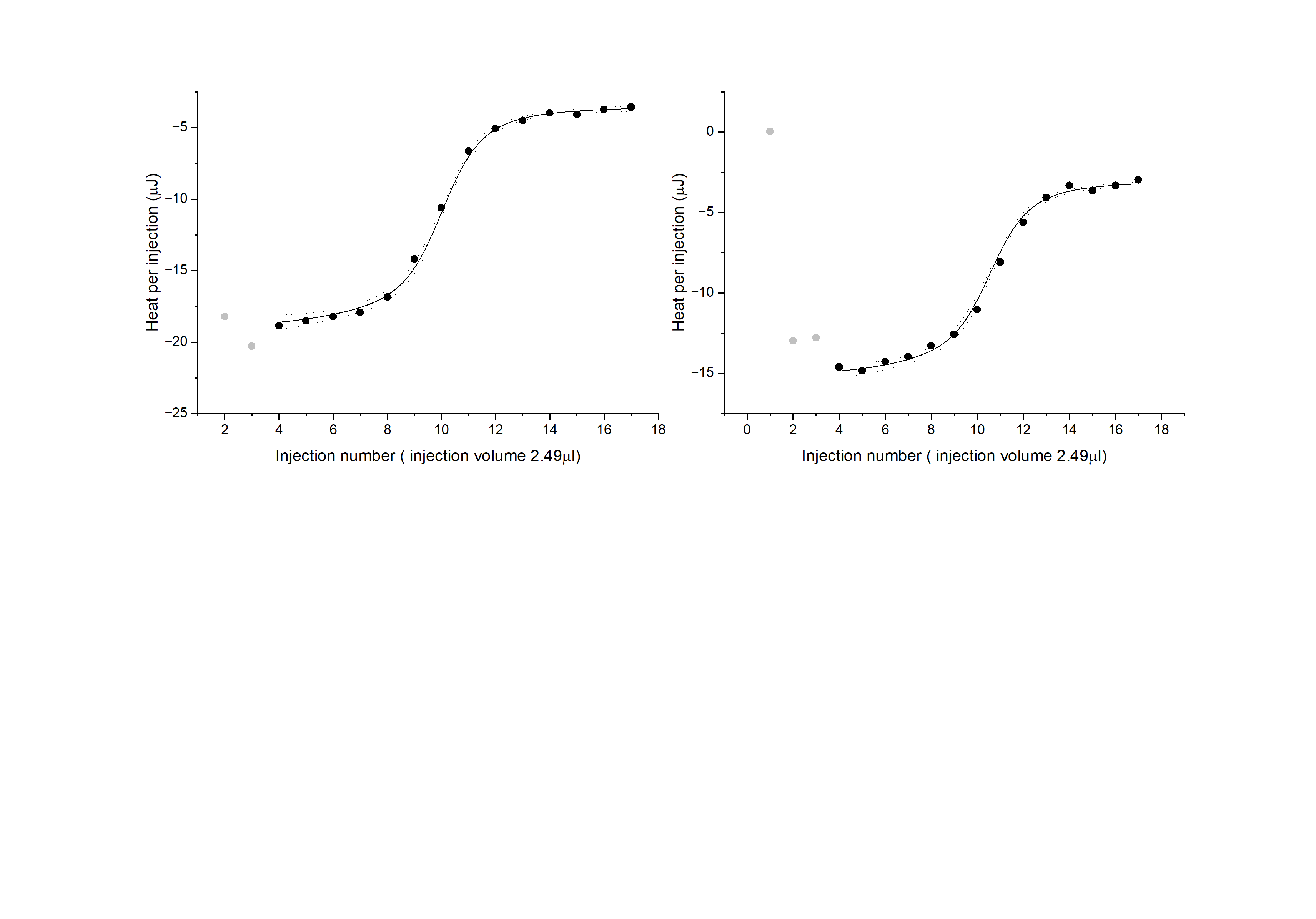


**Figure S29.** ITC titration data collected for **WT hCK2α and 4,5,6-Br_3_Bt.** Black circles shows experimental data, grey ones indicate data removed from the analysis, thick line represents the fitted model of two independent biding sites and dotted ones boarder the 95% confidence limits for the model. First injection with volume 0.1μl was always removed from the analysis.


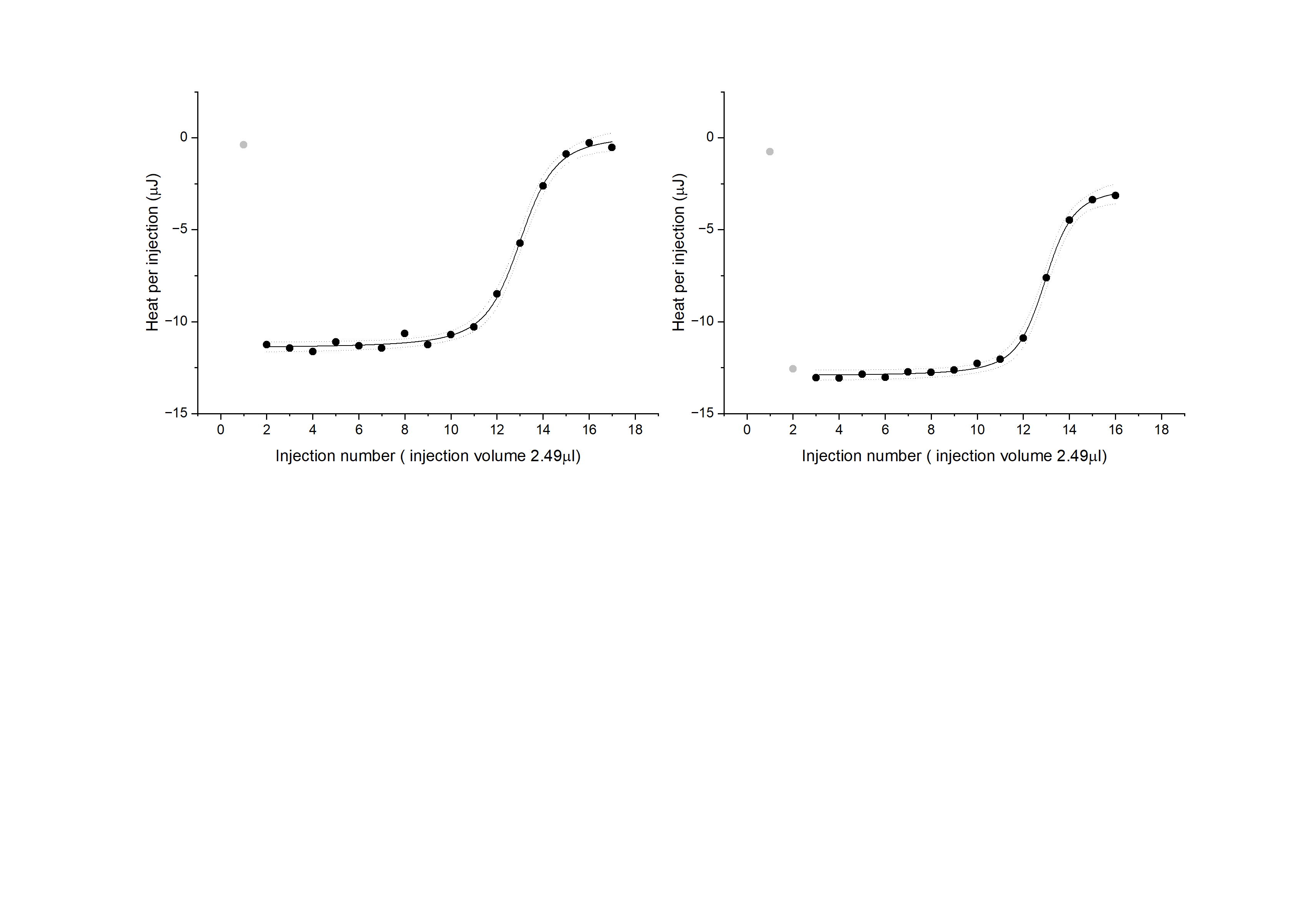


**Figure S30.** ITC titration data collected for **WT hCK2α and 5,6-Br_2_Bt.** Black circles shows experimental data, grey ones indicate data removed from the analysis, thick line represents the fitted model of two independent biding sites and dotted ones boarder the 95% confidence limits for the model. First injection with volume 0.1μl was always removed from the analysis.

**
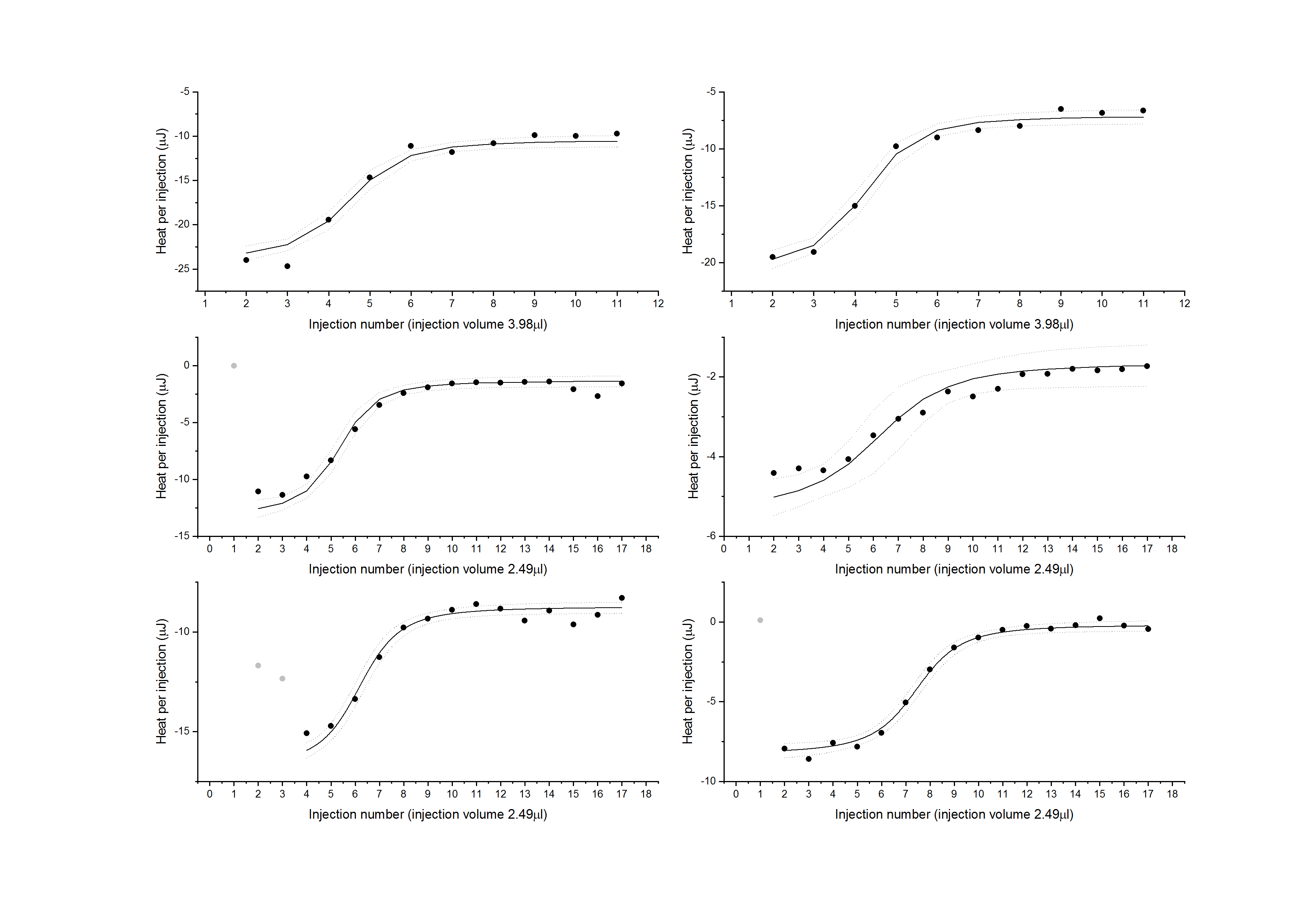
**

**Figure S31.** ITC titration data collected for **WT hCK2α and TBBz**..Black circles shows experimental data, grey ones indicate data removed from the analysis, thick line represents the fitted model of two independent biding sites and dotted ones boarder the 95% confidence limits for the model. First injection with volume 0.1μl was always removed from the analysis.

**
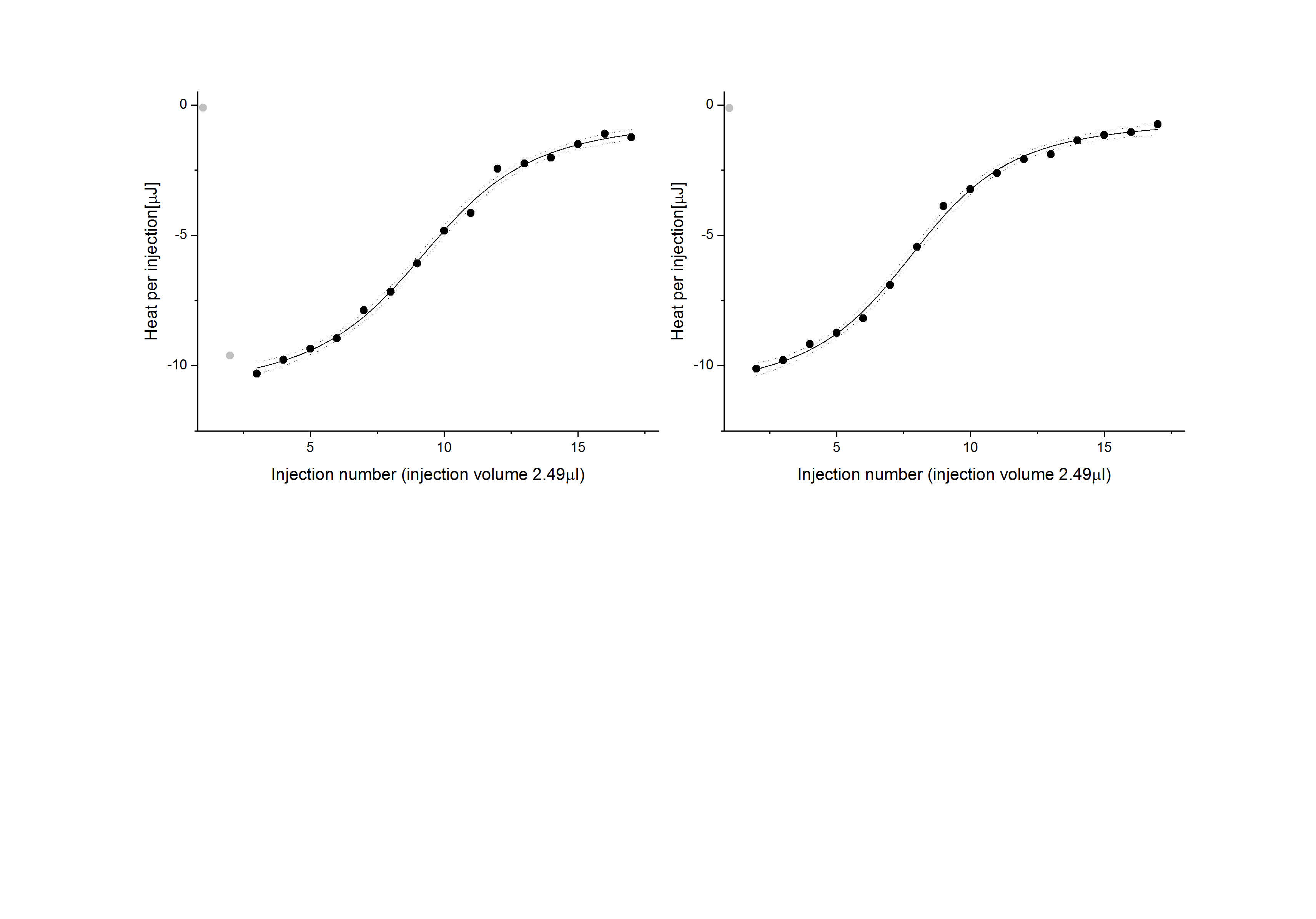
**

**Figure S32.** ITC titration data collected for **WT hCK2α and 4,5,6-Br_3_Bz**. Black circles shows experimental data, grey ones indicate data removed from the analysis, thick line represents the fitted model of two independent biding sites and dotted ones boarder the 95% confidence limits for the model. First injection with volume 0.1μl was always removed from the analysis.

**
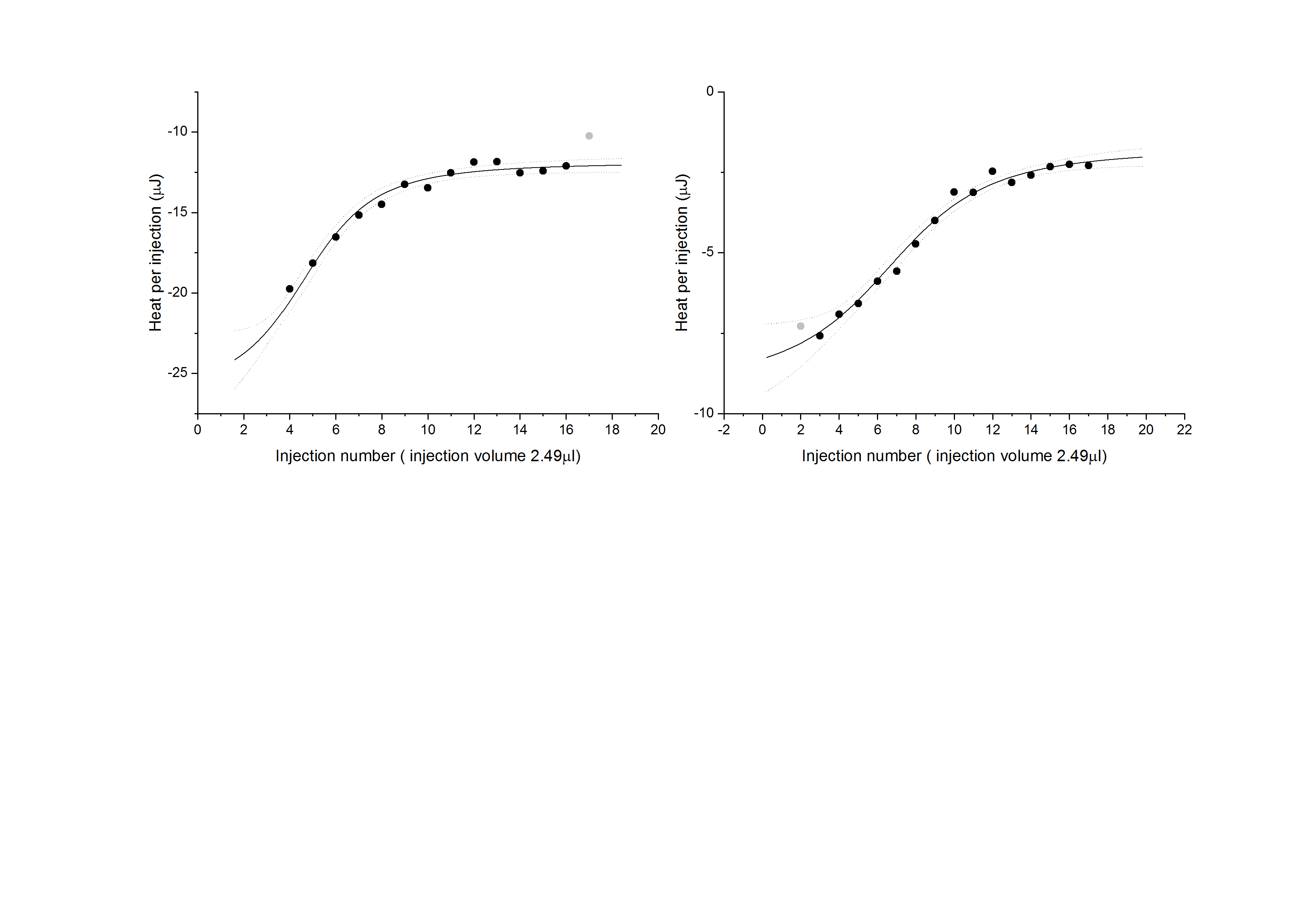
**

**Figure S33.** ITC titration data collected for **WT hCK2α and 5,6-Br_2_Bz**. Black circles shows experimental data, grey ones indicate data removed from the analysis, thick line represents the fitted model of two independent biding sites and dotted ones boarder the 95% confidence limits for the model. First injection with volume 0.1μl was always removed from the analysis.

**
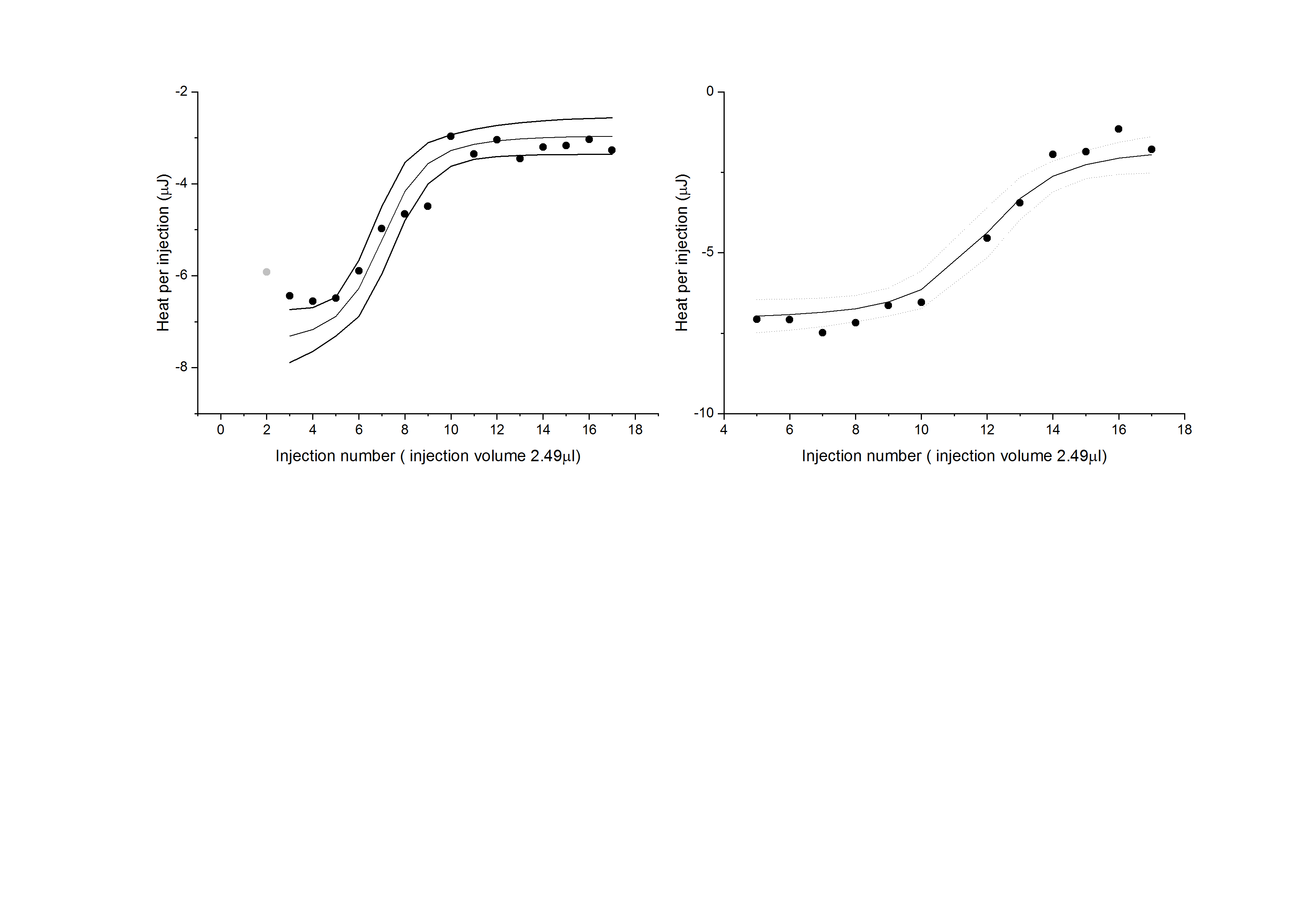
**

**Figure S34.** ITC titration data collected for **H115A hCK2α and TBBt**. Black circles shows experimental data, grey ones indicate data removed from the analysis, thick line represents the fitted model of two independent biding sites and dotted ones boarder the 95% confidence limits for the model. First injection with volume 0.1μl was always removed from the analysis.

**
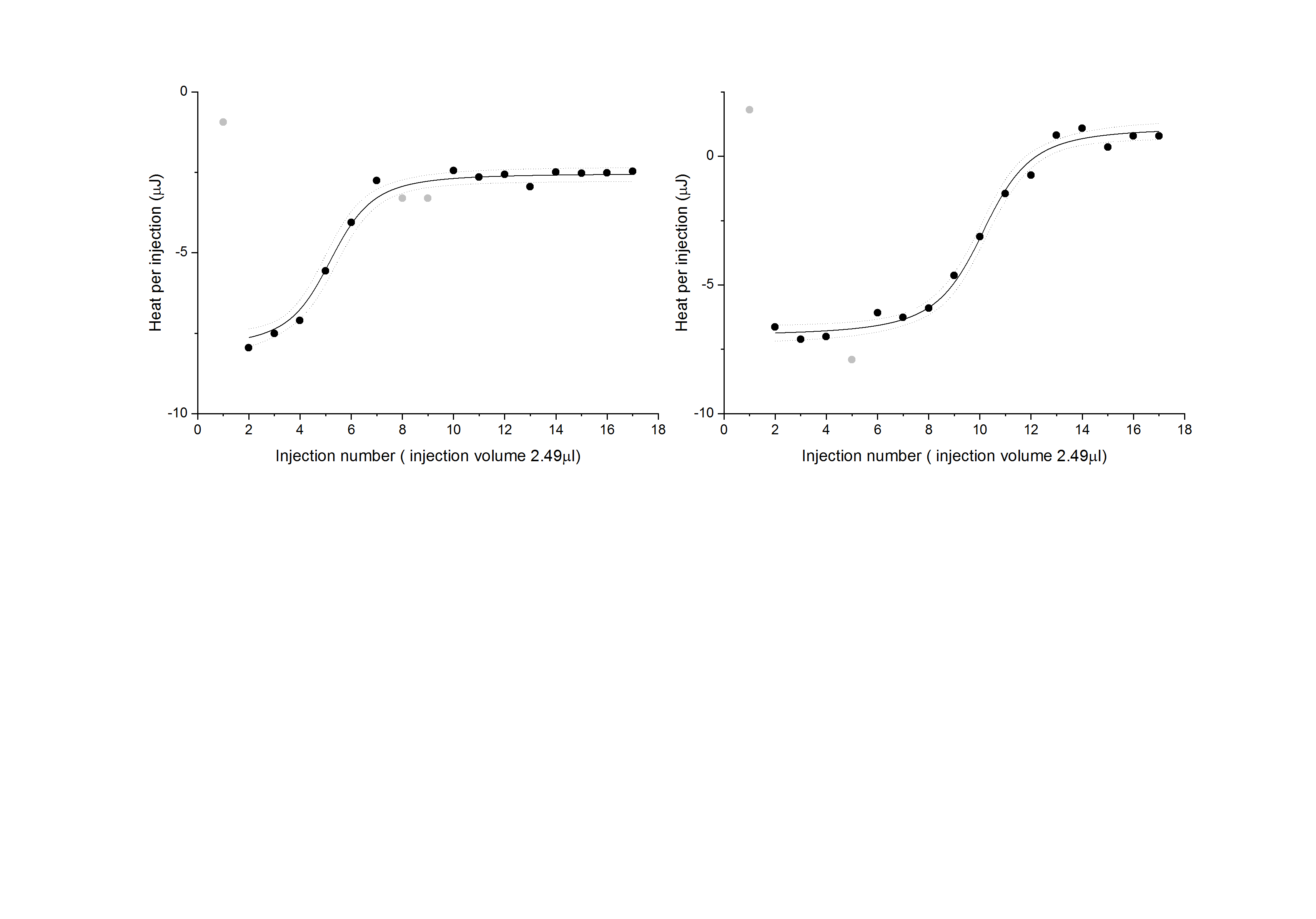
**

**Figure S35.** ITC titration data collected for **H115A hCK2α and 4,5,6-Br_3_Bt**. Black circles shows experimental data, grey ones indicate data removed from the analysis, thick line represents the fitted model of two independent biding sites and dotted ones boarder the 95% confidence limits for the model. First injection with volume 0.1μl was always removed from the analysis.

**
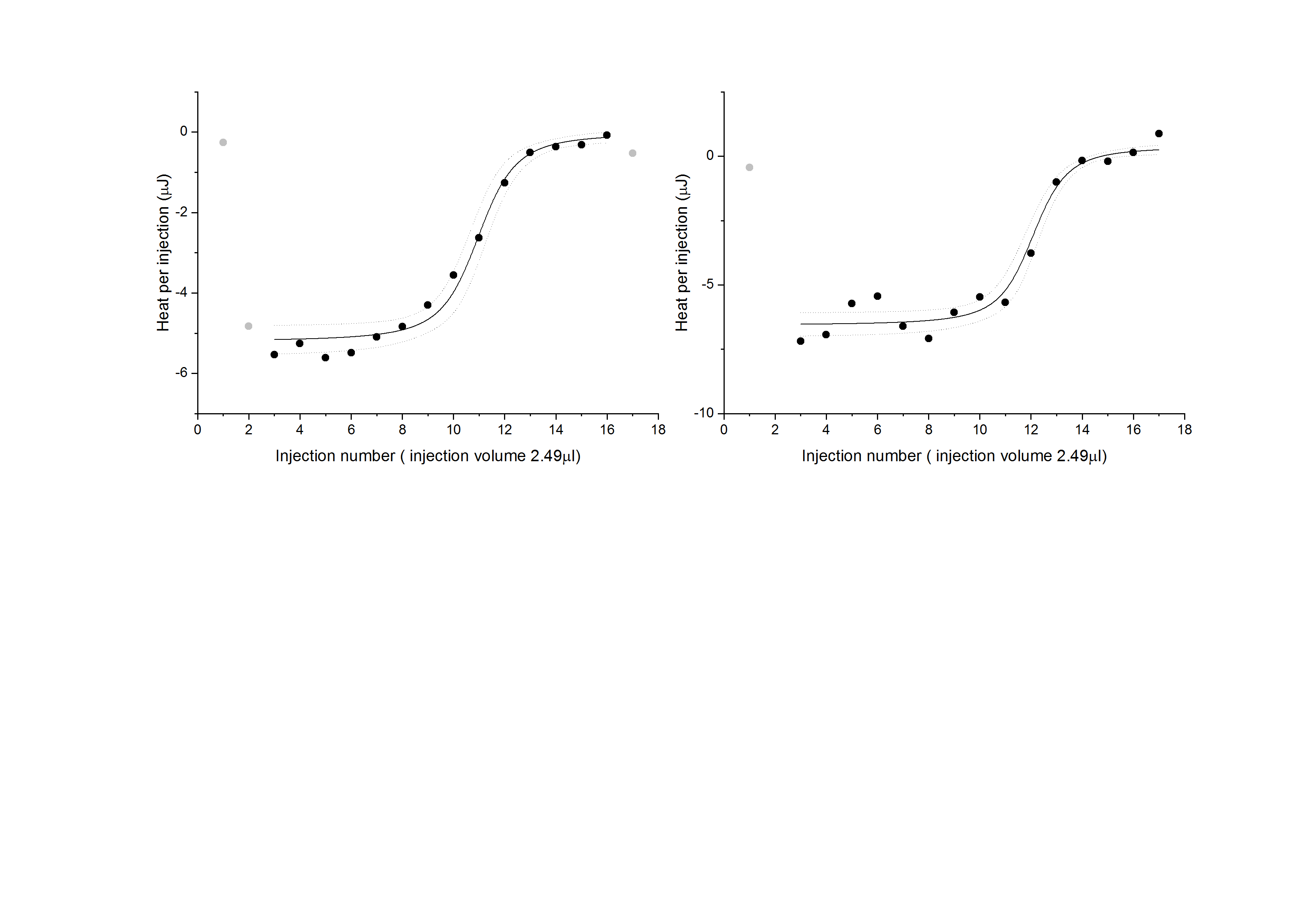
Figure S36.** ITC titration data collected for **H115A hCK2α and 5,6-Br_2_Bt**. Black circles shows experimental data, grey ones indicate data removed from the analysis, thick line represents the fitted model of two independent biding sites and dotted ones boarder the 95% confidence limits for the model. First injection with volume 0.1μl was always removed from the analysis.

**
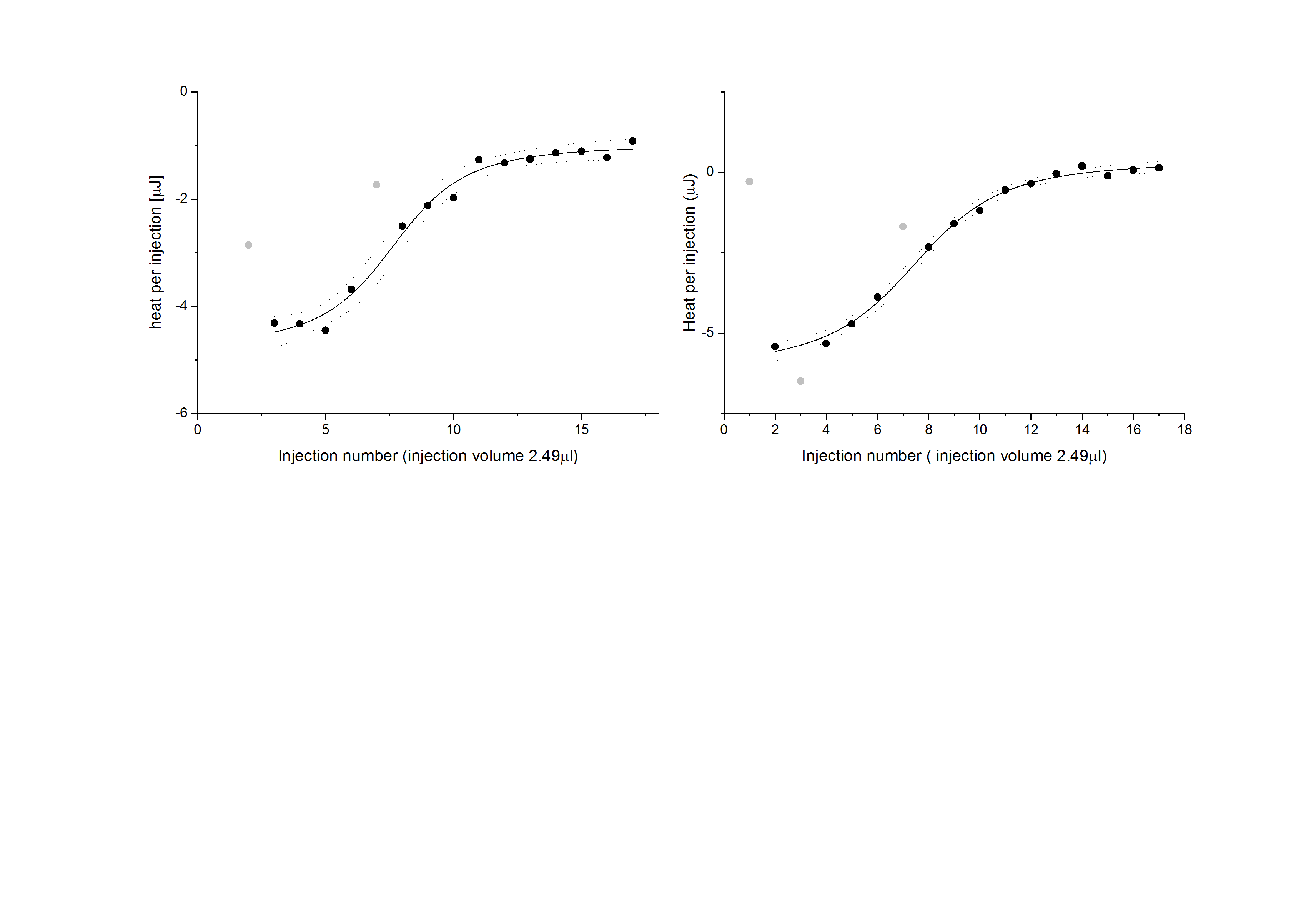
Figure S37.** ITC titration data collected for **H115A hCK2α and TBBz**. Black circles shows experimental data, grey ones indicate data removed from the analysis, thick line represents the fitted model of two independent biding sites and dotted ones boarder the 95% confidence limits for the model. First injection with volume 0.1μl was always removed from the analysis.

**
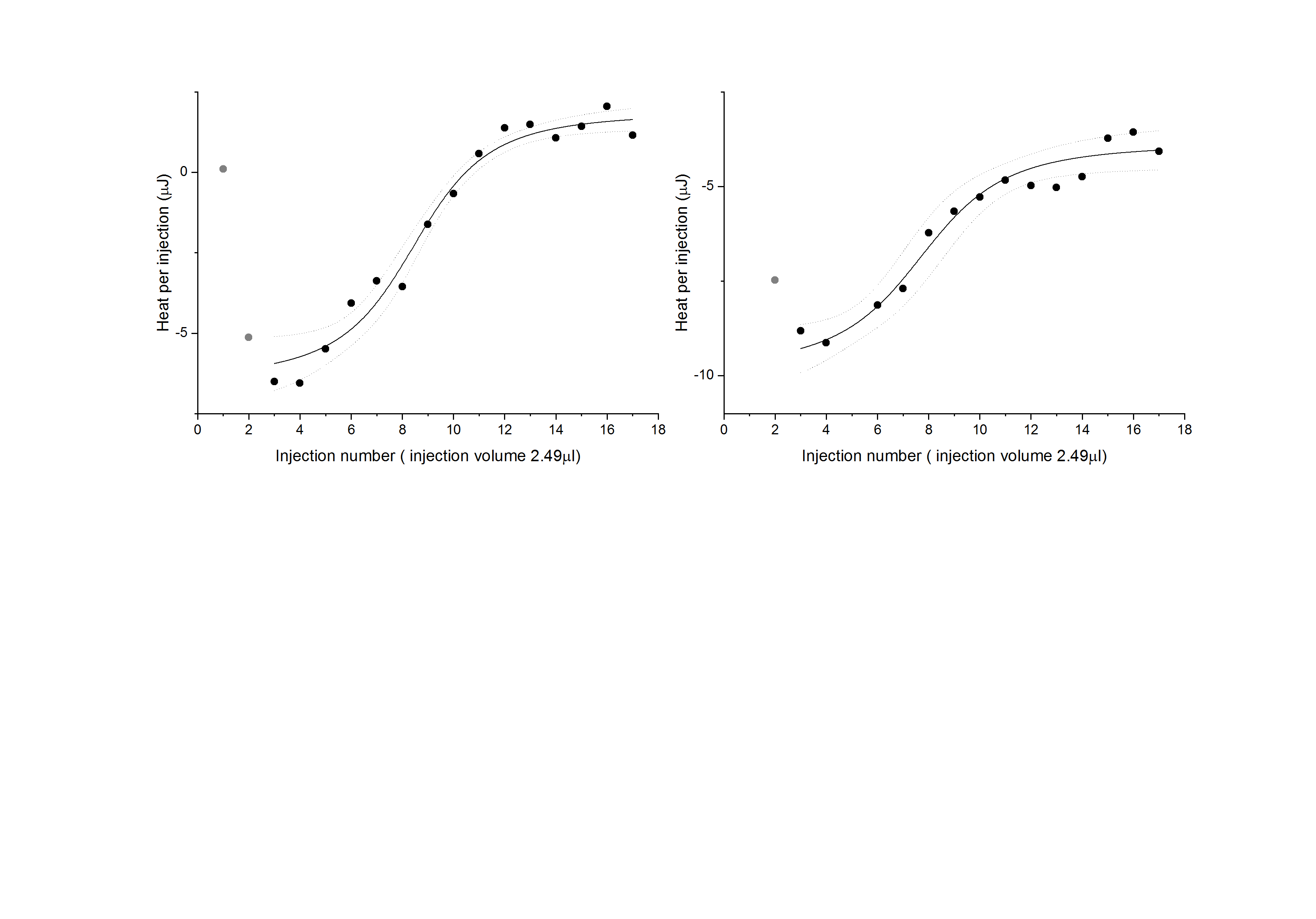
**

**Figure S38.** ITC titration data collected for **H115A hCK2α and 4,5,6-Br_3_Bz**. Black circles shows experimental data, grey ones indicate data removed from the analysis, thick line represents the fitted model of two independent biding sites and dotted ones boarder the 95% confidence limits for the model. First injection with volume 0.1μl was always removed from the analysis.

**
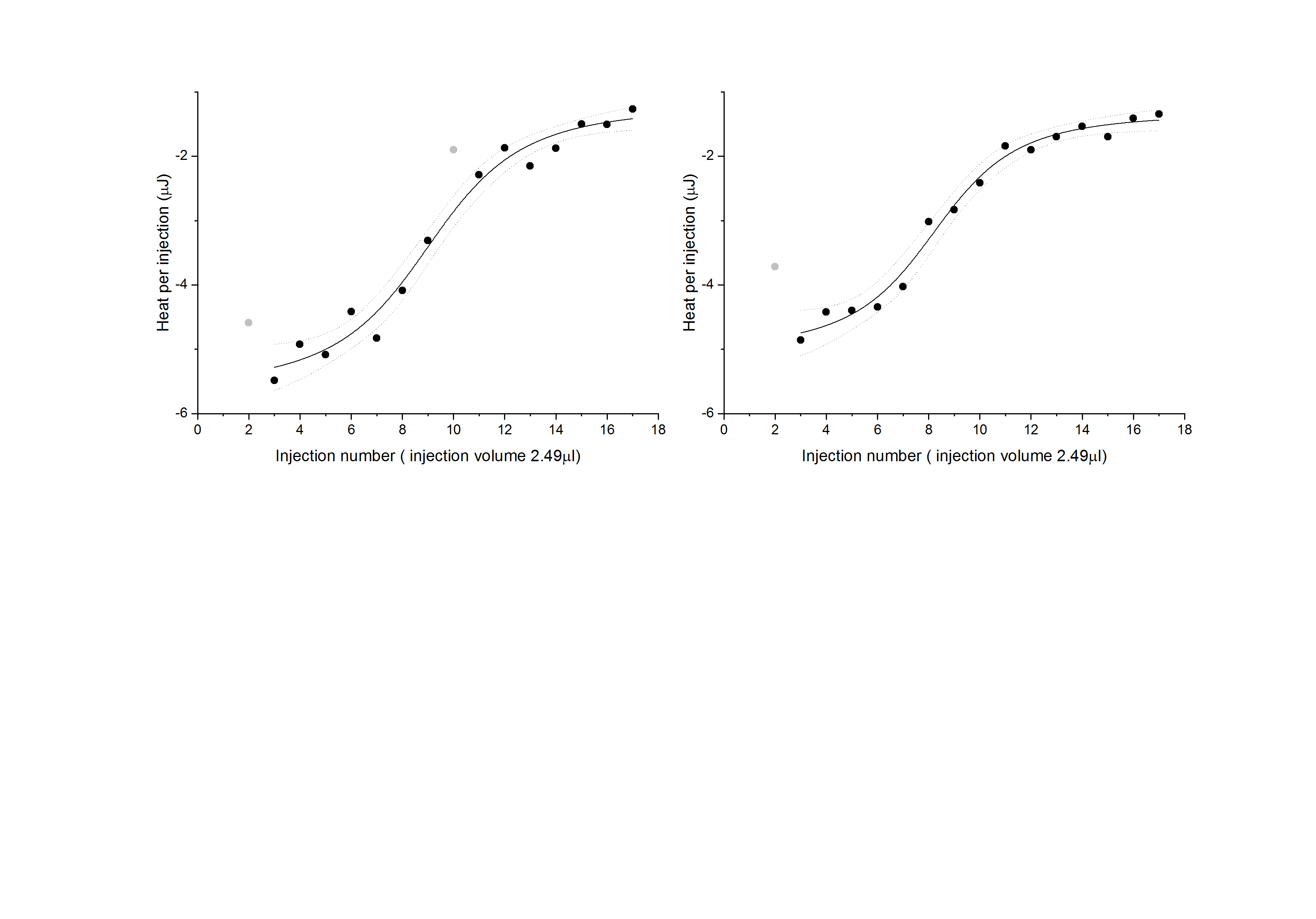
**

**Figure S396.** ITC titration data collected for **H115A hCK2α and 5,6-Br_2_Bz**. Black circles shows experimental data, grey ones indicate data removed from the analysis, thick line represents the fitted model of two independent biding sites and dotted ones boarder the 95% confidence limits for the model. First injection with volume 0.1μl was always removed from the analysis.

**
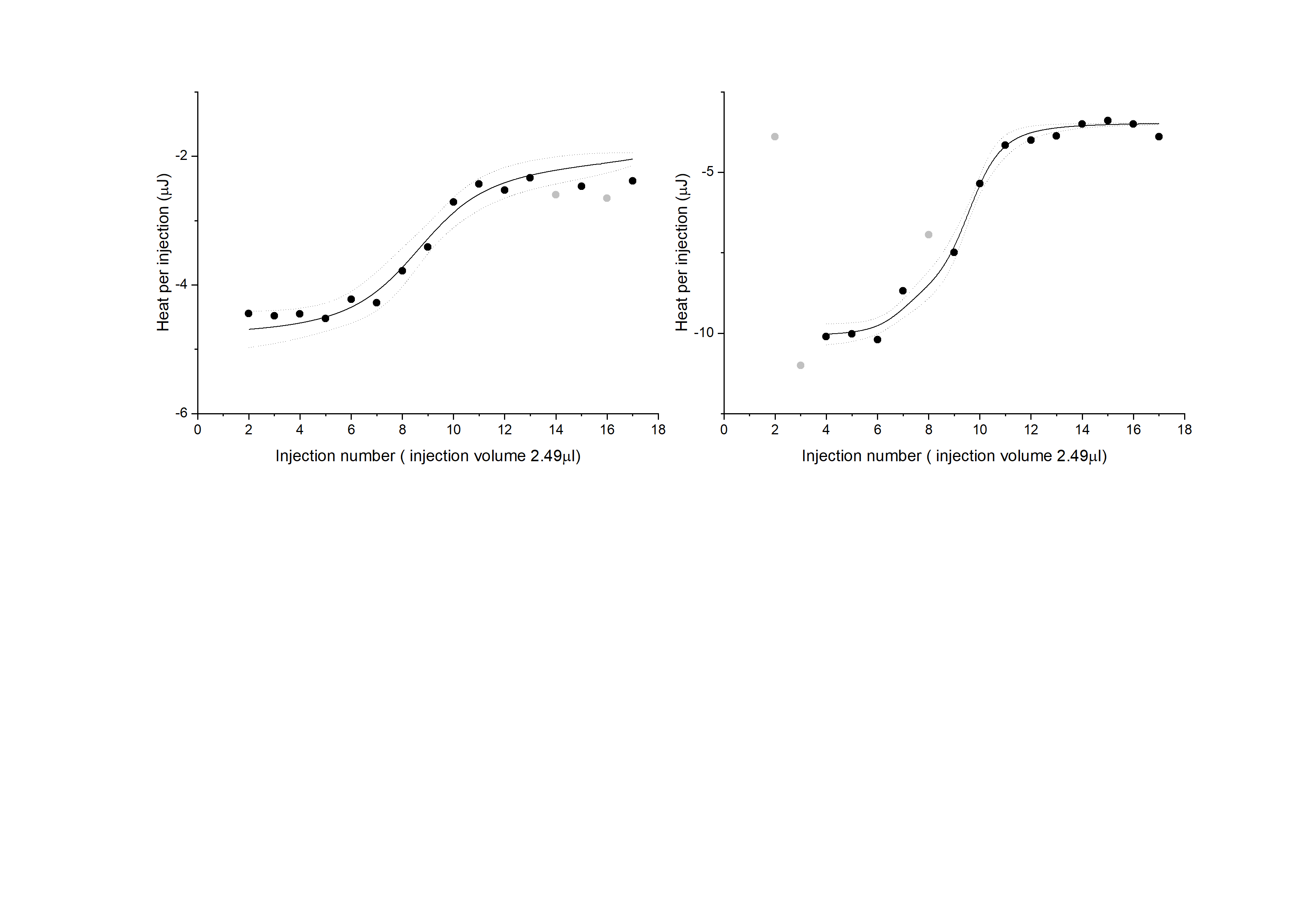
**

**Figure S40.** ITC titration data collected for **H160A hCK2α and TBBt**. Black circles shows experimental data, grey ones indicate data removed from the analysis, thick line represents the fitted model of two independent biding sites and dotted ones boarder the 95% confidence limits for the model. First injection with volume 0.1μl was always removed from the analysis.

**
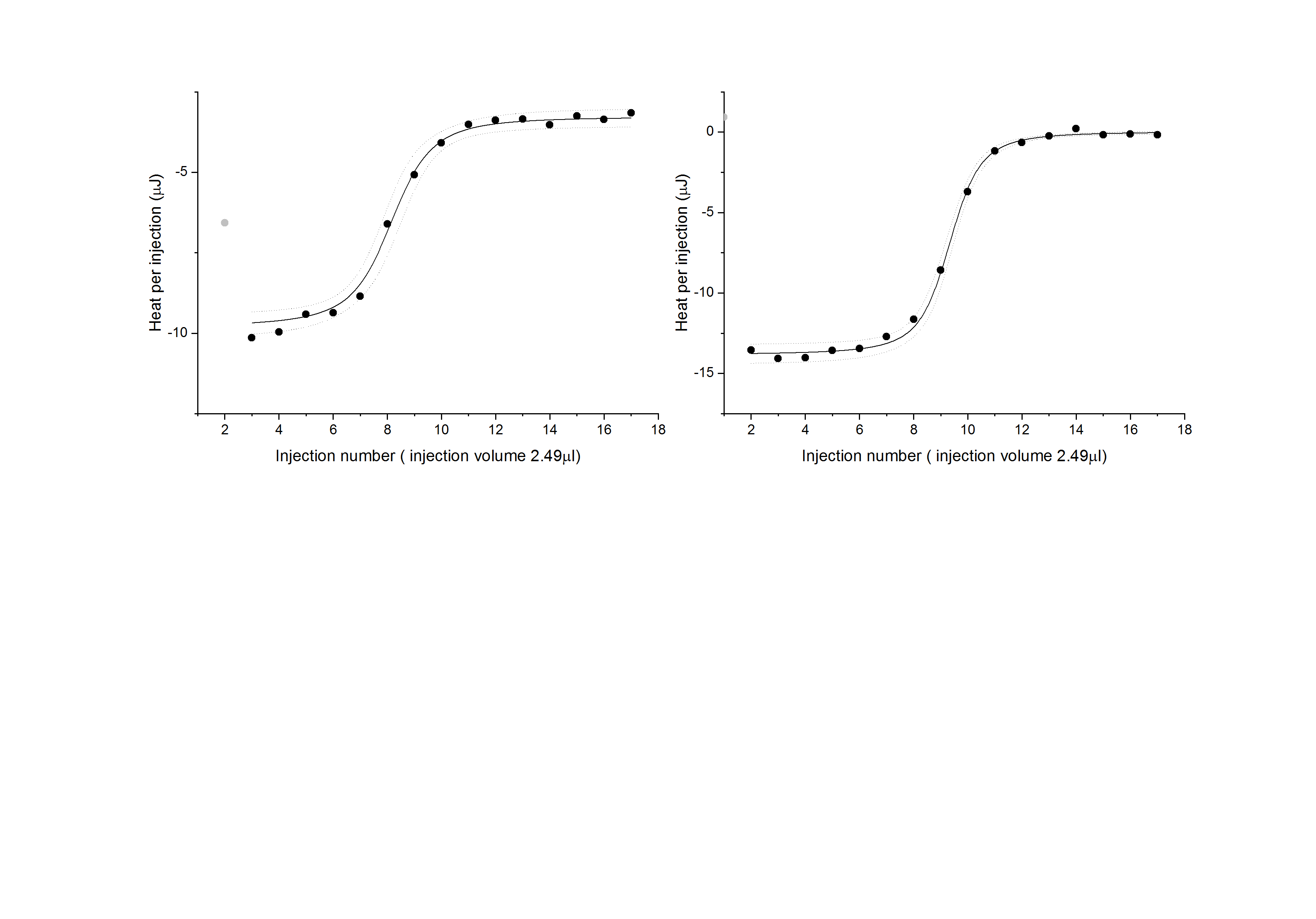
**

**Figure S41.** ITC titration data collected for **H160A hCK2α and 4,5,6-Br_3_Bt**. Black circles shows experimental data, grey ones indicate data removed from the analysis, thick line represents the fitted model of two independent biding sites and dotted ones boarder the 95% confidence limits for the model. First injection with volume 0.1μl was always removed from the analysis.

**
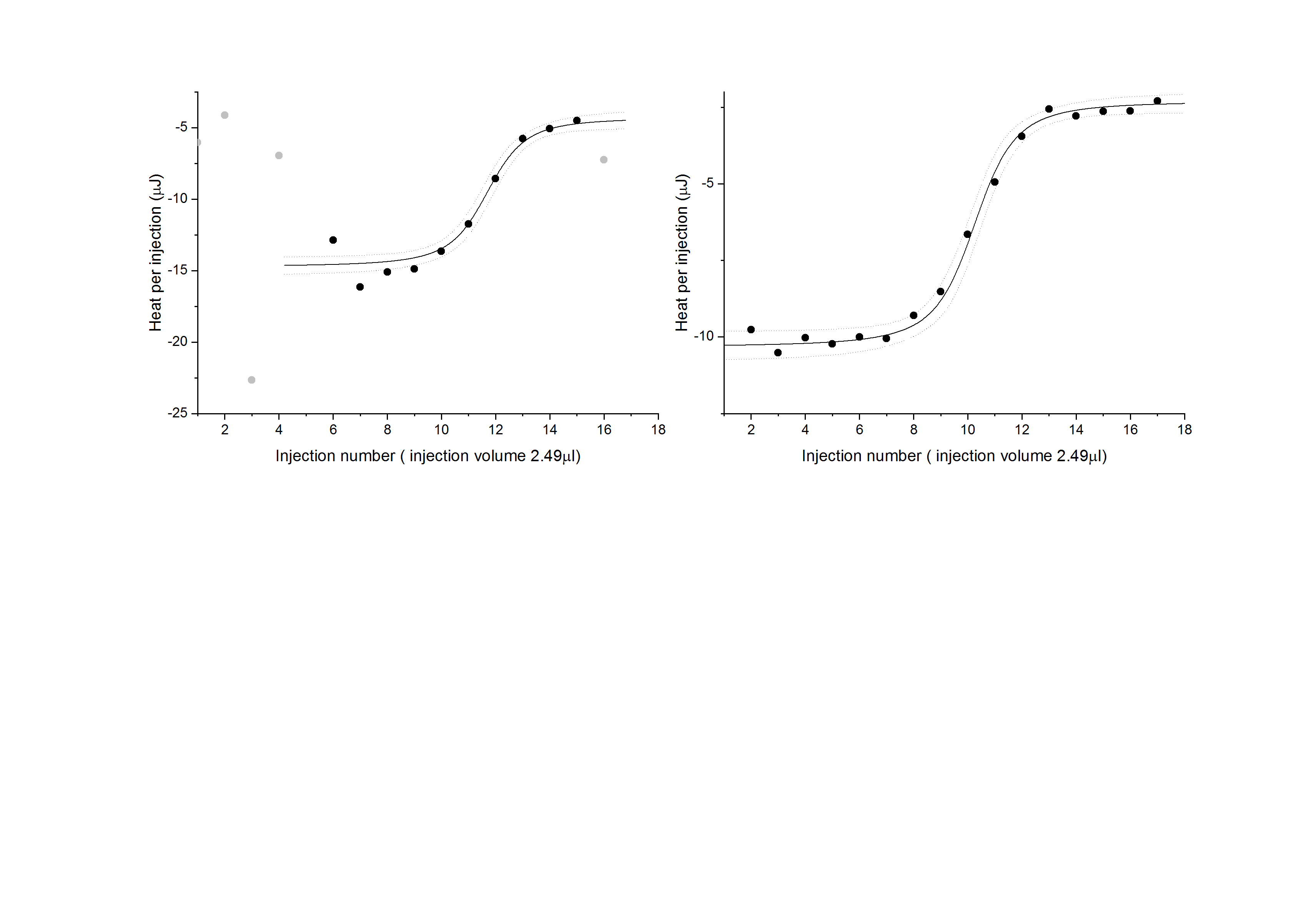
**

**Figure S42.** ITC titration data collected for **H160A hCK2α and 5,6-Br_2_Bt**. Black circles shows experimental data, grey ones indicate data removed from the analysis, thick line represents the fitted model of two independent biding sites and dotted ones boarder the 95% confidence limits for the model. First injection with volume 0.1μl was always removed from the analysis.

**
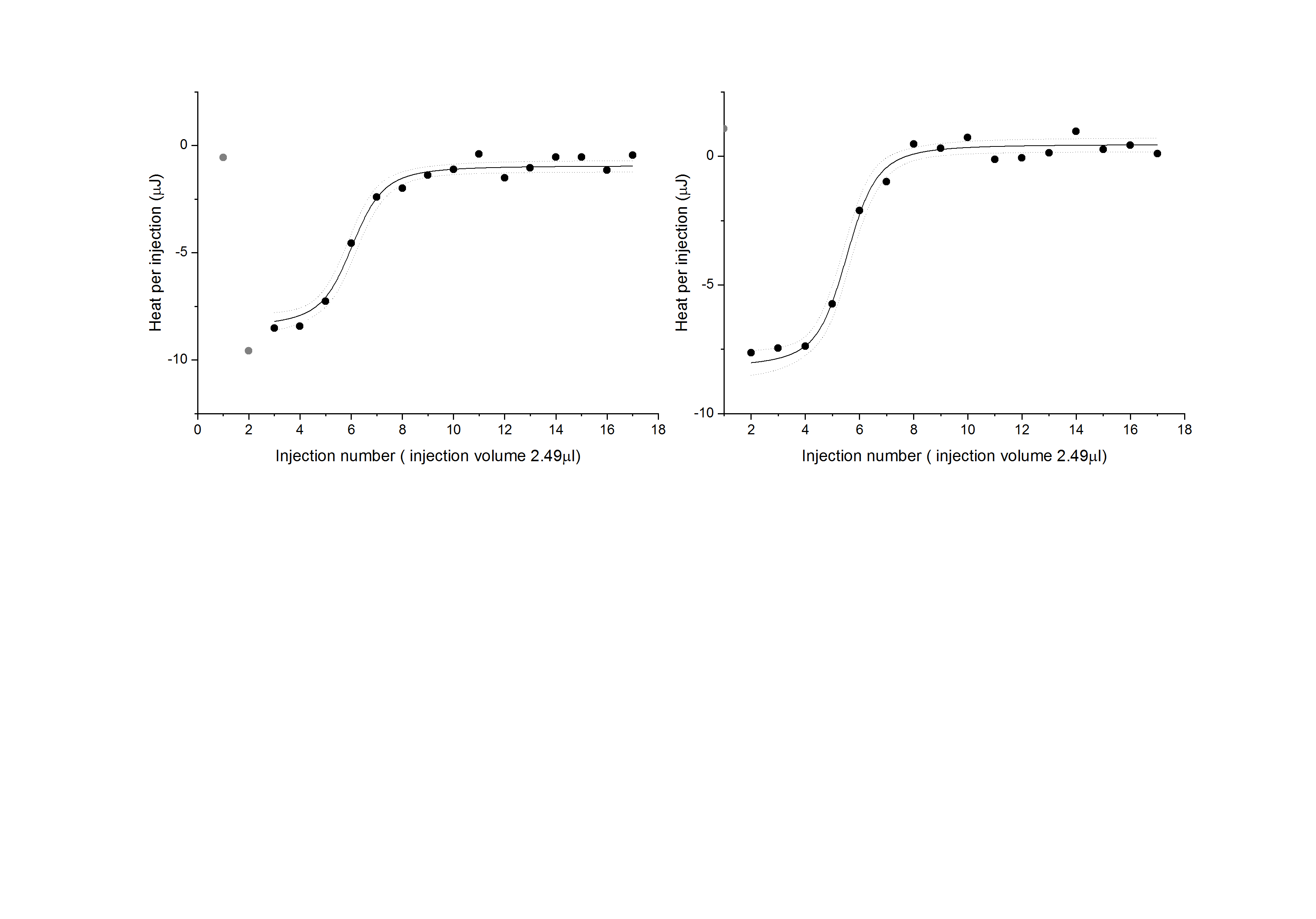
**

**Figure S43.** ITC titration data collected for **H160A hCK2α and TBBz**. Black circles shows experimental data, grey ones indicate data removed from the analysis, thick line represents the fitted model of two independent biding sites and dotted ones boarder the 95% confidence limits for the model. First injection with volume 0.1μl was always removed from the analysis.


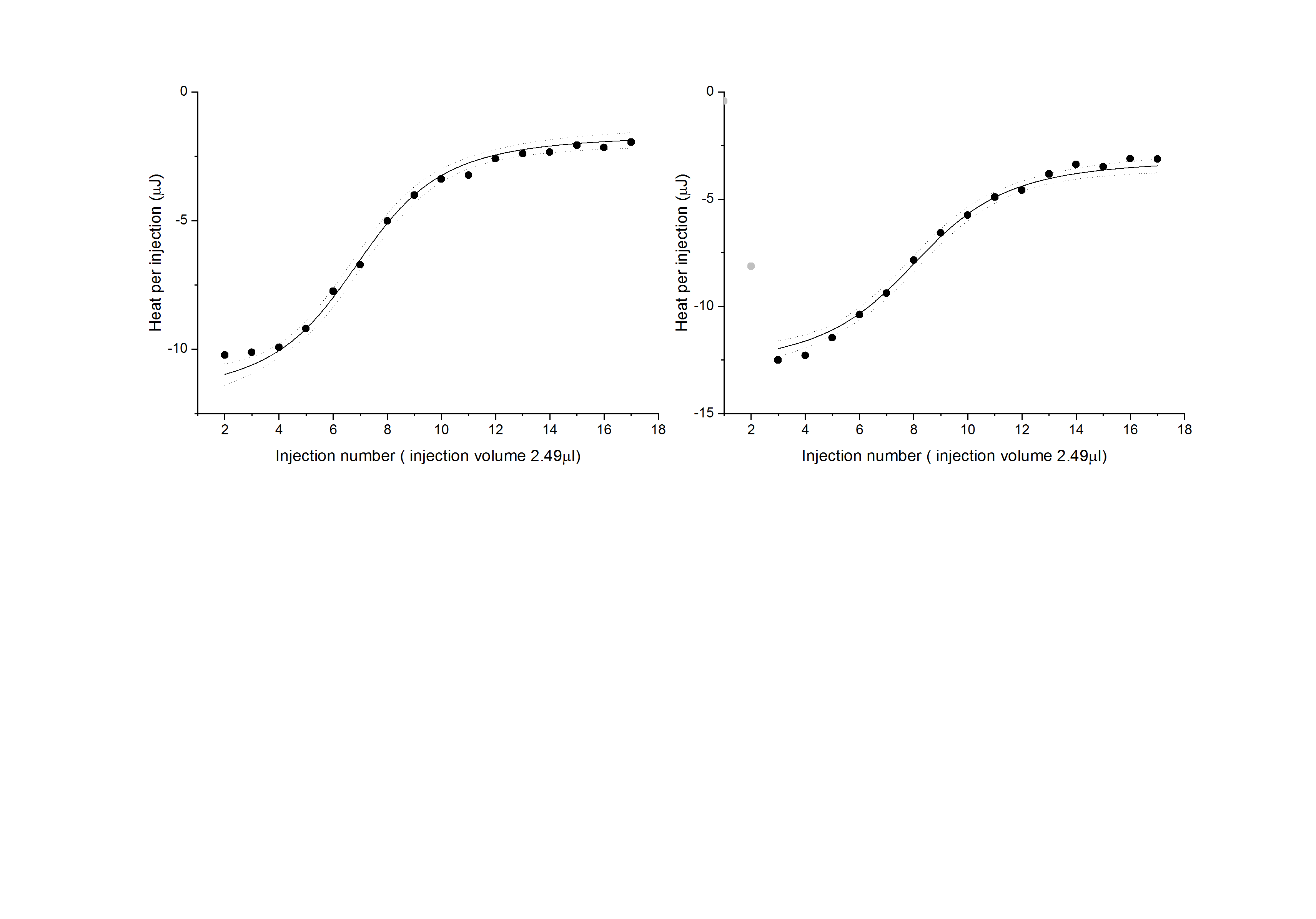


**Figure S44.** ITC titration data collected for **H160A hCK2α and 4,5,6-Br_3_Bz**. Black circles shows experimental data, grey ones indicate data removed from the analysis, thick line represents the fitted model of two independent biding sites and dotted ones boarder the 95% confidence limits for the model. First injection with volume 0.1μl was always removed from the analysis.

**
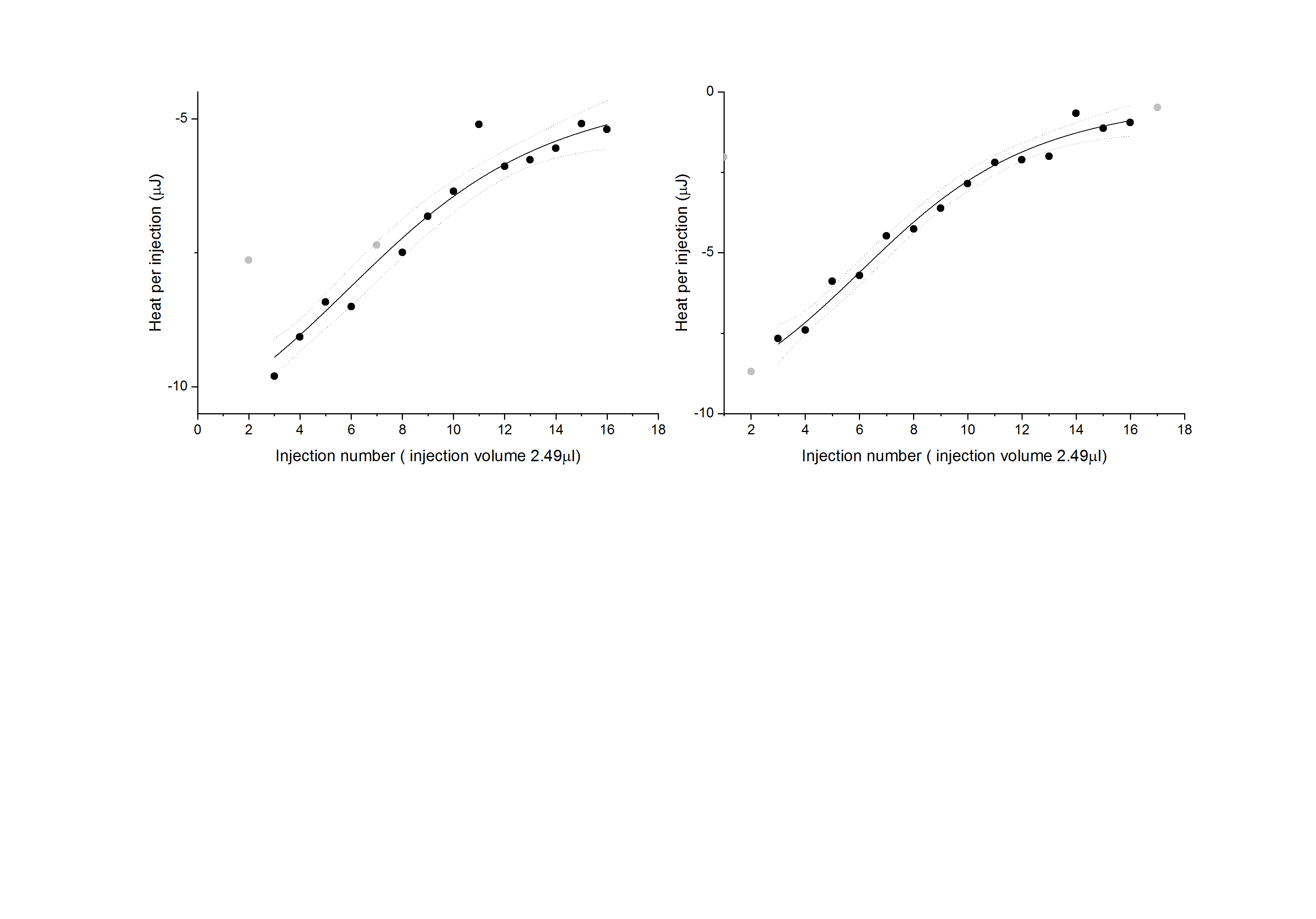
Figure S45.** ITC titration data collected for **H160A hCK2α and 5,6-Br_2_Bz**. Black circles shows experimental data, grey ones indicate data removed from the analysis, thick line represents the fitted model of two independent biding sites and dotted ones boarder the 95% confidence limits for the model. First injection with volume 0.1μl was always removed from the analysis.

**
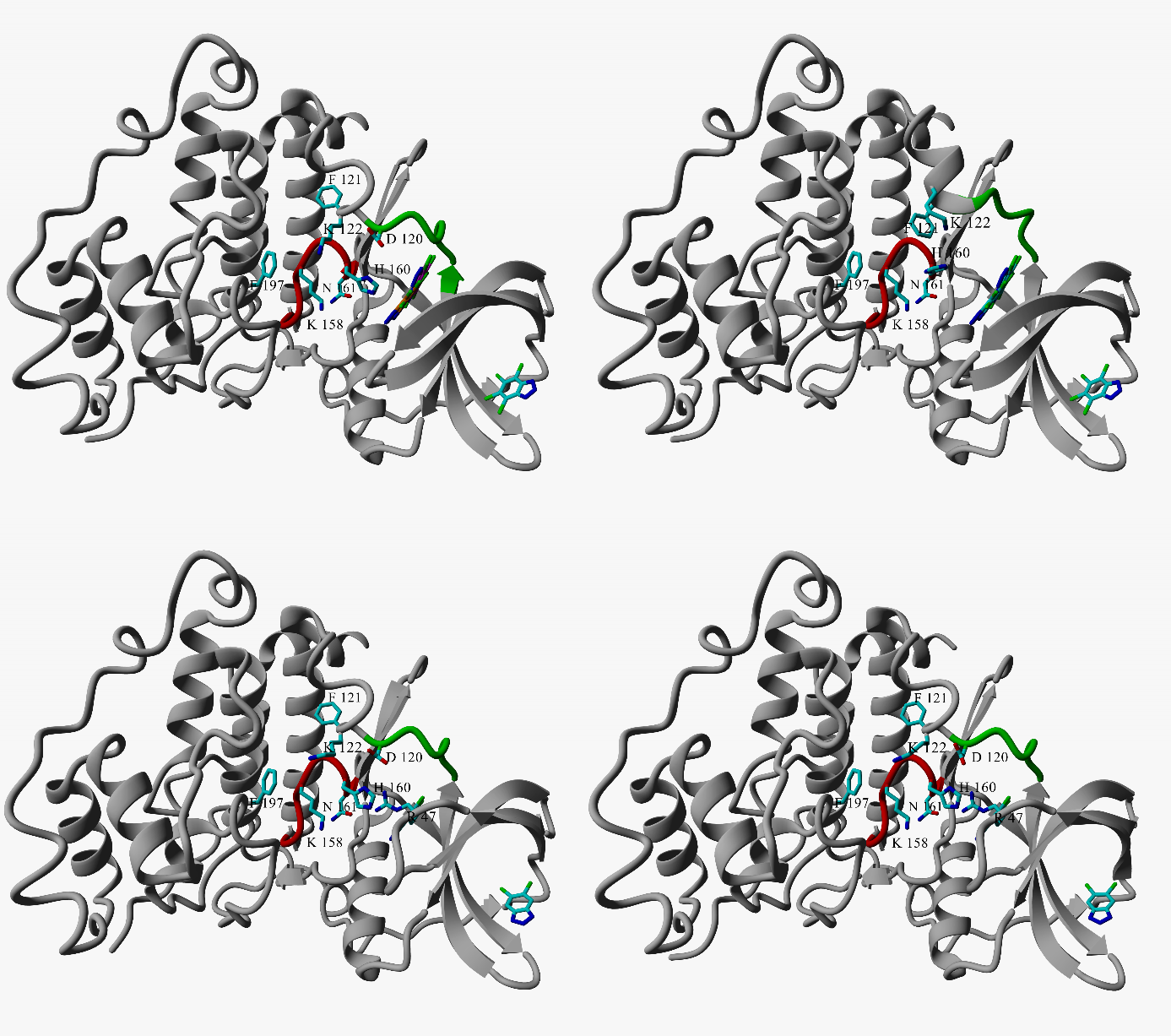
Figure S46. The interaction of His160 with the neighboring residues in hCK2α at different pH.** A) complex with TBBt at pH 7.5 (PDB:6TLL); B) complex with TBBt at pH 8.5 (PDB:7QGE); C) complex with 5,6-Br_2_Bt at pH 6.5 (PDB:7QGB) and D) complex with 5,6-Br_2_Bt at pH 8.5 (PDB:7QGD). The hinge region and the catalytic loop are denoted in green and red, respectively.

**A**

**C**

**D**

**B**
